# Visible light-mediated gold-catalysed carbon(sp^2^)–carbon(sp) cross-coupling†
†Electronic supplementary information (ESI) available. See DOI: 10.1039/c5sc03025k


**DOI:** 10.1039/c5sc03025k

**Published:** 2015-11-10

**Authors:** Suhong Kim, Jaime Rojas-Martin, F. Dean Toste

**Affiliations:** a Department of Chemistry , University of California , Berkeley , CA 94720 , USA . Email: fdtoste@berkeley.edu; b Departamento de Química Orgánica (Módulo 01) , Universidad Autónoma de Madrid , C/Francisco Tomásy Valiente 7 , Cantoblanco , 28049 Madrid , Spain

## Abstract

A dual photoredox and gold-catalysed cross-coupling reaction of alkynyltrimethylsilanes and aryldiazonium tetrafluoroborates is described. The reaction proceeds through visible-light mediated oxidative addition of aryldiazoniums, transmetalation of alkynyltrimethylsilanes and aryl–alkynyl reductive elimination. Exclusive selectivity for silyl-substituted alkynes is observed, with no reactivity observed for terminal alkynes.

## Abstract 2

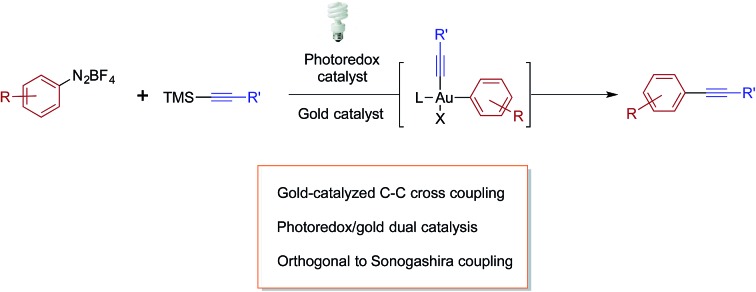
A new method for the alkynylation of aryldiazonium salts with TMS-alkynes *via* dual gold and photoredox catalysis is described.

## Introduction

**Scheme 1:**
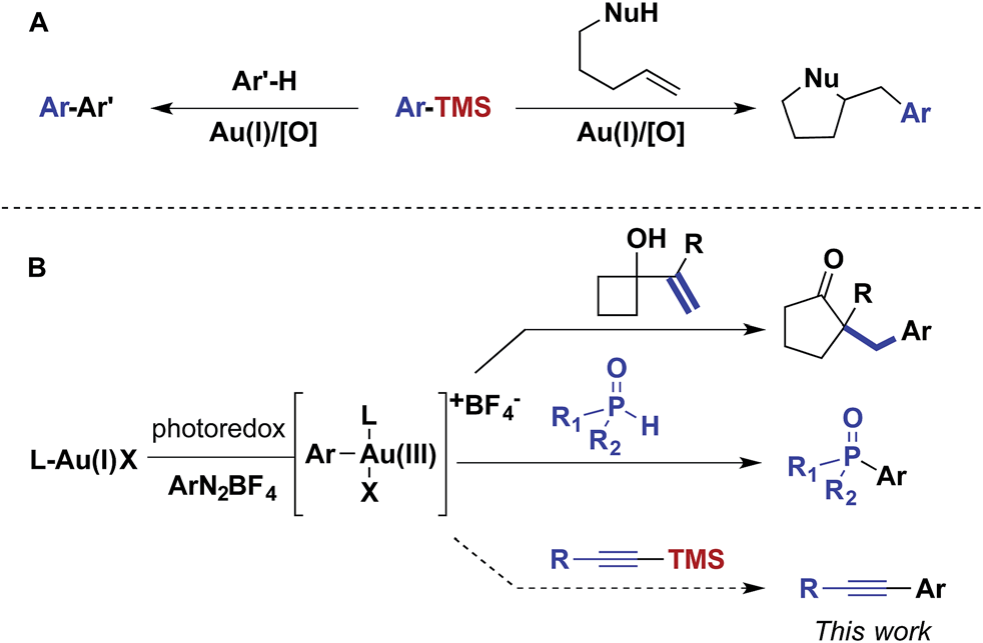
Photoredox catalyst and visible light-mediated Au^I^/Au^III^ catalysis.

For over a decade, homogeneous gold catalysis has been investigated because it provides access to novel modes of reactivity and enables rapid generation of complex molecular architectures.^1^ However, in contrast to other low-valent late transition metal catalysts, the majority of gold(i) complexes are unreactive towards the oxidative addition of aryl and vinyl halides and pseudohalides.^2^ Consequently, most gold-catalysed cross-coupling reactions have required sacrificial oxidants to access the +3 oxidation state.^3^ For example, this reactivity platform has been exploited in a gold-catalysed oxidative coupling of organosilanes and 1,2-alkene functionalizations (Scheme 1A). As an alternative, visible light-mediated oxidative addition of aryldiazoniums has emerged as a method for the generation of the requisite gold(iii) intermediates *via* photoredox catalysis, thereby obviating the need for sacrificial oxidants.^4^ These gold(iii) intermediates have been intercepted by a variety of nucleophiles in dual catalytic photoredox–gold reactions (Scheme 1B).

Although aryldiazoniums and photoredox catalysts provide an efficient method to generate gold(iii) intermediates, several challenges must be addressed in order to facilitate carbon–carbon cross-coupling *via* these species. First, aryldiazonium salts are highly electrophilic and reactive towards many classes of nucleophilic coupling partners. Aryldiazoniums are generally decomposed under Kumada and Negishi coupling conditions and do not tolerate ligand additives and stoichiometric bases employed in the majority of Suzuki, Sonogashira and Hiyama coupling reactions.^5^ Second, as visible light-mediated oxidative addition of aryldiazoniums is proposed to proceed through radical intermediates, nucleophilic coupling partners that can participate in single electron transfer, such as organotin and organotrifluoroborates compounds, are problematic.^6^ Inspired by the oxidative coupling of organosilanes, we envisioned that the gold(iii) complexes generated *via* photoredox catalysis might undergo transmetalation with organosilanes, generating an intermediate poised for carbon–carbon bond formation through reductive elimination.^7^,^8^

## Results and discussions

While aryldiazonium tetrafluoroborates have been shown to react with halo-, azido-, allyl- and thiotrimethylsilanes, their reactions with alkynyltrimethylsilanes has not been previously reported.^9^ Additionally, previous reports of organosilane transmetallation with gold lead us to hypothesize that the transmetallation of alkynyltrimethylsilanes to gold(iii) intermediates might result in productive cross-coupling. On the basis of this hypothesis, we examined the combined gold/photoredox coupling of 1 equivalent of aryldiazonium salt **1a** and 1 equivalent of alkynyltrimethylsilane **2**.

Evaluation of reaction conditions showed that the combination of Ar_3_PAuCl and Ru(bpy)_3_(PF_6_)_2_, in acetonitrile gave the highest yield of aryl alkyne **3a** (Table 1). Changing either the solvent or the photoredox catalyst proved detrimental to the yield of the desired product. With respect to the ligands on the gold catalyst, both electronic and steric factors impacted the efficiency of the cross-coupling. For example, electron-deficient triarylphosphine ligands resulted in significantly lower yield of **3a** (Table 1, entry 10). The sterics of the ligand showed an even more dramatic effect on reaction yield. The reaction conducted with (*p*-tol)_3_PAuCl provided 72% yield of the desired product, while the much more hindered (*o*-tol)_3_PAuCl-catalysed reaction only provided 17% yield of **3a** (Table 1, entry 11 and 12). The yield of product was lower when tricyclohexylphosphinegold(i) chloride was used as catalyst instead of triphenylphoshinegold(i) chloride (Table 1, entry 13). Gold(i) complexes of dialkylbiarylphosphine were also examined, but all showed less than 5% yield due to the combined effect of dialkyl groups and the steric effect of biaryl groups. Finally, no coupling product was observed in the absence of the ruthenium catalyst (entry 14) or when the reaction was conducted in the dark (entry 15).

**Table 1:** Optimization of reaction conditions

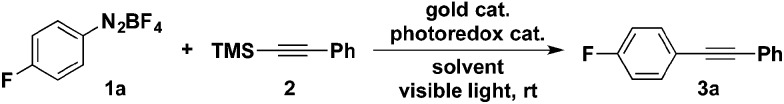				
Entry	Gold cat.	Photoredox cat.	Solvent	Yield^*a*^
1	Ph_3_PAuCl	Ru(bpy)_3_(PF_6_)_2_	CH_3_CN	70
2	Ph_3_PAuCl	Ru(bpy)_3_(PF_6_)_2_	Acetone	39
3	Ph_3_PAuCl	Ru(bpy)_3_(PF_6_)_2_	CH_3_NO_2_	15
4	Ph_3_PAuCl	Ru(bpy)_3_(PF_6_)_2_	DMF	10
5	Ph_3_PAuCl	Ru(bpy)_3_(PF_6_)_2_	MeOH	18
6	Ph_3_PAuCl	Ru(bpy)_3_(PF_6_)_2_	EtOH	4
7	Ph_3_PAuCl	Ir(ppy)_3_	CH_3_CN	21
8	Ph_3_PAuCl	Ir(ppy)_2_(dtbbpy)PF_6_	CH_3_CN	45
9	(*p*-MeOPh)_3_PAuCl	Ru(bpy)_3_(PF_6_)_2_	CH_3_CN	73
10	(*p*-CF_3_Ph)_3_PAuCl	Ru(bpy)_3_(PF_6_)_2_	CH_3_CN	43
11	(*p*-tol)_3_PAuCl	Ru(bpy)_3_(PF_6_)_2_	CH_3_CN	72
12	(*o*-tol)_3_PAuCl	Ru(bpy)_3_(PF_6_)_2_	CH_3_CN	17
13	Cy_3_PAuCl	Ru(bpy)_3_(PF_6_)_2_	CH_3_CN	48
14	Ph_3_PAuCl	—	CH_3_CN	0
15^*b*^	Ph_3_PAuCl	Ru(bpy)_3_(PF_6_)_2_	CH_3_CN	0

^*a*^GC yields.

^*b*^The reaction was run in the dark. bpy = 2,2′-bipyridine, ppy = 2-phenylpyridine, dtbbpy = 4,4′-di-*tert*-butyl-2,2′-bipyridine.

With an optimized catalyst system in hand, the scope with respect to the aryldiazonium coupling partner was examined (Table 2). The yields of alkynylation reactions of electron-poor aryldiazonium salts were generally higher than those of reactions with electron-rich aryldiazonium coupling partners. It should be noted that the intrinsic instability of *ortho*-substituted aryldiazonium salts limited their use in this transformation (Table 2, entry **3k** and **3l**). It is also noteworthy that all halogen substitutions on the aryldiazonium coupling partner were preserved during the coupling reaction. Bromo- and iodoaryldiazonium salts were readily coupled and leaving halides intact for use in further reactions (Table 2, **3b** and **3j**); this chemoselectivity is challenging under typical palladium-catalysed Sonogashira coupling reactions.^10^

**Table 2:** Scope of aryldiazonium tetrafluoroborates

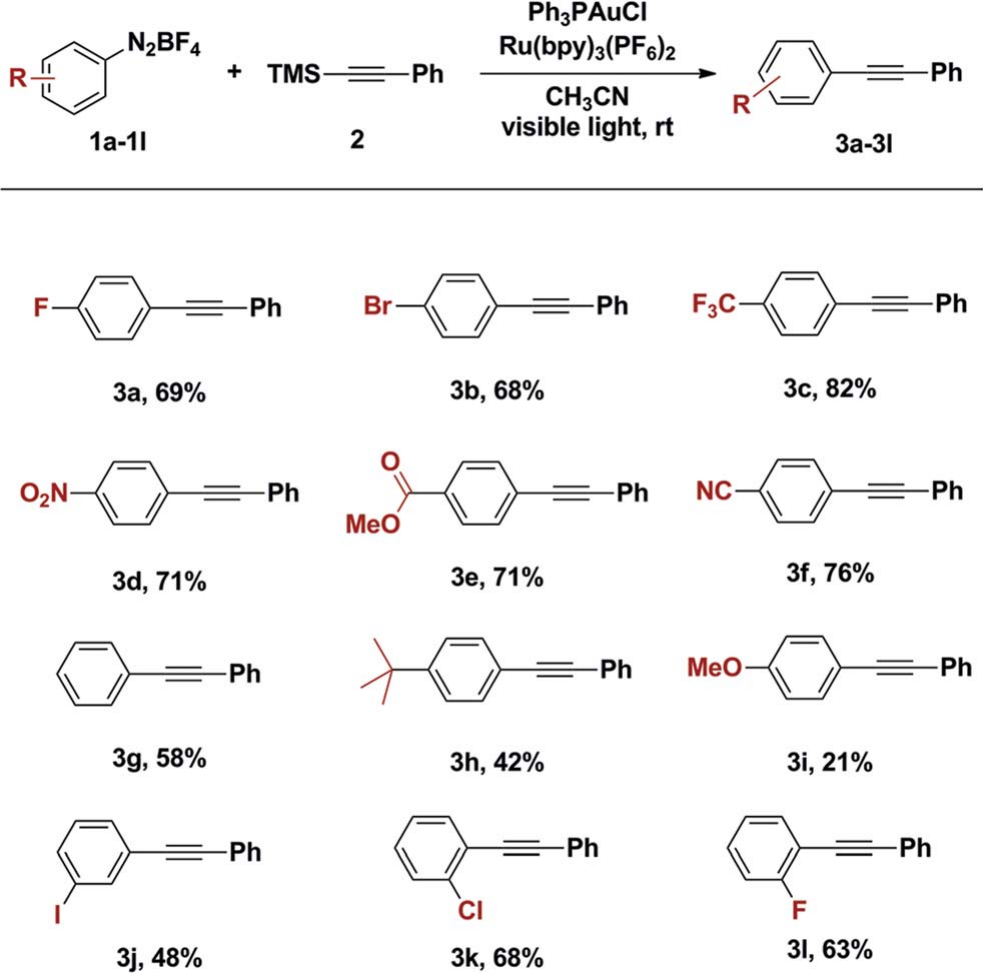

The difference in reactivity of electron-rich and -poor aryldiazoniums may be the result of competing reaction pathways. Oxidative addition is believed to proceed through single electron reduction of aryldiazoniums and this process is preferred with electron-poor aryldiazonium salts.^4^ On the other hand, the generation of aryl cations, through loss of dinitrogen, is facile with electron-rich aryldiazoniums. Correspondingly, we observed trace amount of desired product when benzenediazonium tetrafluoroborate was heated at 60 °C for 9 hours in the presence of 1-trimethylsilyl-2-(4-methoxy)phenylacetylene; however the same reactivity was not observed with 4-fluorophenyldiazonium salts on the same conditions.

The scope of alkynyltrimethylsilanes was also investigated (Table 3). Both aryl- and alkylethynyltrimethylsilanes were coupled with modest to high product yields. *o*-Isopropylphenyl, biphenylyl and naphthylalkynyltrimethylsilanes participated in the gold-catalysed coupling (Table 3, **3m**, **3n** and **3o**). Potentially sensitive benzylic and propargylic C–H and C–X bonds were well tolerated under the reaction conditions (Table 3, **3q**, **3aa** and **3s–v**). Additionally, alkynyltrimethylsilane **3w** was prepared in 78% yield from the coupling of aryldiazonium salt **1d** and bis(trimethylsilyl)acetylene, providing a compliment to using trimethylsilylacetylene in a traditional Sonogashira coupling (Table 3, **3w**).

**Table 3:** Scope of alkynyltrimethylsilanes

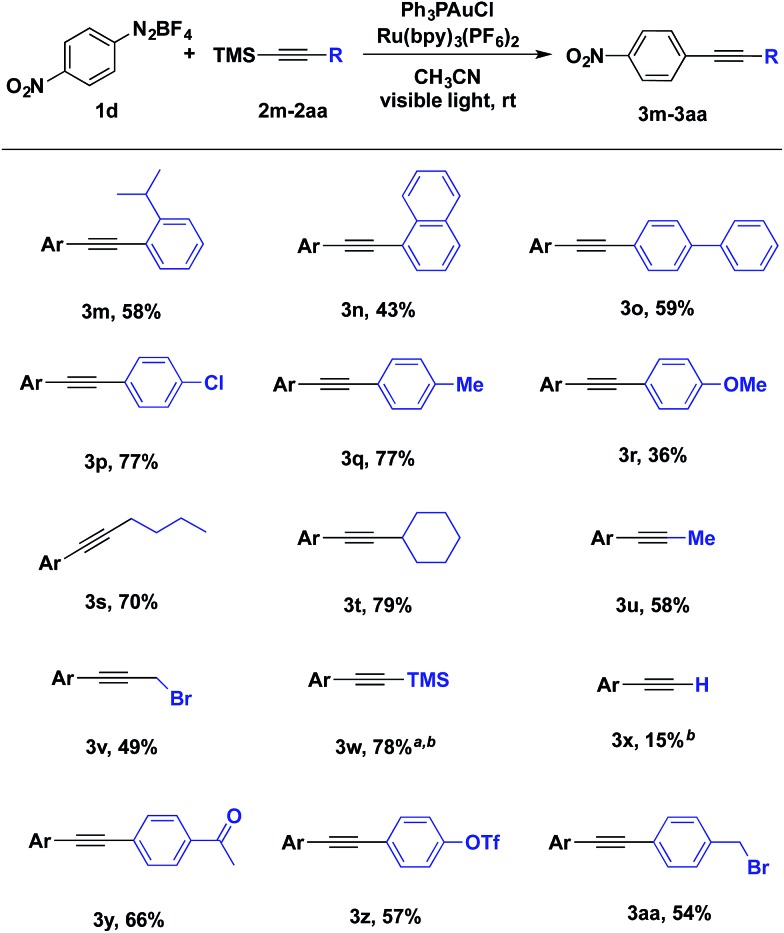

^*a*^5 equiv. of bis(trimethylsilyl)acetylene was used with 1 equiv. of **1d**.

^*b*^GC yields.

Another interesting feature of this reaction is the effect of the silyl group on yield (Table 4). When trimethylsilylacetylene was coupled with **1d** to generate terminal alkyne **3x**, the yield was 15% with none of the corresponding silylacetylene **3w** (Table 3). This observation suggests that, under the current base free reaction conditions, the silyl group plays a critical role in the coupling. To investigate this role, a set of reactions was performed by varying counteranions of aryldiazonium salts and silyl group identities of alkynylsilanes. The yield of coupling product was diminished as the steric hindrance of silyl group was increased (Table 4, entry 1 and entries 4–7). While both tetrafluoroborate and tosylate diazonium salts proved efficient coupling partners, the yield was diminished when hexafluorophosphate salts were used.^11^ Moreover, when the corresponding terminal alkyne was used instead of alkynyltrimethylsilanes, only 3% of the coupling product was observed (Table 4, entry 8).^12^ Taken together, these results suggests that transmetallation from the organosilane is critical for high efficiency of the current coupling reaction.

**Table 4:** Effect of counteranion and silyl group

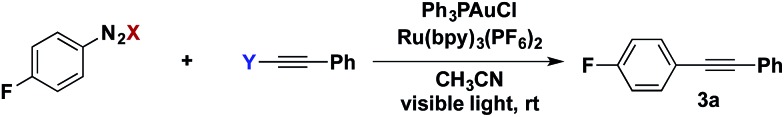			
Entry	Counteranion (**X**)	Silyl group (**Y**)	Yield^*a*^
1	BF_4_^–^	TMS	70
2	TsO^–^	TMS	68
3	PF_6_^–^	TMS	24
4	BF_4_^–^	TES	61
5	BF_4_^–^	TBDMS	10
6	BF_4_^–^	TIPS	10
7	BF_4_^–^	TBDPS	1
**8**	**BF**_**4**_^**–**^	**H**	**3**

^*a*^GC yields.

**Scheme 2:**
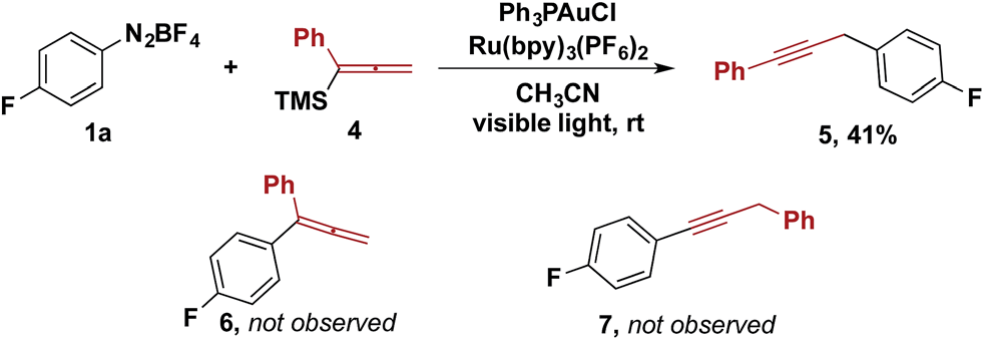
Reaction with allenyltrimethylsilanes.

Other organotrimethylsilanes were tested under the optimized reaction conditions. While the reactions of aryl and vinylsilanes were complicated by competing reactions,^13^,^14^ the gold/photoredox-catalysed coupling of allenyltrimethylsilane **4** provided propargylic compound **5** as the sole coupling product (Scheme 2). The product from aryl–allenyl reductive elimination and the other aryl–propargylic coupling isomer were prepared independently (Scheme 2, **6** and **7**). We examined whether **6** was an intermediate that underwent isomerization to **5** facilitated by visible light and the photoredox catalyst. However, no isomerization was observed of **6** to either **5** or **7** under the reaction conditions. Therefore, the formation of **5** is best rationalized by transmetalation of **4** to the gold(iii) intermediate, followed by 1,3-migration and aryl–propargyl reductive elimination.

## Conclusions

In summary, we have demonstrated that trimethylsilylalkynes and aryldiazonium tetrafluoroborates can be coupled *via* dual photoredox and gold catalysis strategy. The reaction proceeds under mild conditions and shows excellent functional group tolerance, including aryl halides that may be reactive under traditional coupling conditions. This process compliments the Sonogashira coupling, especially when aryl–alkynyl coupling is required under non-basic conditions and may present an advantage when TMS-alkynes are available rather than corresponding terminal alkynes.

## Supplementary Material

Supplementary information
