# Supplementary material for: Visible light-mediated gold-catalysed carbon(sp2)–carbon(sp) cross-coupling
Source: Chem Sci. 2015 Nov 10;7(1):85–8. doi: 10.1039/c5sc03025k (PMC5947751; doi:10.1039/c5sc03025k)
Supplement: Supplementary file 1 [file SC-007-C5SC03025K-s001.pdf]

Electronic Supplementary Material (ESI) for Chemical Science.
This journal is © The Royal Society of Chemistry 2015

Supporting Information

General consideration	S2
Preparation of aryldiazonium salts	S3
Preparation of alkynylsilanes	S6
Preparation of gold catalyst and photoredox catalysts	S12
General procedure for gold-catalyzed aryl-akynyl cross coupling	S13
Preparation of *p*-nitrophenylacetylene, **3x**	S21
Preparation of 1-phenyl-3-*p*-fluorophenyl-1-propyne, **5**	S21
Preparation of 1-phenyl-1-*p*-fluorophenylprop-1,2-diene, **6**	S22
Preparation of 1-p-fluorophenyl-3-phenyl-1-propyne, **7**	S22
References	S23
Spectra	S25

General consideration

Unless otherwise noted, reagents were purchased from commercial suppliers and used without further purification. All magnetic stir bars were washed with freshly prepared aqua regia prior to use to minimize the potential for trace metal contamination. Column chromatography was performed using Sorbent Technology silica gel, (60 Å, 30-63 μm). Thin-layer chromatography analysis was performed using Merck silica gel 60 F254 TLC plates, and visualized by iodine, *p*-anisaldehyde, ceric ammonium molybdate, vanillin or UV lamp. All NMR spectra were recorded with Bruker AV-300, AVB-400, AVQ-400, AV-500, DRX-500 and AV-600 spectrometers. ^{1}H and ^{13}C chemical shifts were reported in ppm downfield of tetramethylsilane and referenced to residual solvent peak ($CHCl_3$; δH = 7.26 and δC = 77.00, CH_3CN; δH = 1.94 and δC = 1.32, 118.26, acetone; δH = 2.05 and δC = 29.84, 206.26, DMSO; δH = 2.50 and δC = 39.52). ^{19}F chemical shifts were reported in ppm upfield of $CFCl_3$ and referenced to internal standard (C_6F_6; δH = −164.9). Multiplicities were reported using the following abbreviations: s = singlet, d = doublet, t = triplet, q = quartet, m = multiplet. Gas chromatography was conducted on an HP 6850A Series GC System on a Chiraldex 225 β-dex column with a flame ionization detector. (*E*)-Stilbene was used as an internal standard. Mass spectral data were obtained from the Autospec Premier magnetic sector mass spectrometer (EI) at the Micro-Mass/Analytical Facility operated by the College of Chemistry, University of California, Berkeley.

The gold-catalyzed carbon-carbon cross coupling were run in 2 dram (17 mm x 60 mm) vials (not dried) fitted with a screw cap and stirring was carried out using a 10 mm magnetic stir bar. All reactions were carried out under air unless otherwise noted. Acetonitrile, diethyl ether, tetrahydrofuran, dimethyl formamide, and triethylamine were purified by passage through an activated alumina column under argon. A household lamp with a 23 W fluorescent light bulb and a 17 W white LED was used for the light source of reactions. **Caution:** Appropriate safety precautions should be taken due to the explosive nature of diazonium salts, including the use of a blast shield.

Preparation of aryldiazonium salts

General procedure for aryldiazonium tetrafluoroborate preparation[1]

The aniline (10 mmol) was dissolved in a mixture of 50% (V/V) fluoroboric acid (3.5 mL) and water (3.5 mL). After cooling to 0 °C, an aqueous solution of sodium nitrite (700 mg, 10.1 mmol, in 1.5 mL H_2O) was added in 0.25 mL portions. The mixture was stirred for 30 min and the thick precipitate was collected by filtration and dissolved in minimal amount of acetone. The diazonium tetrafluoroborate was then precipitated by the addition of Et_2O. The product was dried under high vacuum for several hours.

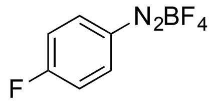

p-Fluorobenzenediazonium tetrafluoroborate, **1a**

White solid (1.36 g, 65%), ^1H NMR (400 MHz, CD_3CN) δ 8.62 – 8.59 (m, 2H), 7.68 – 7.64 (m, 2H). In accordance with previously reported spectra[2]

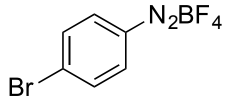

p-Bromobenzenediazonium tetrafluoroborate, **1b**

Pale pink solid (2.11 g, 78%), ^1H NMR (400 MHz, CD_3CN) δ 8.37 (d, *J* = 8.8 Hz, 2H), 8.10 (d, *J* = 8.9 Hz, 2H). In accordance with previously reported spectra.[3]

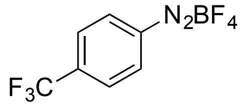

p-Trifluoromethylbenzenediazonium tetrafluoroborate, **1c**

White solid (1.66 g, 64%), ^1H NMR (400 MHz, CD_3CN) δ 8.71 (d, *J* = 8.6 Hz, 2H), 8.23 (d, *J* = 8.6 Hz, 2H). In accordance with previously reported spectra.[4]

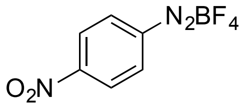

p-Nitrobenzenediazonium tetrafluoroborate, **1d**

White solid (1.73 g, 73%), ^{1}H NMR (400 MHz, CD_3CN) δ 8.77 (d, *J* = 9.0 Hz, 2H), 8.63 (d, *J* = 9.0 Hz, 2H). In accordance with previously reported spectra.[3]

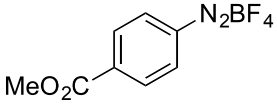

p-Methoxycarbonylbenzenediazonium tetrafluoroborate, **1e**

White solid (1.46 g, 58%), ^{1}H NMR (400 MHz, CD_3CN) δ 8.61 (d, *J* = 8.6 Hz, 2H), 8.41 (d, *J* = 8.6 Hz, 2H), 3.97 (s, 3H). In accordance with previously reported spectra.[2]

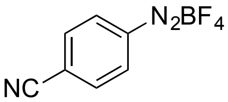

p-Cyanobenzenediazonium tetrafluoroborate, **1f**

White solid (1.83 g, 85%), ^{1}H NMR (400 MHz, CD_3CN) δ 8.64 (d, *J* = 9.0 Hz, 1H), 8.23 (d, *J* = 9.0 Hz, 1H). In accordance with previously reported spectra.[5]

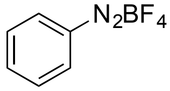

Benzenediazonium tetrafluoroborate, **1g**

White solid (1.37 g, 71%), ^{1}H NMR (400 MHz, CD_3CN) δ 8.50 (d, *J* = 8.2 Hz, 2H), 8.25 (t, *J* = 7.6 Hz, 1H), 7.92 (t, *J* = 7.5 Hz, 2H). In accordance with previously reported spectra.[3]

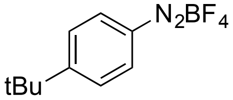

p-*tert*-Butylbenzenediazonium tetrafluoroborate, **1h**

White solid (0.915 g, 37%), ^{1}H NMR (400 MHz, CD_3CN) δ 8.42 (d, *J* = 9.1 Hz, 2H), 7.97 (d, *J* = 9.0 Hz, 2H), 1.38 (s, 1H). In accordance with previously reported spectra.[3]

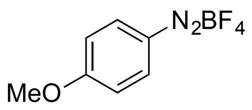

p-Methoxybenzenediazonium tetrafluoroborate, **1i**

Beige solid (1.59 g, 72%), ^{1}H NMR (400 MHz, CD_3CN) δ 8.43 (d, *J* = 9.4 Hz, 2H), 7.35 (d, *J* = 9.4 Hz, 2H), 4.05 (s, 3H). In accordance with previously reported spectra.[2]

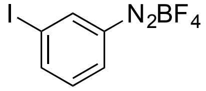

m-Iodobenzenediazonium tetrafluoroborate, **1j**

Beige solid (1.34 g, 42%), ^{1}H NMR (400 MHz, CD_3CN) δ 8.77 (s, 1H), 8.62 – 8.41 (m, 2H), 7.68 – 7.64 (m, 1H).

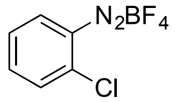

o-Chlorobenzenediazonium tetrafluoroborate, **1k**

White solid (1.72 g, 76%), ^{1}H NMR (400 MHz, CD_3CN) δ 8.70 – 8.50 (m, 1H), 8.33 – 8.16 (m, 1H), 8.02 – 8.00 (m, 1H), 7.90 – 7.86 (m, 1H).

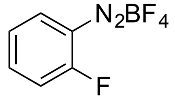

o-Fluorobenzenediazonium tetrafluoroborate, **1l**

White solid (1.81 g, 87%), ^{1}H NMR (400 MHz, CD_3CN) δ 8.54 – 8.50 (m, 1H), 8.37 – 8.31 (m, 1H), 7.79 – 7.73 (m, 2H).

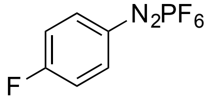

Proecdure for *p*-fluorobenzenediazonium hexafluorophosphate preparation[6]

p-Fluoroaniline (35.0 mmol) was dissolved in a mixture of 12N aqueous HCl solution (9.5 mL) and water (65 mL). After cooling to 0 °C, an aqueous solution of sodium nitrite (2.9 g, 42.0 mmol, in 7.5 mL H_2O) was added in 1.5 mL portions. 65% aqueous HPF_6 (7.4 mL, 13.4 g, 60

mmol) solution was added in one portion with vigorous stirring. The mixture was stirred for 30 min and the precipitate was collected by filtration. The precipitate was dissolved in acetone and then recrystallized by the addition of diethyl ether. Again, the precipitate was dissolved in methanol and recrystallized by the addition of diethyl ether. Colorless solid (5.77 g, 62%), ^{1}H NMR (400 MHz, CD_3CN) δ 8.59 – 8.54 (m, 2H), 7.71 – 7.61 (m, 2H).

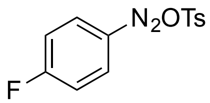

Procedure for p-fluorobenzenediazonium tosylate preparation[7]

p-Fluoroaniline (10.0 mmol) was dissolved in a mixture of 50% (V/V) glacial acetic acid (50 mL) and water (50 mL). *p*-Toluenesulfonic acid dihydrate (11.0 mmol) was added to the solution. After cooling to 0 °C, *tert*-butylnitrite (19.5 mmol) was added dropwise to the solution. The mixture was stirred for 30 min and stirred at 23 °C for another 30 min. Diethyl ether (500 mL) was poured into the solution and the precipitate was collected by filtration. The precipitate was dissolved in a minimal amount of methanol and then precipitated by the addition of diethyl ether. The product was dried under high vacuum for several hours. Colorless solid (2.64 g, 90%), ^{1}H NMR (400 MHz, DMSO-d_6) δ 8.86 – 8.78 (m, 2H), 7.91 – 7.87 (m, 2H), 7.47 (d, J = 8.0 Hz, 2H), 7.11 (d, J = 8.0 Hz, 2H), 2.28 (s, 3H).

<u>Preparation of alkynylsilanes</u>

General procedure for phenylethynylsilanes preparation[8]

Phenylacetylene (20.4 mmol) was dissolved in THF (60 mL). After cooling to –78 °C, *n*-BuLi (2.5M in hexanes, 8.3 mL, 20.8 mmol) was added dropwise to the solution. The mixture was stirred for 2 h at –78 °C. The silylchloride (20.0 mmol) dissolved in THF (10 mL) was added dropwise. The mixture was stirred 6 h at 23 °C. A saturated solution of aqueous ammonium chloride (100 mL) was added and the mixture was extracted with dichloromethane (2 × 100 mL). The collected organic layer was washed with a mixture of 50% (V/V) brine (100 mL) and water (100 mL), then dried over $MgSO_4$, filtered and concentrated under reduced pressure. The product was purified by flash column chromatography.

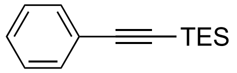

(Phenylethynyl)triethylsilane. Colorless liquid (2.96 g, 68%), ^{1}H NMR (400 MHz, $CDCl_3$) δ 7.53 – 7.49 (m, 2H), 7.33 – 7.32 (m, 3H), 1.10 (t, *J* = 7.9 Hz, 9H), 0.72 (q, *J* = 7.9 Hz, 6H), ^{13}C NMR (101 MHz, $CDCl_3$) δ 131.86, 128.18, 127.98 123.16, 106.20, 91.36, 7.34, 4.27. In accordance with previously reported spectra.[9]

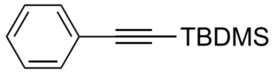

(Phenylethynyl)-*tert*-butyldimethylsilane. Colorless liquid (2.75 g, 64%), ^{1}H NMR (400 MHz, $CDCl_3$) δ 7.54 – 7.51 (m, 2H), 7.35 – 7.33 (m, 3H), 1.07 (s, 9H), 0.25 (s, 6H), ^{13}C NMR (101 MHz, $CDCl_3$) δ 131.97, 128.40, 128.16, 123.24, 105.78, 92.36, 26.15, 16.72, -4.58. In accordance with previously reported spectra.[10]

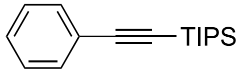

(Phenylethynyl)triisopropylsilane. Colorless liquid (4.09 g, 79%), ^{1}H NMR (400 MHz, $CDCl_3$) δ 7.56 – 7.54 (m, 2H), 7.38 – 7.31 (m, 3H), 1.22 (s, 21H), ^{13}C NMR (101 MHz, $CDCl_3$) δ 132.01, 128.25, 128.14, 123.57, 107.15, 90.36, 18.67, 11.34. In accordance with previously reported spectra.[11]

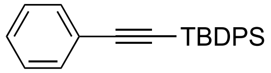

(Phenylethynyl)-*tert*-butyldiphenylsilane. Colorless liquid (2.89 g, 42%), ^{1}H NMR (400 MHz, $CDCl_3$) δ 8.03 – 8.00 (m, 4H), 7.76 – 7.69 (m, 2H), 7.54 – 7.50 (m, 6H), 7.47 – 7.42 (m, 3H), 1.30 (s, 9H), ^{13}C NMR (101 MHz, $CDCl_3$) δ 135.58, 133.27, 132.11, 129.50, 128.81, 128.26, 127.71, 122.96, 109.14, 89.29, 27.11, 18.71, m/z HRMS (EI) found 340.1646 $C_{24}H_{24}Si$ requires 340.1647.

General Procedure for alkynyltrimethylsilane[12]

Iodobenzene (4.0 mmol, 1 equiv.), $Pd(PPh_3)_2Cl_2$ (84 mg, 0.120 mmol, 3 mol%), CuI (46 mg, 0.242 mmol, 6 mol%) and triethylamine (1.63 g, 16.1 mmol, 4 equiv.) were dissolved in DMF

(6.8 mL). Trimethylsilylacetylene (0.39 g, 4.00 mmol, 1 equiv.) was added to the mixture. After heating to 60°C, the mixture was stirred for 1 h. The mixture was diluted with a mixture of 50% (V/V) ethyl acetate (50 mL) and water (50 mL). The organic layer was collected and washed with water (3 × 50 mL) and brine (50 mL). The organic layer was dried over MgSO4, filtered and concentrated under reduced pressure. The residue was purified by flash chromatography on silica gel.

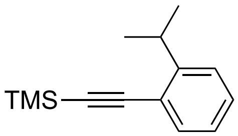

((*o*-Isopropylphenyl)ethynyl)trimethylsilane, **2m**

Yellow liquid (726 mg, 84%), ^{1}H NMR (500 MHz, $CDCl_3$) δ 7.44 (d, *J* = 7.7 Hz, 1H), 7.32 – 7.23 (m, 2H), 7.13 – 7.10 (m, 1H), 3.46 (hept, *J* = 6.9 Hz, 1H), 1.27 (d, *J* = 6.9 Hz, 6H), 0.27 (s, 9H), ^{13}C NMR (126 MHz, $CDCl_3$) δ 150.91, 132.55, 128.79, 125.36, 124.79, 121.87, 103.95, 97.98, 31.50, 22.87, -0.02. In accordance with previously reported spectra.[13]

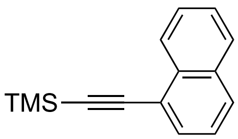

((1-Naphthyl)ethynyl)trimethylsilane, **2n**

Yellow liquid (731 mg, 81%), ^{1}H NMR (400 MHz, $CDCl_3$) δ 8.37 – 8.34 (m, 1H), 7.89 – 7.79 (m, 2H), 7.72 (dd, *J* = 7.2, 1.2 Hz, 1H), 7.59 (ddd, *J* = 8.3, 6.9, 1.4 Hz, 1H), 7.52 (ddd, *J* = 8.1, 6.8, 1.3 Hz, 1H), 7.42 (dd, *J* = 8.3, 7.1 Hz, 1H), 0.35 (s, 9H), ^{13}C NMR (101 MHz, $CDCl_3$) δ 133.37, 133.05, 130.78, 128.95, 128.23, 126.80, 126.36, 126.17, 125.09, 120.71, 103.03, 99.41, 0.11. In accordance with previously reported spectra.[12]

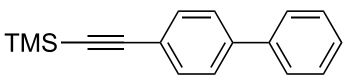

([1,1'-Biphenyl]-4-ylethynyl)trimethylsilane, **2o**

White solid (755 mg, 75%), ^{1}H NMR (400 MHz, $CDCl_3$) δ 7.65 – 7.53 (m, 6H), 7.48 – 7.44 (m, 2H), 7.41 – 7.34 (m, 1H), 0.31 (s, 9H), ^{13}C NMR (101 MHz, $CDCl_3$) δ 141.15, 140.28, 132.37,

128.81, 127.62, 126.99, 126.83, 122.02, 105.00, 94.83, 0.00. In accordance with previously reported spectra.[14]

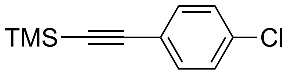

((p-Chlorophenyl)ethynyl)trimethylsilane, **2p**

White solid (690 mg, 83%), ^{1}H NMR (400 MHz, $CDCl_3$) δ 7.38 (d, J = 8.5 Hz, 2H), 7.27 (d, J = 8.5 Hz, 2H), 0.24 (s, 9H), ^{13}C NMR (101 MHz, $CDCl_3$) δ 134.47, 133.16, 128.52, 121.59, 103.79, 95.32, -0.12. In accordance with previously reported spectra.[15]

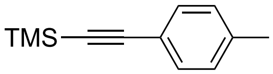

((p-Tolyl)ethynyl)trimethylsilane, **2q**

Yellow liquid (653 mg, 87%), ^{1}H NMR (400 MHz, $CDCl_3$) δ 7.36 (d, J = 8.1 Hz, 2H), 7.10 (d, J = 7.8 Hz, 2H), 2.34 (s, 3H), 0.25 (s, 9H), ^{13}C NMR (101 MHz, $CDCl_3$) δ 138.58, 131.83, 128.92, 120.01, 105.33, 93.20, 21.49, 0.02. In accordance with previously reported spetra.[16]

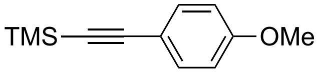

((p-Methoxyphenyl)ethynyl)trimethylsilane, **2r**

Yellow liquid (591 mg, 72%), ^{1}H NMR (400 MHz, $CDCl_3$) δ 7.40 (d, J = 8.6 Hz, 2H), 6.82 (d, J = 8.6 Hz, 2H), 3.80 (s, 3H), 0.24 (s, 9H), ^{13}C NMR (101 MHz, $CDCl_3$) δ 159.68, 133.44, 115.20, 113.76, 105.15, 92.39, 55.24, 0.05. In accordance with previously reported spectra.[12]

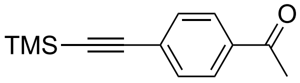

((p-Acetylphenyl)ethynyl)trimethylsilane, **2y**

Pale yellow liquid (646 mg, 75%), ^{1}H NMR (400 MHz, $CDCl_3$) δ 7.88 (d, J = 8.3 Hz, 2H), 7.53 (d, J = 8.3 Hz, 2H), 2.59 (s, 3H), 0.26 (s, 9H), ^{13}C NMR (101 MHz, $CDCl_3$) δ 197.25, 136.38, 132.04, 128.08, 127.94, 103.99, 98.08, 26.58, -0.19. In accordance with previously reported spectra.[17]

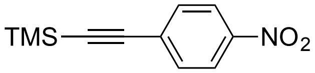

((p-Nitroxyphenyl)ethynyl)trimethylsilane, **3w**

Light yellow solid (500 mg, 57%), ^{1}H NMR (400 MHz, $CDCl_3$) δ 8.17 (d, J = 8.9 Hz, 2H), 7.60 (d, J = 8.9 Hz, 2H), 0.27 (s, 9H), ^{13}C NMR (101 MHz, $CDCl_3$) δ 147.10, 132.67, 129.95, 123.47, 102.66, 100.61, -0.31. In accordance with previously reported spectra.[12]

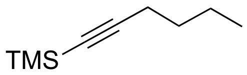

Procedure for 1-trimethylsilyl-1-hexyne preparation, 2s[8]

1-Hexyne (20.4 mmol) was dissolved in THF (60 mL). After cooling to –78 °C, n-BuLi (2.5M in hexanes, 8.3 mL, 20.8 mmol) was added dropwise to the solution. The mixture was stirred for 2 h at –78 °C. The trimethylsilylchloride (20.0 mmol) dissolved in THF (10 mL) was added dropwise. The mixture was stirred 3 h at 23 °C. A saturated solution of aqueous ammonium chloride (100 mL) was added and the mixture was extracted with dichloromethane (2 × 100 mL). The collected organic layer was washed with a mixture of 50% (V/V) brine (100 mL) and water (100 mL), then dried over $MgSO_4$, filtered and concentrated under reduced pressure. The residue was purified by flash chromatography on silica gel. Colorless liquid (1.14 g, 37%), ^{1}H NMR (400 MHz, $CDCl_3$) δ 2.19 (t, J = 7.1 Hz, 2H), 1.52 – 1.33 (m, 4H), 0.89 (t, J = 7.3 Hz, 3H), 0.12 (s, 9H), ^{13}C NMR (101 MHz, $CDCl_3$) δ 107.56, 84.11, 30.75, 21.90, 19.53, 13.58, 0.14. In accordance with previously reported spectra.[18]

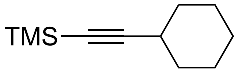

Procedure for (cyclohexylethynyl)trimethylsilane preparation, 2t[19]

To a solution of ethynylcyclohexane (1.66 mL, 12.8 mmol) in THF (54 mL, 0.24 M) under N_2 at –78 ºC was added n-BuLi (2.5 M solution in hexanes, 5.6 mL, 14.0 mmol). The mixture was stirred for 30 min and then the cooling bath had been removed. The mixture was stirred for extra 30 min. The mixture was stirred for 17 h after the addition of trimethylsilylchloride (1.77 mL, 14.0 mmol). The mixture was diluted with Et_2O (50 mL) and H_2O (50 mL) and the layers were separated. After the aqueous layer had been extracted again with Et_2O (3 × 50 mL), the combined extracts were dried with $MgSO_4$ and concentrated. The residue was purified by flash

chromatography on silica gel. Colorless liquid (1.43 g, 62%), ^{1}H NMR (400 MHz, $CDCl_3$) δ 2.45 – 2.29 (m, 1H), 1.89 – 1.62 (m, 4H), 1.53 – 1.39 (m, 3H), 1.36 – 1.19 (m, 3H), 0.14 (s, 9H), ^{13}C NMR (101 MHz, $CDCl_3$) δ 111.83, 83.66, 32.63, 30.00, 25.86, 24.81, 0.28. In accordance with previously reported spectra.[20]

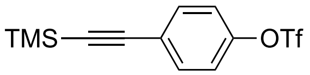

Procedure for 4-((trimethylsilyl)ethynyl)phenyl trifluoromethanesulfonate preparation, 2z[21]

A 20 mL vial was charged with 4-((trimethylsilyl)ethynyl)phenol[38] (500 mg, 2.62 mmol, 1.0 equiv.) and DCM (3 mL). the resulting solution was cooled to 0 ^{o}C, and pyridine (0.38g, 4.8 mmol, 2.0 equiv.) was added dropwise via syringe. A solution of trifilic anhydride (812 mg, 2.88 mmol, 1.1 equiv.) in DCM (1 mL) was added dropwise via syringe, and the vial was allowed to warm to 23 ^{o}C. After stirring for 1 h, the reaction mixture was diluted with Et_2O (20 mL), washed with 1 M HCl (20 mL), sat. $NaHCO_3$ (20 mL), H_2O (20 mL) and brine (20 mL). the organic layer was dried with $MgSO_4$ and concentrated in vacuo and purified via flash chromatography. Yellow liquid (543 mg, 64%), ^{1}H NMR (500 MHz, $CDCl_3$) δ 7.53 (d, J = 8.8 Hz, 2H), 7.21 (d, J = 8.8 Hz, 2H), 0.25 (s, 9H), ^{13}C NMR (101 MHz, $CDCl_3$) δ 149.04, 133.76, 123.85, 121.33, 118.71 (q, J = 322 Hz), 102.77, 96.62, -0.28. In accordance with previously reported spectra.[21]

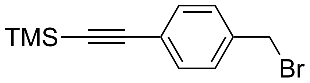

Procedure for ((4-(bromomethyl)phenyl)ethynyl)trimethylsilane preparation, 2aa[22]

To a solution of ((*p*-Tolyl)ethynyl)trimethylsilane, **2q** (900 mg, 4.78 mmol, 1.0 equiv.) in CCl_4 (7.5 mL), NBS (1.19 g, 6.70 mmol, 1.4 equiv.) and benzoyl peroxide (25 mg) were added. Then, reaction mixture was refluxed under nitrogen. After 3h, iced water (5 mL) was added to the reaction mixture, and the precipitate was removed by filtration. After the organic layer was separated, the solvent was evaporated under reduced pressure. The residue was then diluted with Et2O (10 mL) and the ethereal layer was washed with water and dried over $MgSO_4$. After filtration, the solvent was evaporated the resulting oliy product was purified by column chromatography. Yellow liquid (484 mg, 38%), ^{1}H NMR (500 MHz, $CDCl_3$) δ 7.43 (d, J = 8.1

Hz, 2H), 7.32 (d, J = 8.0 Hz, 2H), 4.46 (s, 2H), 0.25 (s, 9H), ^{13}C NMR (101 MHz, $CDCl_3$) δ 137.91, 132.20, 128.83, 123.15, 104.43, 95.14, 32.80, -0.10. In accordance with previously reported spectra.[22]

Preparation of gold catalyst and photoredox catalysts

Procedure for triphenylphosphinegold(I) chloride preparation[23]

Gold(III) chloride (5.11 mmol) was dissolved in ethanol (70 mL). 12N aqueous HCl solution was added dropwise with stirring until the solution became yellow and homogeneous. A solution of triphenylphosphine (7.24 mmol) in ethanol (100 mL) was added to the gold solution. The mixture immediately became colorless and a white precipitate appeared. The mixture was stirred for 2 min and the precipitate was collected by filtration, washed with diethyl ether (3 × 30 mL) and dried in vaccuo. The solid was dissolved in dichloromethane (20 mL) and was crystallized using hexane (120 mL) at –25 °C. Colorless solid (1.93 g, 75%), ^{1}H NMR (400 MHz, $CDCl_3$) δ 7.59 – 7.42 (m, 18H), ^{13}C NMR (101 MHz, $CDCl_3$) δ 134.19, 134.05, 131.98, 131.95, 129.27, 129.15.

Procedure for Tris(2,2'-bipyridine)ruthenium(II) hexafluorophosphate preparation[24]

Ruthenium(III) chloride (2.60 mmol) and 2,2'-bipyridine (16.0 mmol) were dissolved in ethanol (100 mL). The reaction mixture was heated to reflux for 12 h under nitrogen atmosphere. After cooling to 23 °C, potassium hexafluorophosphate (10 mmol) was added to the mixture and the precipitate was collected by filtration. The red solid was washed with water and then washed through the fritted funnel with acetone to remove excess ruthenium salts. The acetone eluent was diluted with diethyl ether to precipitate the ruthenium complex. The red precipitate was filtered and dried overnight in vaccuo. Red solid (1.82 g, 82%), ^{1}H NMR (300 MHz, DMSO-d_6) δ 8.84 (dd, J = 8.2, 1.4 Hz, 6H), 8.17 (ddd, J = 8.2, 7.2, 1.4 Hz, 6H), 7.73 (dd, J = 5.8, 1.4 Hz, 6H), 7.53 (ddd, J = 7.2, 5.8, 1.4 Hz, 6H), ^{13}C NMR (101 MHz, DMSO) δ 156.38, 151.05, 137.77, 127.74, 124.32.

Procedure for (4,4'-di-*tert*-butyl-2,2'-bipyridine)bis(2-(2-pyridinyl)phenyl)Iridium(III) hexa fluorophosphate preparation [25]

A mixture of 2-ethoxyethanol (30 mL) and water (10 mL) was placed in a flask containing $IrCl_3 \cdot 3H_2O$ (388 mg, 1.3 mmol) and 2-phenylpyridine (760 mg, 4.9 mmol). The mixture was heated under reflux for 24 h. After the mixture was cooled to 23 °C, the yellow precipitate was collected by filtration. The precipitate was washed with absolute ethanol (60 mL) and acetone (60 mL) and dissolved in dichoromethane (75 mL) and filtered. The filtrate was collected and solvent was removed in vaccuo to give the crude $[Ir(ppy)_2Cl]_2$ which was used for the next step without further purification. Green solid (415 mg, 60%)

A mixture of 4,4′-di-tert-butyl-2,2′-dipyridyl (118 mg, 0.44 mmol) and $[Ir(ppy)_2Cl]_2$ (214 mg, 0.2 mmol) in 1,2-ethanediol (10 mL) under nitrogen was heated to 150 °C and stirred for 15 h. After the mixture was cooled to 23 °C, water (150 mL) was added. A solution was extracted with diethyl ether (3 × 50 mL). The aqueous layer was heated to 70 °C and NH_4PF_6 (1.00 g) in water (10 mL) was added to the aqueous solution. After the mixture was cooled to 5 °C, the yellow precipitate was collected by filtration, dried in vaccuo and recrystallized by vapor diffusion with acetonitrile and diethyl ether. Yellow solid (293 mg, 73%), ^{1}H NMR (400 MHz, Acetone-d_6) δ 8.88 (d, J = 2.0 Hz, 2H), 8.23 (dd, J = 8.2, 1.3 Hz, 2H), 8.01 – 7.86 (m, 6H), 7.79 (dt, J = 5.8, 1.1 Hz, 2H), 7.70 (dd, J = 5.9, 2.0 Hz, 2H), 7.13 (ddd, J = 7.3, 5.8, 1.4 Hz, 2H), 7.03 (td, J = 7.5, 1.2 Hz, 2H), 6.91 (td, J = 7.4, 1.4 Hz, 2H), 6.34 (dd, J = 7.6, 1.2 Hz, 2H), 2.84 (d, J = 13.4 Hz, 2H), 1.41 (s, 18H).

General procedure for gold-catalyzed aryl-akynyl cross coupling

Aryldiazonium tetrafluoroborate (0.24 mmol, 1.2 equiv.), alkynyltrimethylsilane (0.2 mmol, 1 equiv.) and triphenylphosphinegold(I) chloride (10 mg, 0.02 mmol, 10 mol%) were placed in a vial equipped with a stir bar. To the vial kept at –78°C was added the solution of tris(2,2'-bipyridine)ruthenium(II) hexafluorophosphate (3.3 mg, 0.004 mmol, 2 mol%) in acetonitrile (2 mL). The vial was evacuated and refilled with nitrogen three times. The mixture was slowly warmed up to 23 °C and stirred for 3 h under a household lamp with a 23 W fluorescent light

bulb. After the completion of reaction, diethyl ether (20 mL) was added and filtered through a Celite plug. The filtrate was dried in vaccuo and purified by flash column chromatography to give the desired product.

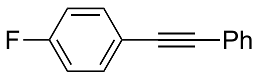

1-(*p*-Fluorophenyl)-2-phenylacetylene, **3a**

White solid (27.0 mg, 69%), ^{1}H NMR (500 MHz, $CDCl_3$) δ 7.54 – 7.50 (m, 4H), 7.37 – 7.33 (m, 3H), 7.07 – 7.03 (m, 2H), ^{13}C NMR (101 MHz, $CDCl_3$) δ 163.71, 161.23, 133.49, 133.41, 131.53, 128.35, 128.31, 123.06, 119.33, 115.73, 115.51, 89.01, 88.26, ^{19}F NMR (376 MHz, $CDCl_3$) δ -114.2, m/z HRMS (EI) found 196.0690 $C_{14}H_9F$ requires 196.0688. In accordance with previously reported spectra.[9]

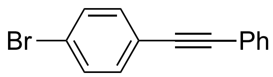

1-(*p*-Bromoophenyl)-2-phenylacetylene, **3b**

White solid (34.7 mg 68%), ^{1}H NMR (400 MHz, $CDCl_3$) δ 7.56 – 7.53 (m, 2H), 7.50 – 7.48 (m, 2H), 7.42 – 7.35 (m, 5H), ^{13}C NMR (101 MHz, $CDCl_3$) δ 132.98, 131.57, 131.55, 128.47, 128.36, 122.85, 122.43, 122.18, 90.47, 88.28, m/z HRMS (EI) found 255.9888 $C_{14}H_9Br$ requires 255.9888. In accordance with previously reported spectra.[26]

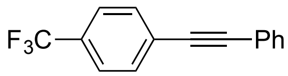

1-(*p*-Trifluoromethylphenyl)-2-phenylacetylene, **3c**

White solid (40.4 mg, 82%), ^{1}H NMR (400 MHz, $CDCl_3$) δ 7.65 – 7.56 (m, 6H), 7.39 – 7.38 (m, 3H), ^{13}C NMR (101 MHz, $CDCl_3$) δ 132.49, 131.78, 131.73, 131.48, 130.36, 130.04, 129.72, 129.39, 129.25, 128.81, 128.51, 128.43, 128.00, 127.11, 126.78, 125.31, 125.27, 125.23, 125.20, 124.62, 122.59, 122.55, 119.88, 91.74, 87.95, ^{19}F NMR (376 MHz, $CDCl_3$) δ -65.9, m/z HRMS (EI) found 246.0659 $C_{15}H_9F_3$ requires 246.0656. In accordance with previously reported spectra.[11]

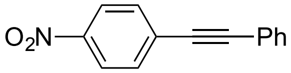

1-(*p*-Nitrophenyl)-2-phenylacetylene, **3d**

Yellow solid (31.7 mg, 71%), ^{1}H NMR (400 MHz, $CDCl_3$) δ 8.24 – 8.20 (m, 2H), 7.68 – 7.65 (m, 2H), 7.58 – 7.55 (m, 2H), 7.41 – 7.38 (m, 3H), ^{13}C NMR (101 MHz, $CDCl_3$) δ 146.91, 132.23, 131.80, 130.22, 129.25, 128.51, 123.60, 122.04, 94.67, 87.52, m/z HRMS (EI) found 223.0635 $C_{14}H_9NO_2$ requires 223.0633. In accordance with previously reported spectra.[9]

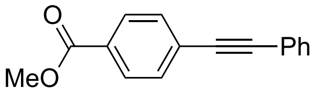

1-(*p*-Methoxycarbonylphenyl)-2-phenylacetylene, **3e**

White solid (33.5 mg, 71%), ^{1}H NMR (400 MHz, $CDCl_3$) δ 8.03 – 8.01 (m, 2H), 7.60 – 7.58 (m, 2H), 7.56 – 7.54 (m, 2H), 7.37 – 7.36 (m, 3H), 3.93 (s, 3H), ^{13}C NMR (101 MHz, $CDCl_3$) δ 166.51, 131.69, 131.46, 129.47, 129.40, 128.72, 128.39, 127.95, 122.64, 92.31, 88.58, 52.20, m/z HRMS (EI) found 236.0838 $C_{16}H_{12}O_2$ requires 236.0837. In accordance with previously reported spectra.[26]

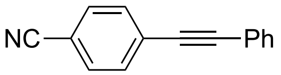

1-(*p*-Cyanophenyl)-2-phenylacetylene, **3f**

White solid (30.9 mg, 76%), ^{1}H NMR (400 MHz, $CDCl_3$) δ 7.65 – 7.60 (m, 4H), 7.56 – 7.54 (m, 2H), 7.39 – 7.37 (m, 3H), ^{13}C NMR (101 MHz, $CDCl_3$) δ 132.02, 131.99, 131.74, 129.08, 128.46, 128.20, 122.19, 118.47, 111.44, 93.75, 87.69, m/z HRMS (EI) found 203.0736 $C_{15}H_9N$ requires 203.0735. In accordance with previously reported spectra.[11]

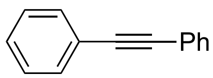

Diphenylacetylene, **3g**

White solid (20.5 mg, 58%), ^{1}H NMR (600 MHz, $CDCl_3$) δ 7.55 – 7.53 (m, 4H), 7.36 – 7.35 (m, 6H), ^{13}C NMR (101 MHz, $CDCl_3$) δ 131.58, 128.32, 128.23, 123.22, 89.34, m/z HRMS (EI) found 178.0780 $C_{14}H_{10}$ requires 178.0783. In accordance with previously reported spectra.[9]

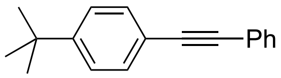

1-(*p*-*tert*-Butylphenyl)-2-phenylacetylene, **3h**

White solid (19.6 mg, 42%), ^{1}H NMR (400 MHz, $CDCl_3$) δ 7.56 – 7.51 (m, 2H), 7.48 (d, *J* = 8.3 Hz, 2H), 7.39 – 7.33 (m, 5H), 1.34 (s, 9H), ^{13}C NMR (101 MHz, $CDCl_3$) δ 151.48, 131.54, 131.30, 128.28, 128.04, 125.32, 123.47, 120.20, 89.51, 88.70, 34.76, 31.16, m/z HRMS (EI) found 234.1410 $C_{18}H_{18}$ requires 234.1409. In accordance with previously reported spectra.[27]

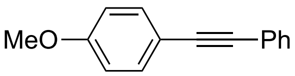

1-(*p*-Methoxyphenyl)-2-phenylacetylene, **3i**

Pale yellow solid (8.8 mg, 21%), ^{1}H NMR (400 MHz, $CDCl_3$) δ 7.51 – 7.46 (m, 4H), 7.35 – 7.20 (m, 3H), 6.89 – 6.87 (m, 2H), 3.83 (s, 3H), ^{13}C NMR (101 MHz, $CDCl_3$) δ ^{13}C NMR (101 MHz, $CDCl_3$) δ 159.59, 133.03, 132.49, 131.43, 128.43, 128.29, 127.91, 123.57, 115.36, 113.98, 89.34, 88.04, 55.30, m/z HRMS (EI) found 208.0889 $C_{15}H_{12}O$ requires 208.0888. In accordance with previously reported spectra.[28]

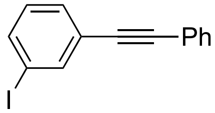

1-(*m*-Iodophenyl)-2-phenylacetylene, **3j**

White solid (29.0 mg, 48%), ^{1}H NMR (400 MHz, $CDCl_3$) δ 7.90 (t, *J* = 1.7 Hz, 1H), 7.67 (ddd, *J* = 8.0, 1.8, 1.1 Hz, 1H), 7.56 – 7.45 (m, 3H), 7.40 – 7.31 (m, 3H), 7.08 (t, *J* = 7.9 Hz, 1H), ^{13}C NMR (101 MHz, $CDCl_3$) δ 140.13, 137.20, 131.63, 130.68, 129.81, 128.58, 128.38, 125.36, 122.77, 93.67, 90.69, 87.61, m/z HRMS (EI) found 303.9752 $C_{14}H_9I$ requires 303.9749. In accordance with previously reported spectra.[29]

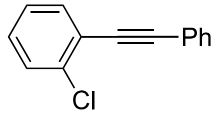

1-(*o*-Chlorophenyl)-2-phenylacetylene, **3k**

White solid (29.0 mg, 68%), ^{1}H NMR (400 MHz, $CDCl_3$) δ 7.62 – 7.55 (m, 3H), 7.46 – 7.42 (m, 1H), 7.39 – 7.34 (m, 3H), 7.30 – 7.22 (m, 2H), ^{13}C NMR (101 MHz, $CDCl_3$) δ ^{13}C NMR (101

MHz, $CDCl_3$) δ 135.92, 133.19, 131.72, 129.28, 129.21, 128.61, 128.35, 126.42, 123.22, 122.91, 94.52, 86.15, m/z HRMS (EI) found 212.0394 $C_{14}H_9Cl$ requires 212.0393. In accordance with previously reported spectra.[30]

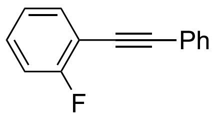

1-(*o*-Fluorophenyl)-2-phenylacetylene, **3l**

White solid (24.6 mg, 63%), ^{1}H NMR (400 MHz, $CDCl_3$) δ 7.58 – 7.51 (m, 3H), 7.37 – 7.29 (m, 4H), 7.15 – 7.09 (m, 2H), ^{13}C NMR (101 MHz, $CDCl_3$) δ 163.87, 161.37, 133.43, 131.69, 129.97, 129.89, 128.56, 128.34, 123.95, 123.91, 122.91, 115.61, 115.40, 112.01, 111.85, 94.41, 82.66, ^{19}F NMR (376 MHz, $CDCl_3$) δ -113.0, m/z HRMS (EI) found 196.0689 $C_{14}H_9F$ requires 196.0688. In accordance with previously reported spectra.[31]

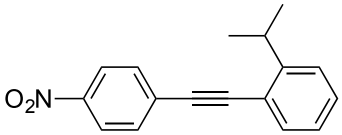

1-(*p*-Nitrophenyl)-2-(*o*-isopropylphenyl)acetylene, **3m**

Yellow solid (30.5 mg, 58%), ^{1}H NMR (400 MHz, $CDCl_3$) δ 8.23 (d, J = 8.9 Hz, 2H), 7.66 (d, J = 8.9 Hz, 2H), 7.56 – 7.48 (m, 1H), 7.40 – 7.31 (m, 2H), 7.24 – 7.16 (m, 1H), 3.51 (hept, J = 6.9 Hz, 1H), 1.33 (d, J = 6.9 Hz, 6H), ^{13}C NMR (101 MHz, $CDCl_3$) δ 150.91, 146.84, 132.61, 132.06, 130.54, 129.69, 125.73, 125.18, 123.64, 120.77, 93.74, 91.15, 31.80, 23.11, m/z HRMS (EI) found 265.1106 $C_{17}H_{15}NO_2$ requires 265.1103.

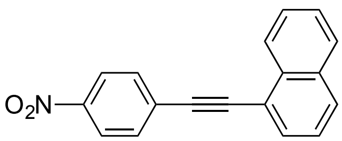

1-(*p*-Nitrophenyl)-2-(1-naphthyl)acetylene, **3n**

Yellow solid (23.4 mg, 43%), ^{1}H NMR (300 MHz, $CDCl_3$) δ 8.39 (d, J = 8.2 Hz, 1H), 8.28 (d, J = 8.5 Hz, 2H), 7.93 – 7.89 (m, 2H), 7.82 – 7.77 (m, 3H), 7.66 – 7.47 (m, 3H), ^{13}C NMR (101 MHz, $CDCl_3$) δ 147.00, 133.16, 133.10, 132.27, 131.12, 130.30, 129.88, 128.49, 127.16, 126.68,

125.82, 125.25, 123.71, 119.65, 92.94, 92.33. m/z HRMS (EI) found 273.0788 $C_{18}H_{11}NO_2$ requires 273.0790.

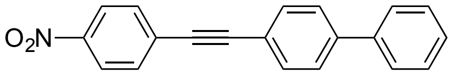

1-(*p*-Nitrophenyl)-2-(*p*-(1,1'-biphenyl)-yl)acetylene, **3o**

Yellow solid (35.4 mg, 59%), ^{1}H NMR (400 MHz, $CDCl_3$) δ 8.24 (d, *J* = 8.8 Hz, 2H), 7.69 (d, *J* = 8.8 Hz, 2H), 7.63 – 7.61 (m, 6H), 7.47 (t, *J* = 7.5 Hz, 2H), 7.40 – 7.38 (m, 1H), ^{13}C NMR (101 MHz, C_6D6) δ 146.92, 142.00, 140.02, 132.27, 132.22, 130.26, 128.91, 127.89, 127.17, 127.03, 123.64, 120.89, 94.69, 88.22, m/z HRMS (EI) found 299.0943 $C_{20}H_{13}NO_2$ requires 299.0946.

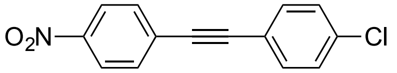

1-(*p*-Nitrophenyl)-2-(*p*-chlorophenyl)acetylene, **3p**

Pale yellow solid (39.8 mg, 77%), ^{1}H NMR (300 MHz, $CDCl_3$) δ 8.23 (d, *J* = 8.6 Hz, 2H), 7.66 (d, *J* = 8.6 Hz, 2H), 7.49 (d, *J* = 8.3 Hz, 2H), 7.37 (d, *J* = 8.3 Hz, 2H), ^{13}C NMR (101 MHz, $CDCl_3$) δ 147.11, 135.42, 133.02, 132.26, 129.84, 128.91, 123.65, 120.57, 93.40, 88.38, m/z HRMS (EI) found 257.0246 $C_{14}H_8NO_2Cl$ requires 257.0244.

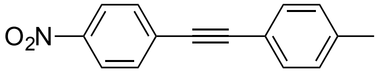

1-(*p*-Nitrophenyl)-2-*p*-tolylacetylene, **3q**

Yellow solid (36.4 mg, 77%), ^{1}H NMR (400 MHz, $CDCl_3$) δ 8.22 (d, *J* = 8.3 Hz, 2H), 7.65 (d, *J* = 8.3 Hz, 2H), 7.46 (d, *J* = 7.8 Hz, 2H), 7.20 (d, *J* = 7.7 Hz, 2H), 2.39 (s, 3H), ^{13}C NMR (101 MHz, $CDCl_3$) δ 146.83, 139.63, 132.13, 131.74, 130.51, 129.29, 123.60, 119.02, 95.09, 87.06, 21.59, m/z HRMS (EI) found 237.0792 $C_{15}H_{11}NO_2$ requires 237.0790. In accordance with previously reported spectra.[32]

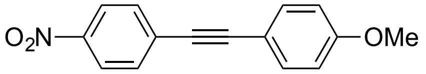

1-(*p*-Nitrophenyl)-2-(*p*-methoxyphenyl)acetylene, **3r**

Yellow (18.2 mg, 36%), ^{1}H NMR (300 MHz, $CDCl_3$) δ 8.21 (d, J = 8.5 Hz, 2H), 7.63 (d, J = 8.7 Hz, 2H), 7.50 (d, J = 8.8 Hz, 2H), 6.91 (d, J = 8.5 Hz, 2H), 3.85 (s, 3H), ^{13}C NMR (101 MHz, $CDCl_3$) δ 160.38, 146.64, 133.42, 131.96, 130.67, 123.60, 114.18, 114.08, 95.12, 86.61, 55.34, m/z HRMS (EI) found 253.0741 $C_{15}H_{11}NO_3$ requires 253.0739. In accordance with previously reported spectra.[33]

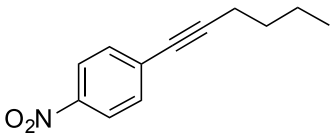

1-(p-Nitrophenyl)-1-hexyne, **3s**
Yellow oil (28.3 mg, 70%), ^{1}H NMR (300 MHz, $CDCl_3$) δ 8.15 (d, J = 8.6 Hz, 2H), 7.51 (d, J = 8.6 Hz, 2H), 2.45 (t, J = 7.0 Hz, 2H), 1.66 – 1.42 (m, 4H), 0.96 (t, J = 7.2 Hz, 3H), ^{13}C NMR (101 MHz, $CDCl_3$) δ 146.51, 132.19, 131.19, 123.43, 96.73, 79.25, 30.43, 22.00, 19.22, 13.57, m/z HRMS (EI) found 203.0947 $C_{12}H_{13}NO_2$ requires 203.0946. In accordance with previously reported spectra.[34]

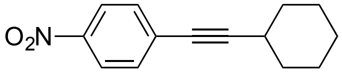

1-(p-Nitrophenyl)-2-cyclohexylacetylene, **3t**
Orange oil (36.4 mg, 79%), ^{1}H NMR (400 MHz, $CDCl_3$) δ 8.14 (d, J = 8.8 Hz, 2H), 7.51 (d, J = 8.8 Hz, 2H), 2.72 – 2.52 (m, 1H), 1.92 – 1.87 (m, 2H), 1.80 – 1.71 (m, 2H), 1.57 – 1.53 (m, 3H), 1.43 – 1.31 (m, 3H). ^{13}C NMR (101 MHz, $CDCl_3$) δ 146.46, 132.21, 131.25, 123.40, 100.63, 79.25, 32.31, 29.76, 25.76, 24.80, m/z HRMS (EI) found 229.1107 $C_{14}H_{15}NO_2$ requires 229.1103.

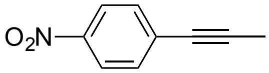

1-(p-Nitrophenyl)-1-propyne, **3u**
White solid (18.5 mg, 58%), ^{1}H NMR (400 MHz, $CDCl_3$) δ 8.15 (d, J = 8.9 Hz, 2H), 7.51 (d, J = 8.8 Hz, 2H), 2.10 (s, 3H), ^{13}C NMR (101 MHz, $CDCl_3$) δ 146.57, 132.17, 131.10, 123.47, 92.16, 78.43, 4.53, m/z HRMS (EI) found 161.0480 $C_9H_7NO_2$ requires 161.0477. In accordance with previously reported spectra.[35]

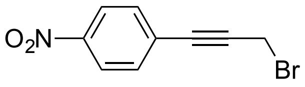

1-(*p*-Nitrophenyl)-3-bromo-1-propyne, **3v**

Pale yellow solid (23.5 mg, 49%), ^{1}H NMR (400 MHz, $CDCl_3$) δ 8.20 (d, *J* = 8.6 Hz, 2H), 7.59 (d, *J* = 8.6 Hz, 2H), 4.16 (s, 2H), ^{13}C NMR (101 MHz, $CDCl_3$) δ 147.43, 132.63, 128.92, 123.57, 89.29, 84.36, 14.02, m/z HRMS (EI) found 238.9581 $C_9H_6NO_2Br$ requires 238.9582. In accordance with previously reported spectra.[36]

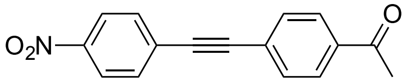

1-(*p*-Nitrophenyl)-2-(*p*-acetylphenyl)acetylene, **3y**

Pale yellow solid (35 mg, 66%), ^{1}H NMR (400 MHz, $CDCl_3$) δ 8.24 (d, *J* = 8.7 Hz, 2H), 7.98 (d, *J* = 8.3 Hz, 2H), 7.70 (d, *J* = 8.7 Hz, 2H), 7.65 (d, *J* = 8.3 Hz, 2H), 2.63 (s, 3H), ^{13}C NMR (101 MHz, $CDCl_3$) δ 197.13, 147.25, 136.91, 132.41, 131.93, 129.46, 128.32, 126.76, 123.65, 93.41, 90.29, 26.62, m/z HRMS (EI) found 265.0739 $C_{16}H_{11}NO_3$ requires 265.0739.

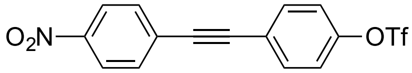

4-((4-nitrophenyl)ethynyl)phenyl trifluoromethanesulfonate, **3z**

Pale Yellow solid (42 mg, 57%), ^{1}H NMR (400 MHz, $CDCl_3$) δ 8.24 (d, *J* = 8.9 Hz, 2H), 7.68 (d, J = 8.9 Hz, 2H), 7.64 (d, J = 8.8 Hz, 2H), 7.31 (d, J = 8.8 Hz, 2H), ^{13}C NMR (101 MHz, $CDCl_3$) δ 149.51, 147.31, 133.70, 132.40, 129.34, 123.68, 122.77, 121.74, 118.66 (q, *J* = 322 Hz), 92.19, 89.10, m/z HRMS (EI) found 371.0075 $C_{15}H_8NO_5SF_3$ requires 371.0075.

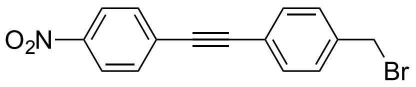

1-(*p*-Nitrophenyl)-2-(*p*-(bromomethyl)phenyl)acetylene, **3aa**

Yellow solid (34 mg, 54%), ^{1}H NMR (400 MHz, $CDCl_3$) δ 8.23 (d, *J* = 8.9 Hz, 2H), 7.67 (d, *J* = 8.9 Hz, 2H), 7.53 (d, *J* = 8.3 Hz, 2H), 7.41 (d, *J* = 8.2 Hz, 2H), 4.50 (s, 2H), ^{13}C NMR (101 MHz, $CDCl_3$) δ 147.04, 138.87, 132.29, 132.19, 129.96, 129.21, 123.63, 122.17, 94.03, 88.28, 32.65, m/z HRMS (EI) found 314.9895 $C_{15}H_{10}NO_2{}^{79}Br$ requires 314.9895.

Preparation of *p*-nitrophenylacetylene, **3x**[37]

To a solution of ((*p*-nitrophenyl)ethynyl)trimethylsilane, **3w** (0.5 mmol) in diethyl ether (3 mL) was added methanol (3 mL) and aqueous sodium hydroxide solution (1.5 mL, 10 wt%). The mixture was stirred for 10 min at 23 °C and neutralized with 1 N HCl solution. The organic layer was separated, and the aqueous layer was extracted with diethyl ether (3 × 3 mL). The combined organic layers were washed with brine (10 mL) and water (10 mL), dried over $MgSO_4$, and concentrated. The residue was purified by flash chromatography on silica gel. Yellow Solid (68.1 mg, 93%), ^{1}H NMR (500 MHz, $CDCl_3$) δ 8.20 (d, *J* = 8.6 Hz, 2H), 7.64 (d, *J* = 8.6 Hz, 2H), 3.36 (s, 1H), ^{13}C NMR (101 MHz, $CDCl_3$) δ 147.49, 132.93, 128.87, 123.52, 82.30, 81.57. In accordance with previously reported spectra.[12]

Preparation of 1-phenyl-3-*p*-fluorophenyl-1-propyne, **5**

p-Fluorobenzenediazonium tetrafluoroborate, **1a** (42.0 mg, 0.20 mmol, 1.0 eq.), 1-phenyl-1-trimethylsilylprop-1,2-diene, **4**[38] (38 mg, 0.20 mmol, 1.0 eq.) and triphenylphosphinegold(I) chloride (4.9 mg, 0.01 mmol, 5 mol%) were placed in a vial equipped with a stir bar. To the vial kept at –78°C was added the solution of tris(2,2'-bipyridine)ruthenium(II) hexafluorophosphate (1.7 mg, 0.002 mmol, 1 mol%) in acetonitrile (2 mL). The vial was evacuated and refilled with nitrogen three times. The mixture was slowly warmed up to 23 °C and stirred for 6 h under a household lamp with a 23 W fluorescent light bulb. After the completion of reaction, diethyl ether (20 mL) was added and filtered through a Celite plug. The filtrate was dried in vaccuo and purified by flash column chromatography to give the desired product. Pale orange solid (17.0 mg, 41%), ^{1}H NMR (400 MHz, $CDCl_3$) δ 7.44 (dd, J = 6.6, 2.9 Hz, 2H), 7.37 (dd, J = 8.1, 5.9 Hz, 2H), 7.33 – 7.28 (m, 3H), 7.03 (t, J = 8.7 Hz, 2H), 3.80 (s, 2H), ^{13}C NMR (101 MHz, $CDCl_3$) δ 161.73 (d, J = 244.6 Hz), 132.38 (d, J = 2.9 Hz), 131.61, 129.37 (d, J = 8.0 Hz), 128.25, 127.91, 123.50, 115.30 (d, J = 21.5 Hz), 87.25, 82.82, 25.00, ^{19}F NMR (376 MHz, $CDCl_3$) δ -119.7, m/z HRMS (EI) found 209.0768 $C_{15}H_{10}F$ requires 209.0767.

Preparation of 1-phenyl-1-*p*-fluorophenylprop-1,2-diene, **6**[39]

To a solution of 1-phenyl-1-propyne (116 mg, 1.00 mmol) in THF (7.7 mL) at –78 °C under N_2 was added *n*-BuLi (2.5 M in hexanes, 0.46 mL, 1.14 mmol). After stirring at –78 °C for 1 h, a solution of dry $ZnBr_2$ (450 mg, 2.00 mmol) in THF (10.0 mL) was added. After stirring 10 min, the reaction mixture was slowly warmed to 23 °C. $Pd(PPh_3)_4$ (39 mg, 5 mol %) and *p*-fluoroiodobenzene (77 μL, 0.67 mmol) were subsequently added at 23 °C with stirring. After stirring 1 h, the mixture was quenched with saturated NH_4Cl, extracted with diethyl ether, dried over $MgSO_4$ and concentrated. The residue was purified by flash column chromatography to give the desired product. White solid (132 mg, 93%), ^{1}H NMR (400 MHz, $CDCl_3$) δ 7.41 – 7.27 (m, 7H), 7.04 (t, J = 8.7 Hz, 2H), 5.27 (s, 2H), ^{13}C NMR (101 MHz, CDCl3) δ 209.68, 162.06 (d, J = 246.4 Hz), 136.08, 132.15 (d, J = 3.3 Hz), 129.98 (d, J = 8.0 Hz), 128.45, 128.26, 127.32, 115.29 (d, J = 21.5 Hz), 108.29, 78.20, ^{19}F NMR (376 MHz, $CDCl_3$) δ -118.4, m/z HRMS (EI) found 209.0769 $C_{15}H_{10}F$ requires 209.0767.

Preparation of 1-*p*-fluorophenyl-3-phenyl-1-propyne, **7**[38]

Preparation of LDA solution: To a solution of diisopropylamine (0.30 mL, 2 mmol) in THF (1 mL) at -78 ºC and under N_2 was added *n*-BuLi (2.5 M in hexanes, 0.80 mL, 2 mmol) and the mixture was stirred for 30 min at this temperature.

To a solution of 1-phenyl-1-propyne (116 mg, 1.00 mmol) in THF (7.7 mL) at –78 °C under N_2 was added the prepared LDA solution. The reaction mixture was slowly warmed to 23 °C and stirred for 1 h. To the mixture, a solution of dry ZnBr2 (450 mg, 2 mmol) in THF (10 mL) was added. After stirring at 23 °C for 25 min, $Pd(PPh_3)_4$ (39 mg, 5 mol %) and *p*-fluoroiodobenzene (77 μL, 0.67 mmol) were added. After stirring 1 h, the mixture was quenched with saturated NH_4Cl, extracted with diethyl ether, dried over $MgSO_4$ and concentrated. The residue was purified by flash column chromatography to give the desired product. Orange solid (141 mg, 99%), ^{1}H NMR (400 MHz, $CDCl_3$) 7.48 – 7.39 (m, 4H), 7.35 (t, J = 7.4 Hz, 2H), 7.30 – 7.22 (m, 1H), 7.00 (t, J = 8.7 Hz, 2H), 3.82 (s, 2H), ^{13}C NMR (101 MHz, $CDCl_3$) δ 162.15 (d, J = 248.6 Hz), 136.57, 133.38 (d, J = 8.2 Hz), 128.51, 127.88, 126.63, 119.67 (d, J = 3.4 Hz), 115.39

(d, J = 22.0 Hz), 87.16, 81.52, 25.60, ^{19}F NMR (376 MHz, $CDCl_3$) δ -115.0, m/z HRMS (EI) found 209.0768 $C_{15}H_{10}F$ requires 209.0767.

References

1. H. M. Nelson, S. H. Reisberg, H. P. Shunatona, J. S. Patel and F. D. Toste, *Angew. Chem. Int. Ed.*, 2014, **53**, 5600.
2. O. Blank, D. Ullrich, M. Kirschstein and M. R. Heinrich, *J. Org. Chem.*, 2007, **72**, 9609.
3. Y. Piao, B. Meany, L. R. Powell, N. Valley, H. Kwon, G. C. Schatz and Y. Wang, *Nat. Chem.*, 2013, **5**, 840.
4. S. M. Dirk, S. Pylypenko, S. W. Howell, J. E. Fulghum and D. R. Wheeler, *Langmuir*, 2005, **21**, 10899.
5. A. Prechter and M. R. Heinrich, *Synthesis,* 2011, **10**, 1515.
6. K. G. Rutherford and W. Redmond, *Org. Synth.*, 1963, **43**, 12.
7. Z. Y. Tang, Y. Zhang, T. Wang and W. Wang, *Synlett.*, 2010, **5**, 804.
8. C. C. Chen and J. Waser, *Org. Lett.*, 2015, **17**, 736.
9. M. Carril, A. Correa, and C. Bolm, *Angew. Chem. Int. Ed.*, 2008, **47**, 4862.
10. W. Uhl, D. Kovert, S. Zemke and A. Hepp, *Organometallics*, 2011, **30**, 4736.
11. T. Truong and O. Daugulis, *Org. Lett.*, 2011, **13**, 4172.
12. H. Ueda, M. Yamaguchi, H. Kameya, K. Sugimoto and H. Tokuyama, *Org. Lett.*, 2014, **16**, 4948.
13. M. Tobisu, H. Nakai and N. Chatani, *J. Org. Chem.*, 2009, **74**, 5471.
14. M. A. Pena, J. P. Sestelo, L. A. Sarandeses, *Synthesis*, 2005, **3**, 485.
15. M. Cai, J. Sha and Q. Xu, *Tetrahedron*, 2007, **63**, 4642.
16. A. M. Rawashdeh, I. A. Elder, J. Yang, A. Dass, C. Sotiriou-Leventis and N. Leventis, *Chem. Mater.*, 2004, **16**, 1493.
17. G. Fabrizi, A. Goggiamani, A. Sferrazza, and S. Cacchi, *Angew. Chem. Int. Ed.*, 2010, **49**, 4067.
18. O. Gager, J. Buendia and G. Cahiez, *Angew. Chem. Int. Ed.*, 2010, **49**, 1278.
19. M. P. Huestis, L. Chan, D. R. Stuart and K. Fagnou, *Angew. Chem. Int. Ed.*, 2011, **50**, 1338.
20. H. Zhang, X. Fu, J. Chen, E. Wang, Y. Liu and Y. Li, *J. Org. Chem.*, 2009, **74**, 9351.

21. S. N. Greszler, H. A. Reichard and G. C. Micalizio, *J. Am. Chem. Soc.*, 2012, **134**, 2766.
22. H. Kawamura, Y. Takeyama, M. Yamamoto, H. Kurihara, K. Morino and E. Yashima, *Chirality*, 2011, **23**, E35.
23. P. Braunstein, H. Lehner and D. Matt, *Inorg. Synth.*, 1990, **27**, 218.
24. M. A. Ischay, Z. Lu and T. P. Yoon, *J. Am. Chem. Soc.*, 2010, **132**, 8572.
25. (a) S. Sprouse, K. A. King, P. J. Spellane and R. J. Watts, *J. Am. Chem. Soc.*, 1984, **106**, 6647. (b) J. Ru, L. Guan, X. Tang, W. Dou, X. Yao, W. Chen, Y. Liu, G. Zhang, W. Liu, Y. Meng and C. Wang, *Inorg. Chem.*, 2014, **53**, 11498. (c) J. D. Slinker, A. A. Gorodetsky, M. S. Lowry, J. Wang, S. Parker, R. Rohl, S. Bernhard and G. G. Malliaras, *J. Am. Chem. Soc.*, 2004, **126**, 2763.
26. G. Fabrizi, A. Goggiamani, A. Sferrazza and S. Cacchi, *Angew. Chem. Int. Ed.*, 2010, **49**, 4067.
27. J. Moon, M. Jeong, H. Nam, J. Ju, J. H. Moon, H. M. Jung and S. Lee, *Org. Lett.*, 2008, **10**, 945.
28. S. B. Park and H. Alper, *Chem. Commun.*, 2004, 1306.
29. A. Orita, H. Taniguchi and J. Otera, *Chem. Asian J.*, 2006, **1**, 430.
30. Z. Novak, P. Nemes and A. Kotschy, *Org. Lett.*, 2004, **6**, 4917.
31. C. Xu, M. Xu, Y. Jia and C. Li, *Org. Lett.*, 2011, **13**, 1556.
32. A. R. Gholap, K. Venkatesan, R. Pasricha, T. Daniel, R. J. Lahoti and K. V. Srinivasan, *J. Org. Chem.*, 2005, **70**, 4869.
33. M. L. N. Rao, D. N. Jadhav and P. Dasgupta, *Org. Lett.*, 2010, **12**, 2048.
34. B. Liang, M. Dai, J. Chen and Z. Yang, *J. Org. Chem.*, 2005, **70**, 391.
35. U. Weerasooriya and J. C. Gilbert, *J. Org. Chem.*, 1982, **47**, 1837.
36. M. Maji, D. Mallick, S. Mondal, A. Anoop, S. S. Bag, A. Basak and E. D. Jemmis, *Org. Lett.*, 2011, **13**, 888.
37. Y. Feng, C. Xie, W. Qiao and H. Xu, *Org. Lett.*, 2013, **15**, 936.
38. D. R. Williams and A. A. Shah, *Chem. Commun*., 2010, **46**, 4297-4299.
39. S. Ma, Q. He and X. Zhang, *J. Org. Chem.*, 2005, **70**, 3336-3338.

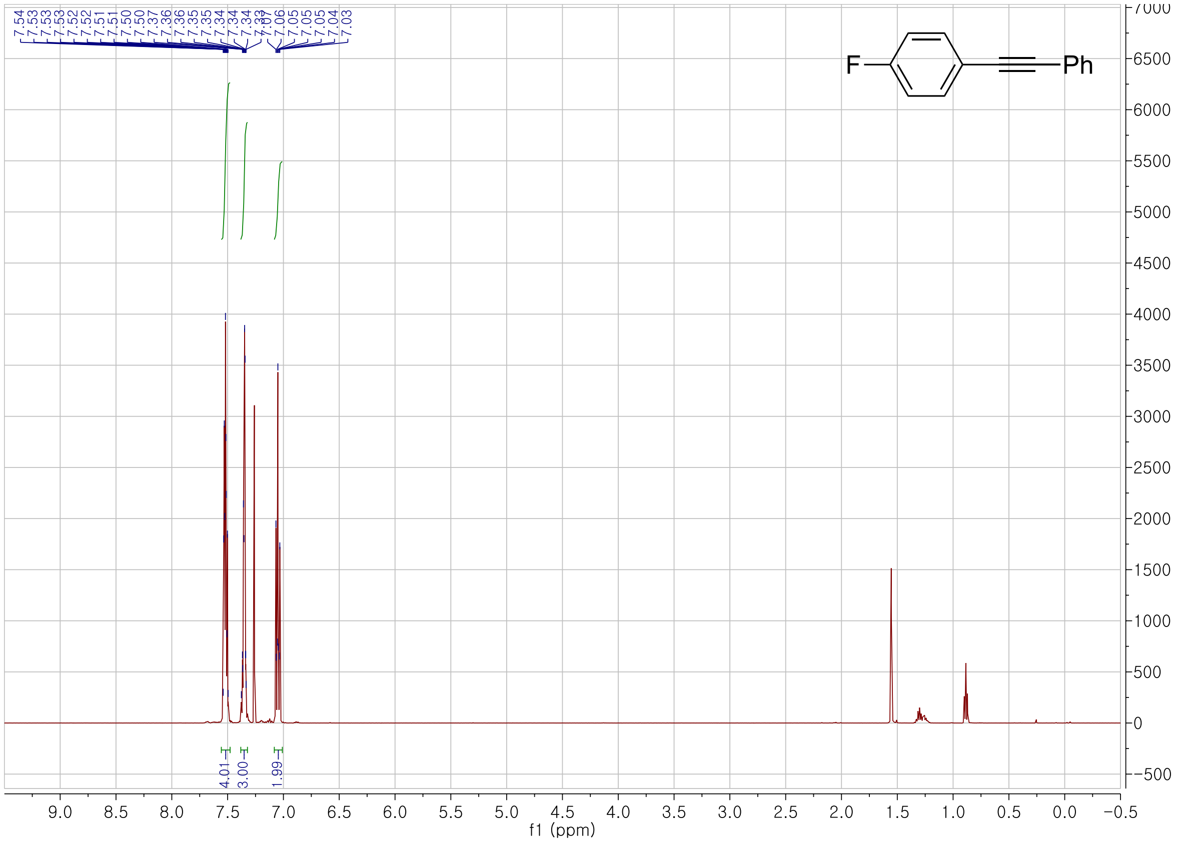
7.54
7.53
7.53
7.52
7.52
7.51
7.51
7.50
7.50
7.37
7.36
7.36
7.35
7.35
7.34
7.34
7.33
7.07
7.06
7.05
7.05
7.04
7.03
F
Ph
4.01
3.00
1.99
f1 (ppm)

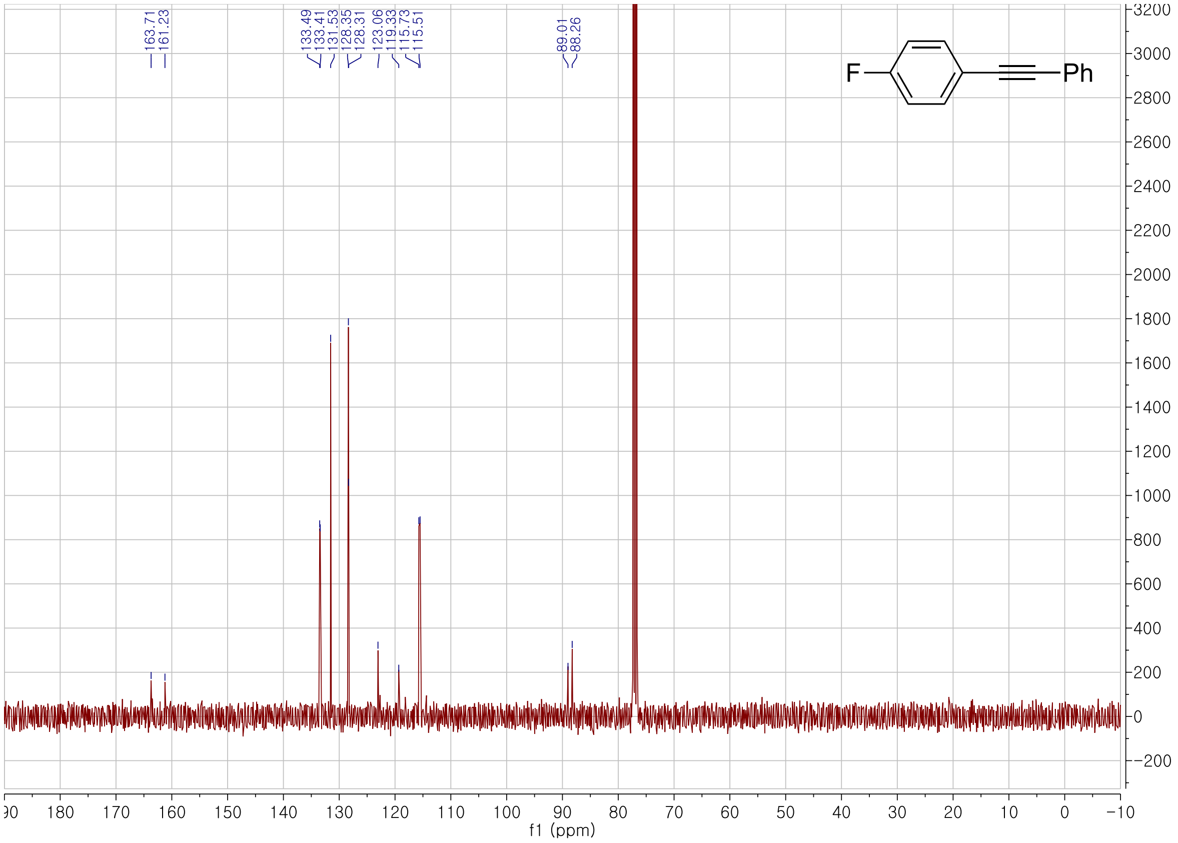
163.71
161.23
133.49
133.41
131.53
128.35
128.31
123.06
119.33
115.73
115.51
89.01
88.26
F
Ph
f1 (ppm)

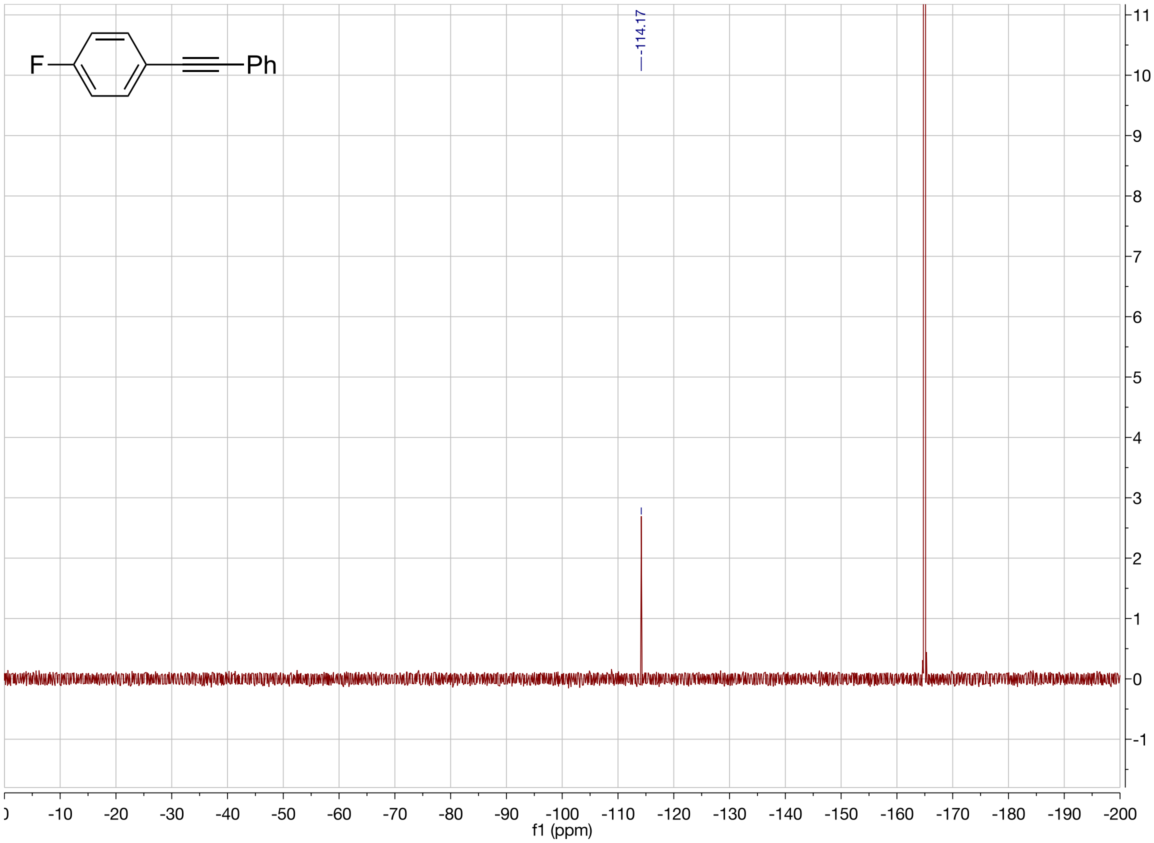
F
Ph
-114.17
f1 (ppm)

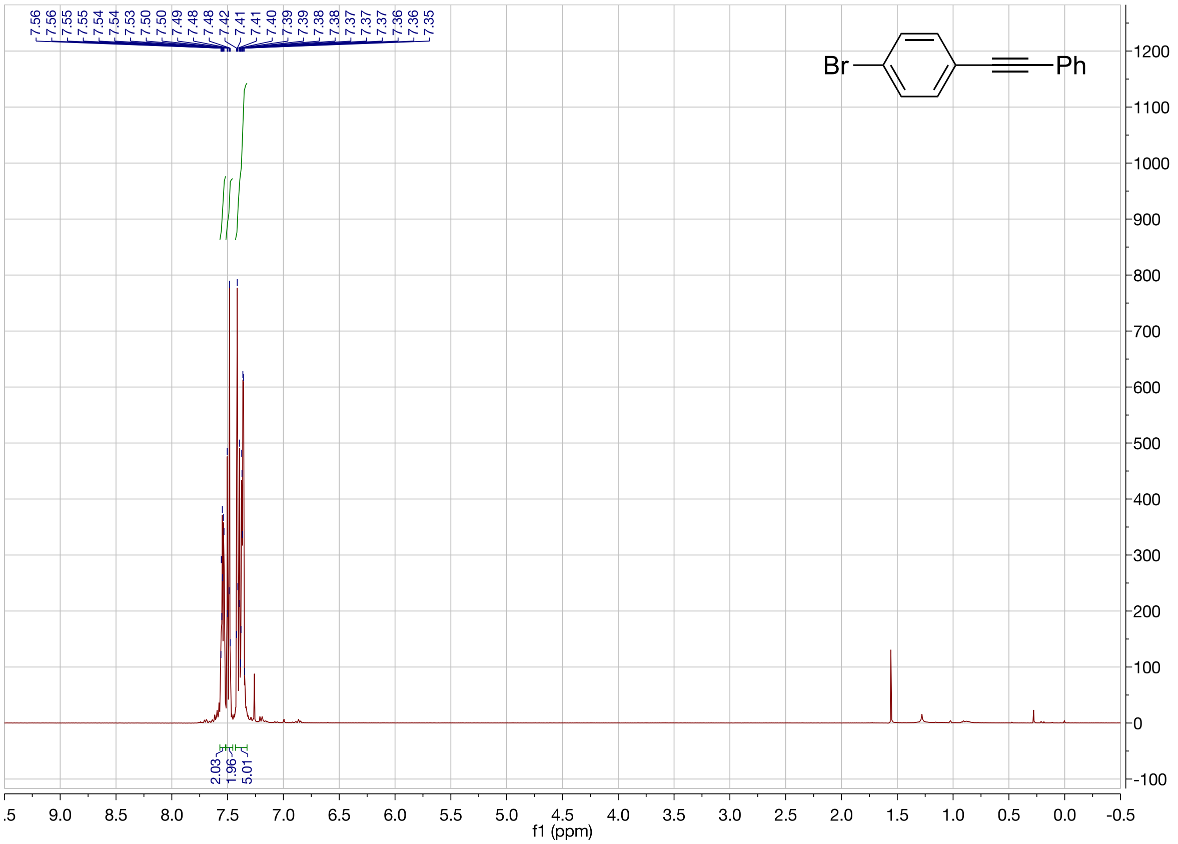

7.56 7.56 7.55 7.55 7.54 7.53 7.50 7.50 7.49 7.48 7.48 7.42 7.41 7.41 7.40 7.39 7.39 7.38 7.37 7.37 7.37 7.36 7.36 7.35
Br
Ph
2.03
1.96
5.01
f1 (ppm)

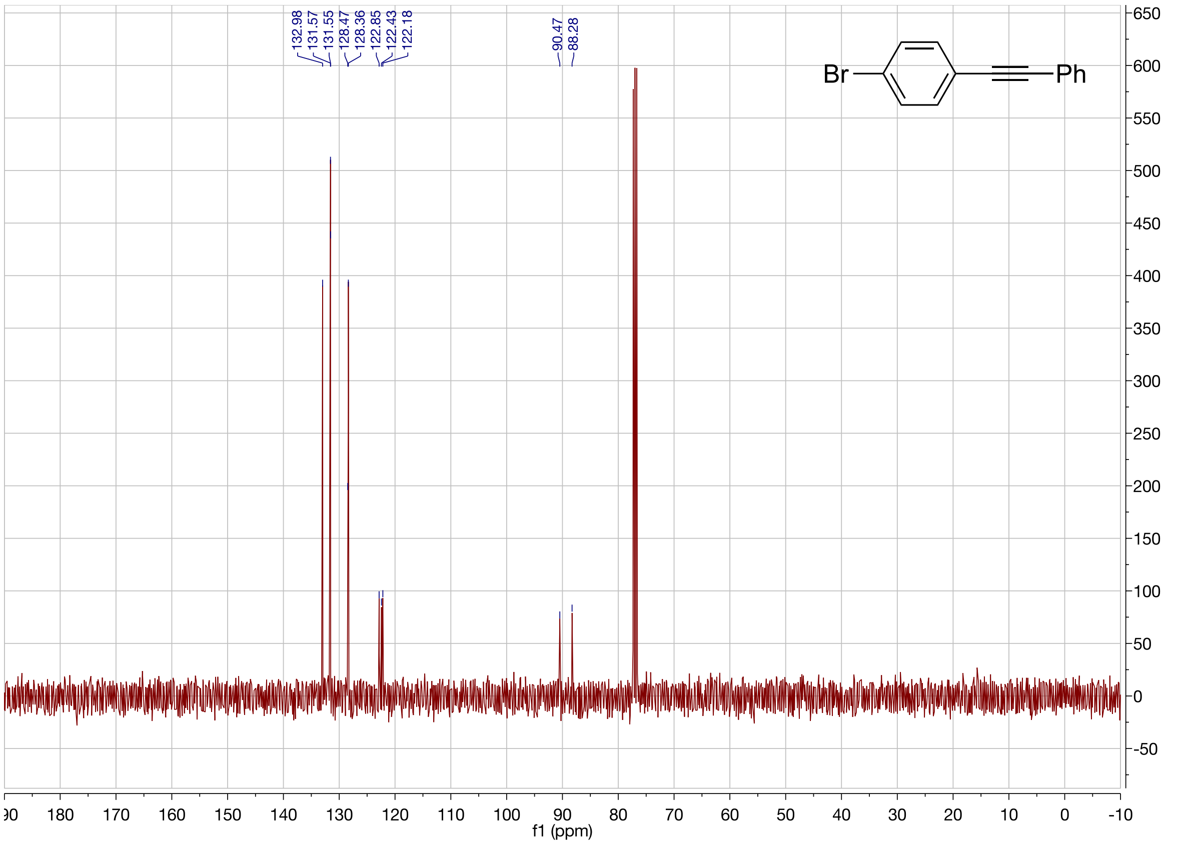

132.98 131.57 131.55 128.47 128.36 122.85 122.43 122.18
90.47 88.28
Br
Ph
f1 (ppm)

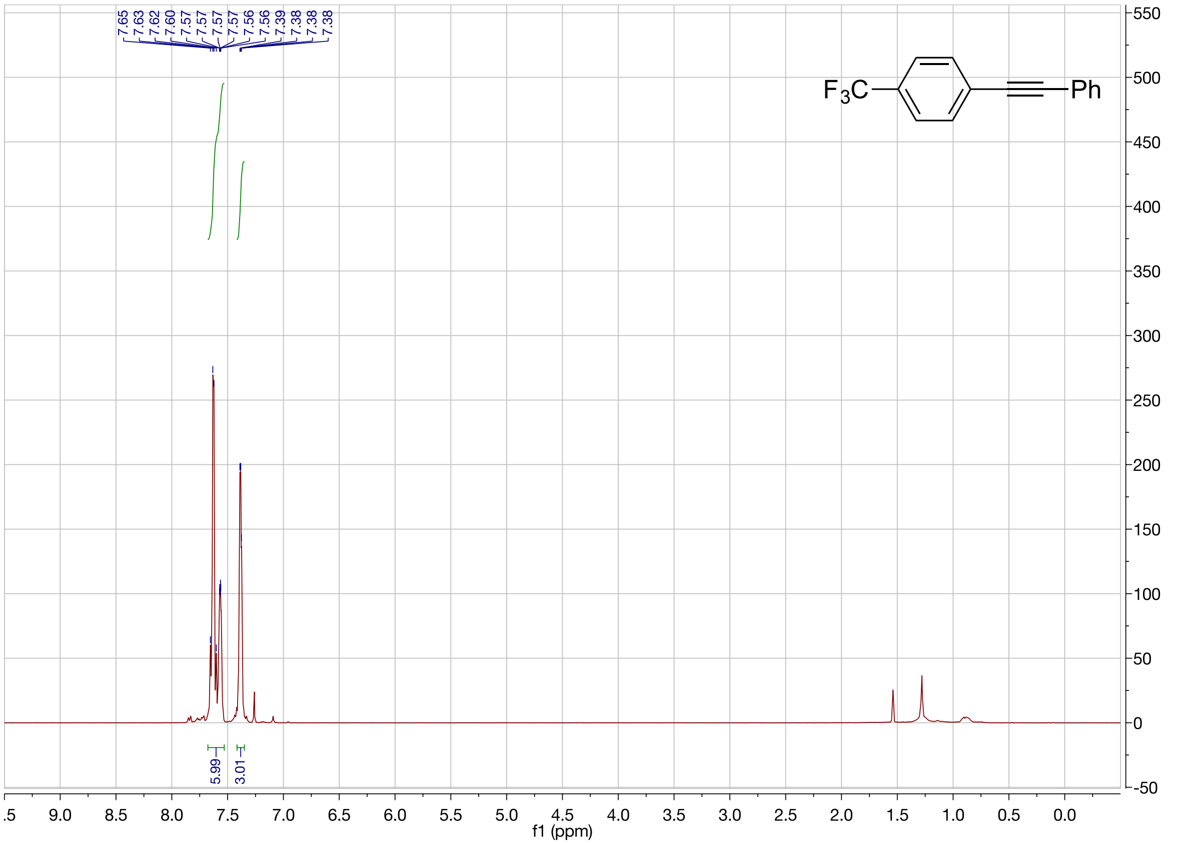

7.65
7.63
7.62
7.60
7.57
7.57
7.57
7.57
7.56
7.56
7.39
7.38
7.38
7.38
F3C
Ph
5.99
3.01
f1 (ppm)

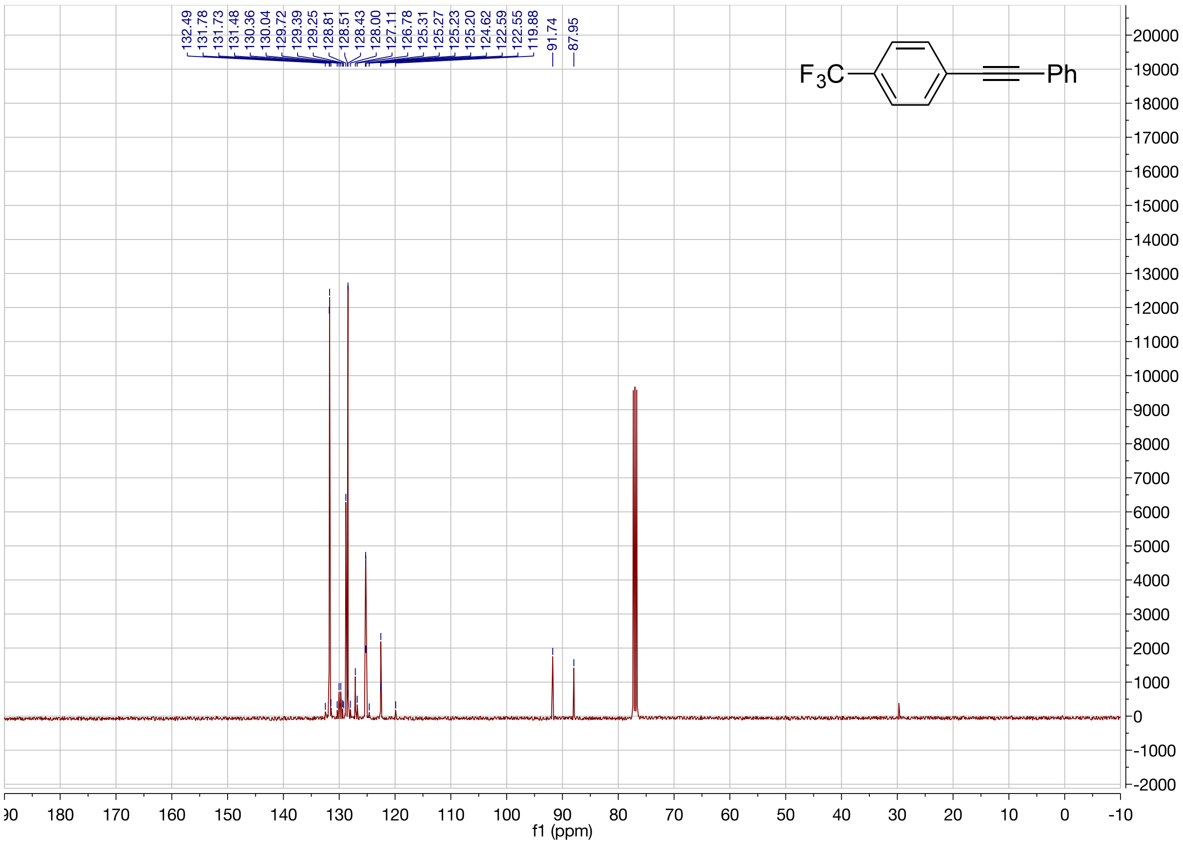

132.49
131.78
131.73
131.48
130.36
130.04
129.72
129.39
129.25
128.81
128.51
128.43
128.00
127.11
126.78
125.31
125.27
125.23
125.20
124.62
122.59
122.55
119.88
91.74
87.95
F3C
Ph
f1 (ppm)

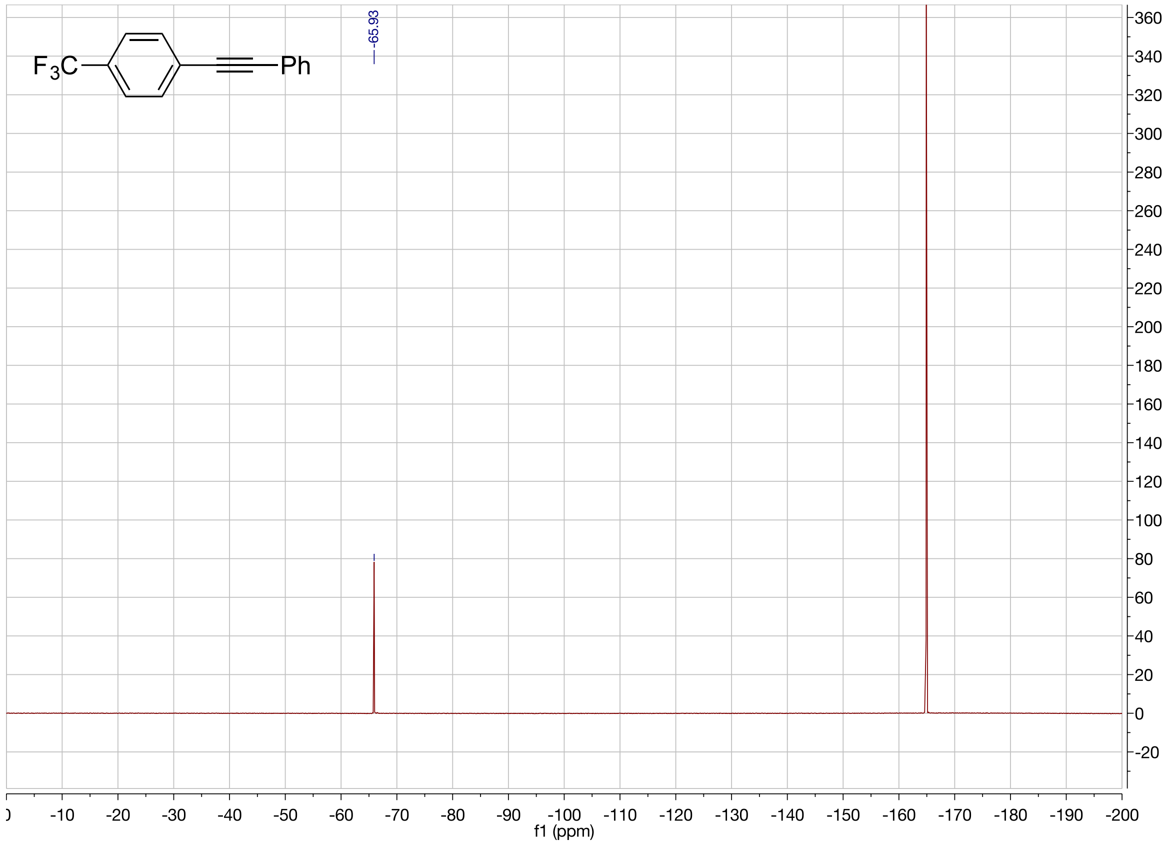

F3C
Ph
-65.93
f1 (ppm)

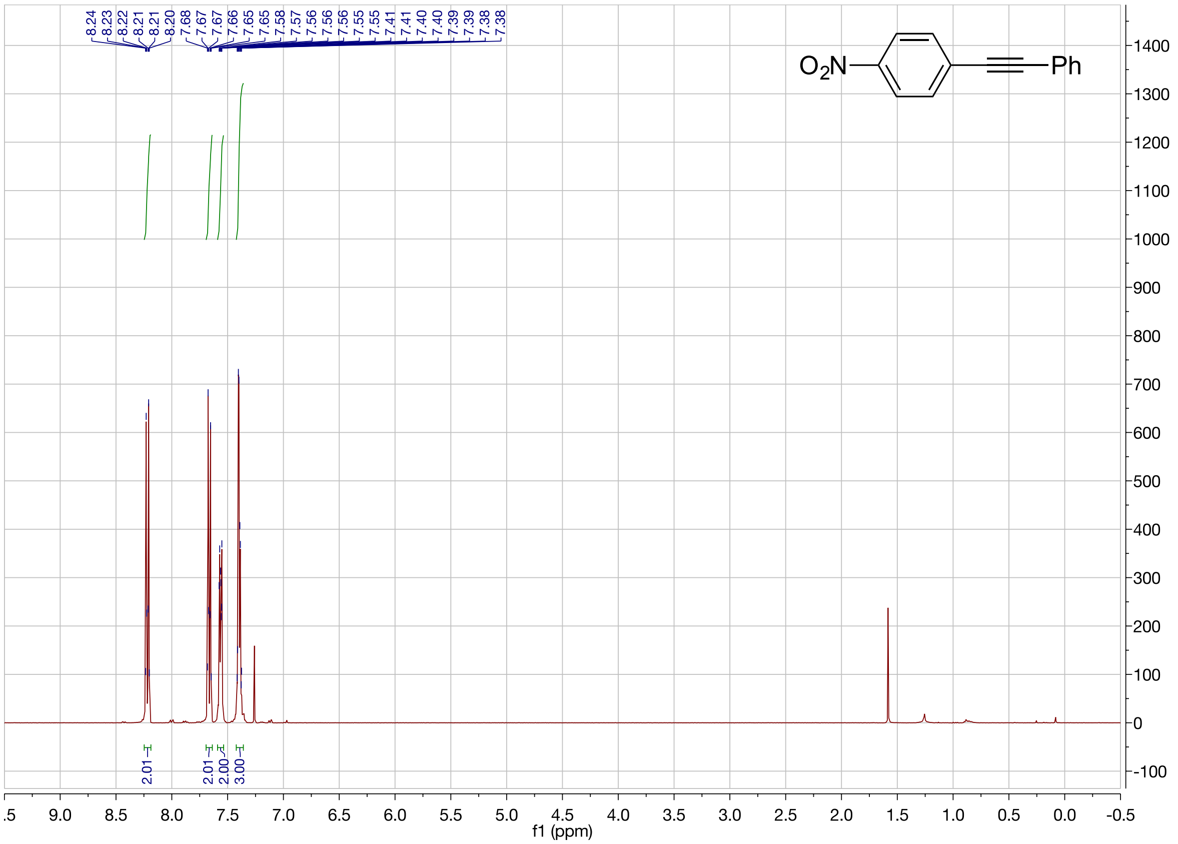

8.24
8.23
8.22
8.21
8.21
8.20
7.68
7.67
7.67
7.66
7.65
7.65
7.58
7.57
7.56
7.56
7.56
7.55
7.55
7.41
7.41
7.40
7.40
7.39
7.39
7.38
7.38
O_2N
Ph
2.01
2.01
2.00
3.00
f1 (ppm)

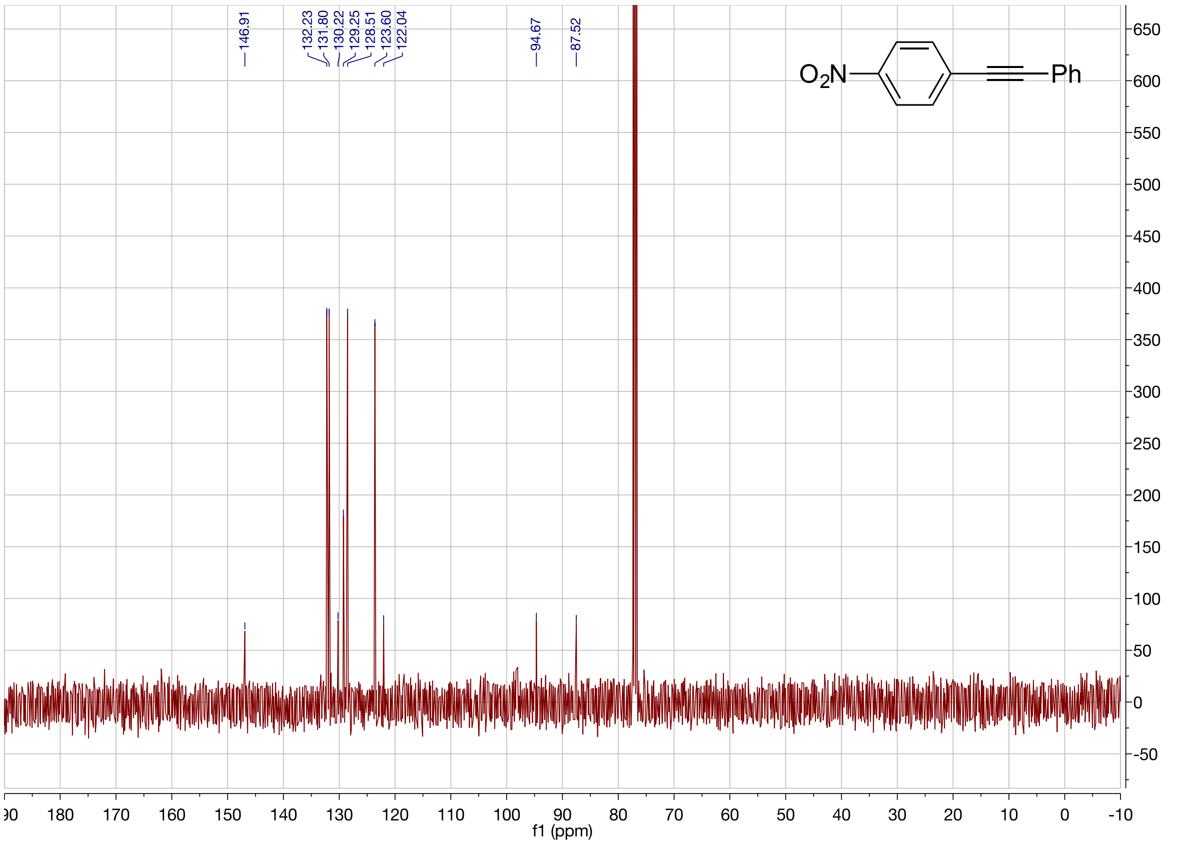

146.91
132.23
131.80
130.22
129.25
128.51
123.60
122.04
94.67
87.52
O_2N
Ph
f1 (ppm)

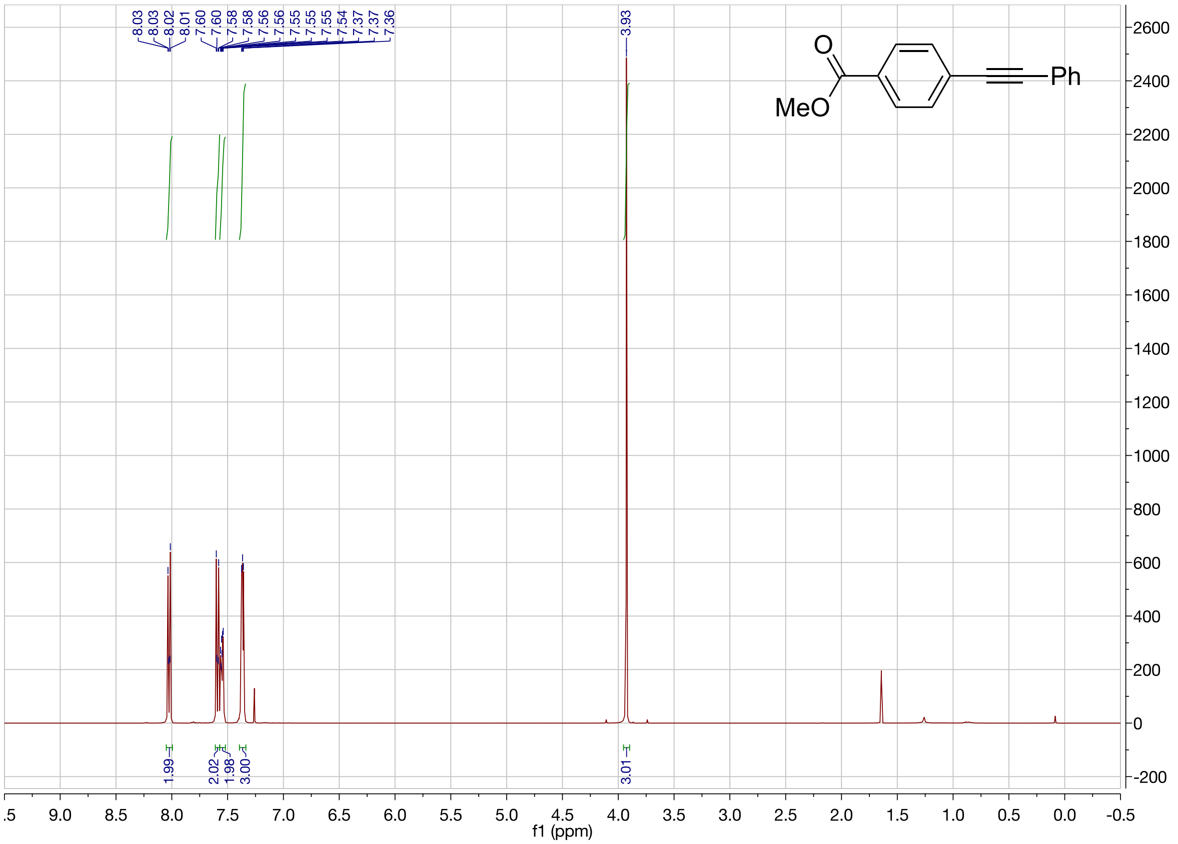
8.03
8.03
8.02
8.01
7.60
7.60
7.58
7.58
7.56
7.56
7.55
7.55
7.55
7.54
7.37
7.37
7.36
3.93
O
MeO
Ph
1.99
2.02
1.98
3.00
3.01
f1 (ppm)

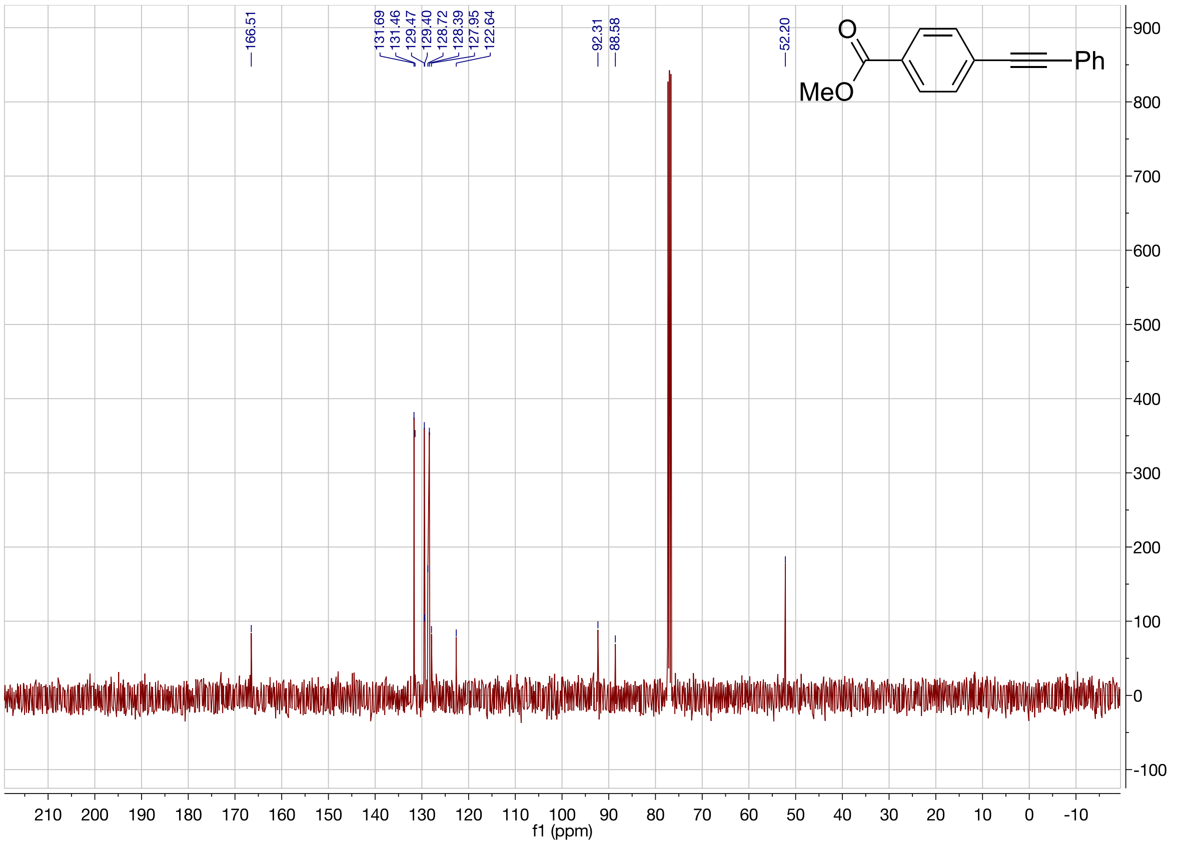
166.51
131.69
131.46
129.47
129.40
128.72
128.39
127.95
122.64
92.31
88.58
52.20
O
MeO
Ph
f1 (ppm)

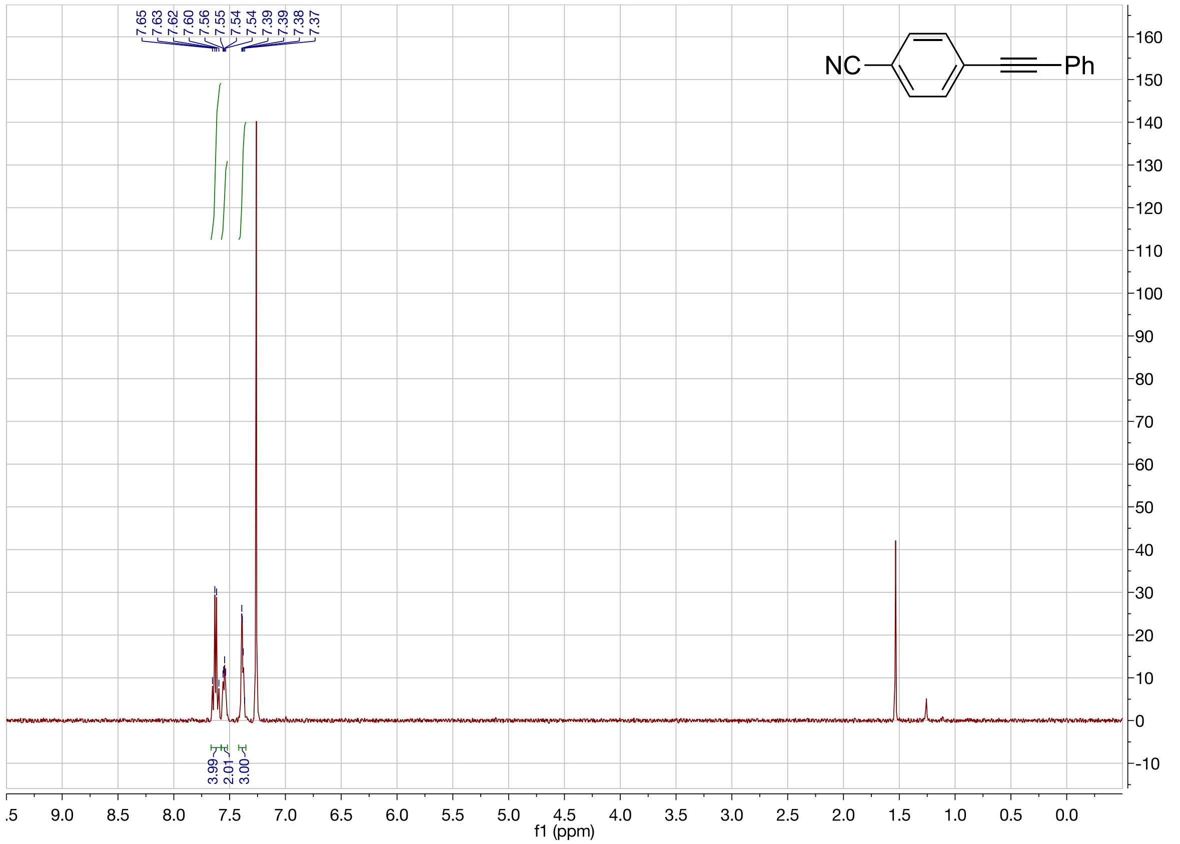
7.65
7.63
7.62
7.60
7.56
7.55
7.54
7.54
7.39
7.39
7.38
7.37
NC
Ph
3.99
2.01
3.00
f1 (ppm)

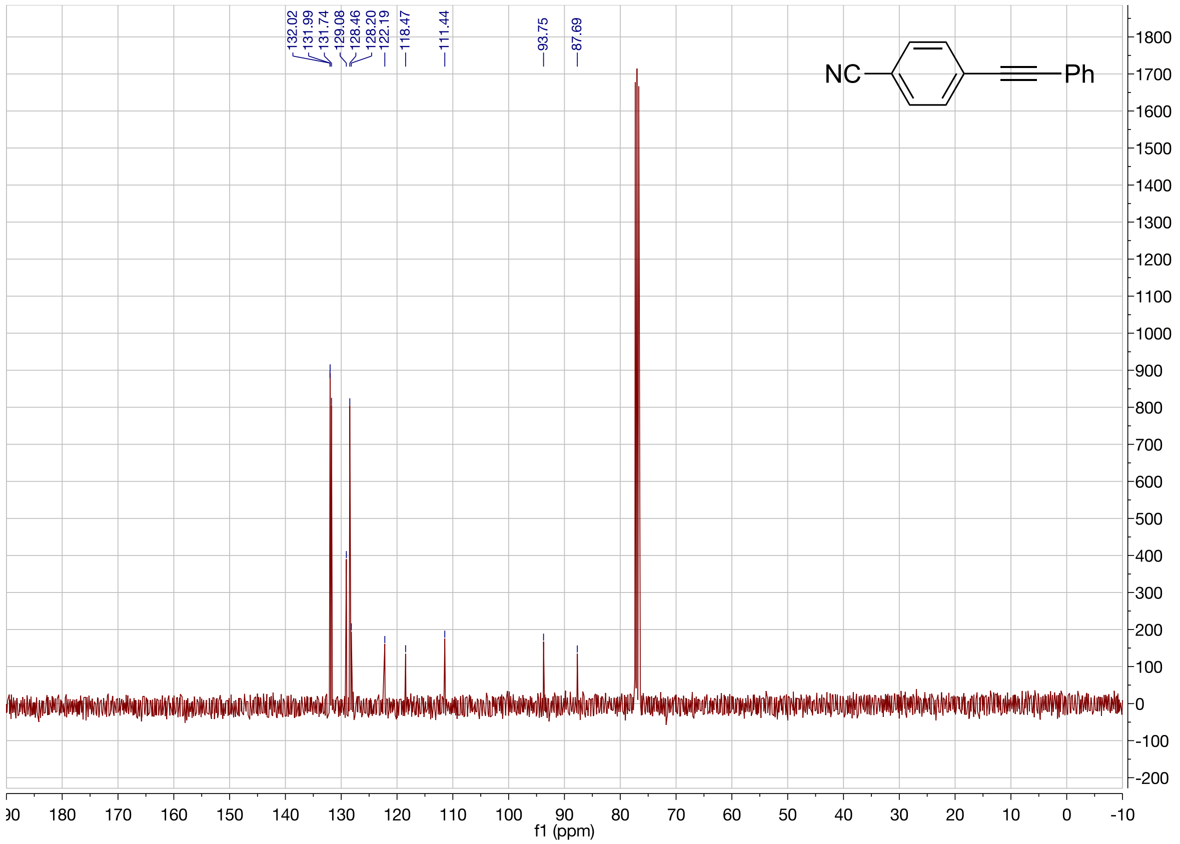
132.02
131.99
131.74
129.08
128.46
128.20
122.19
118.47
111.44
93.75
87.69
NC
Ph
f1 (ppm)

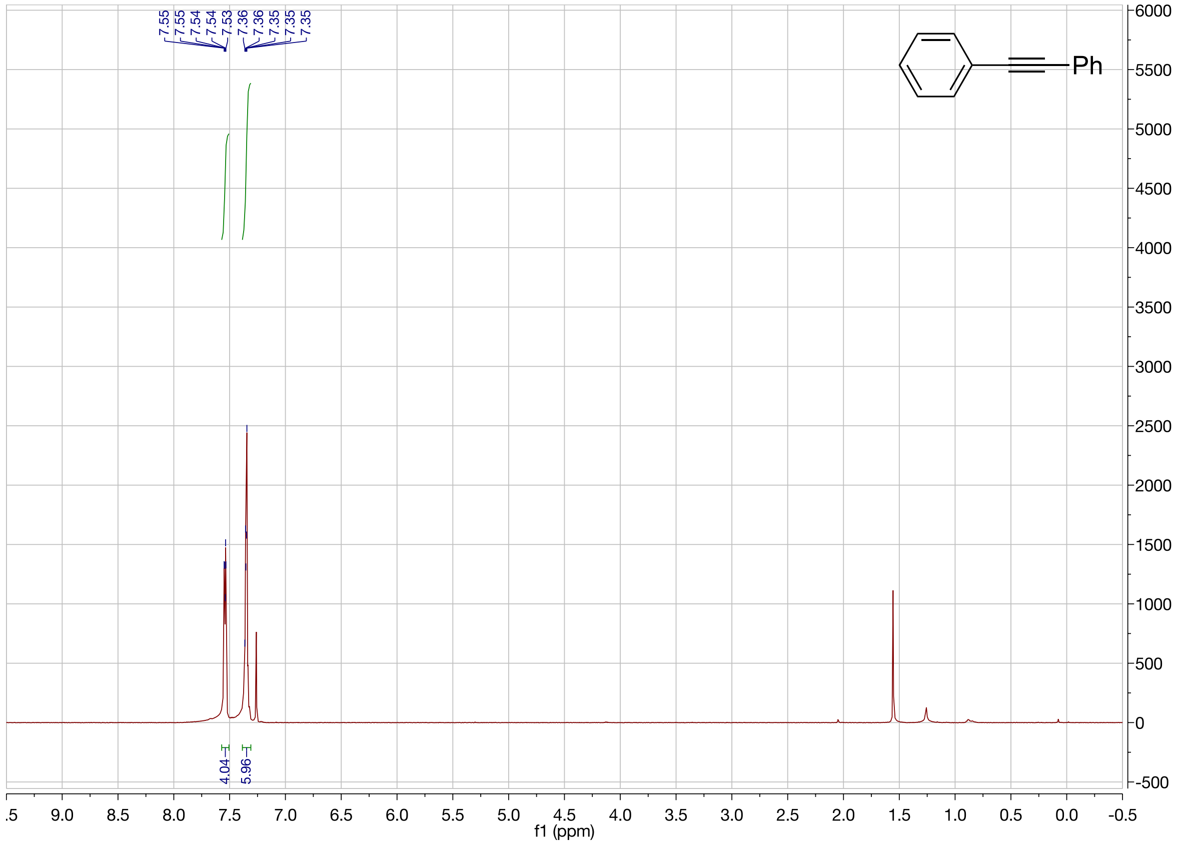
7.55
7.55
7.54
7.54
7.53
7.36
7.36
7.35
7.35
7.35
Ph
4.04
5.96
f1 (ppm)

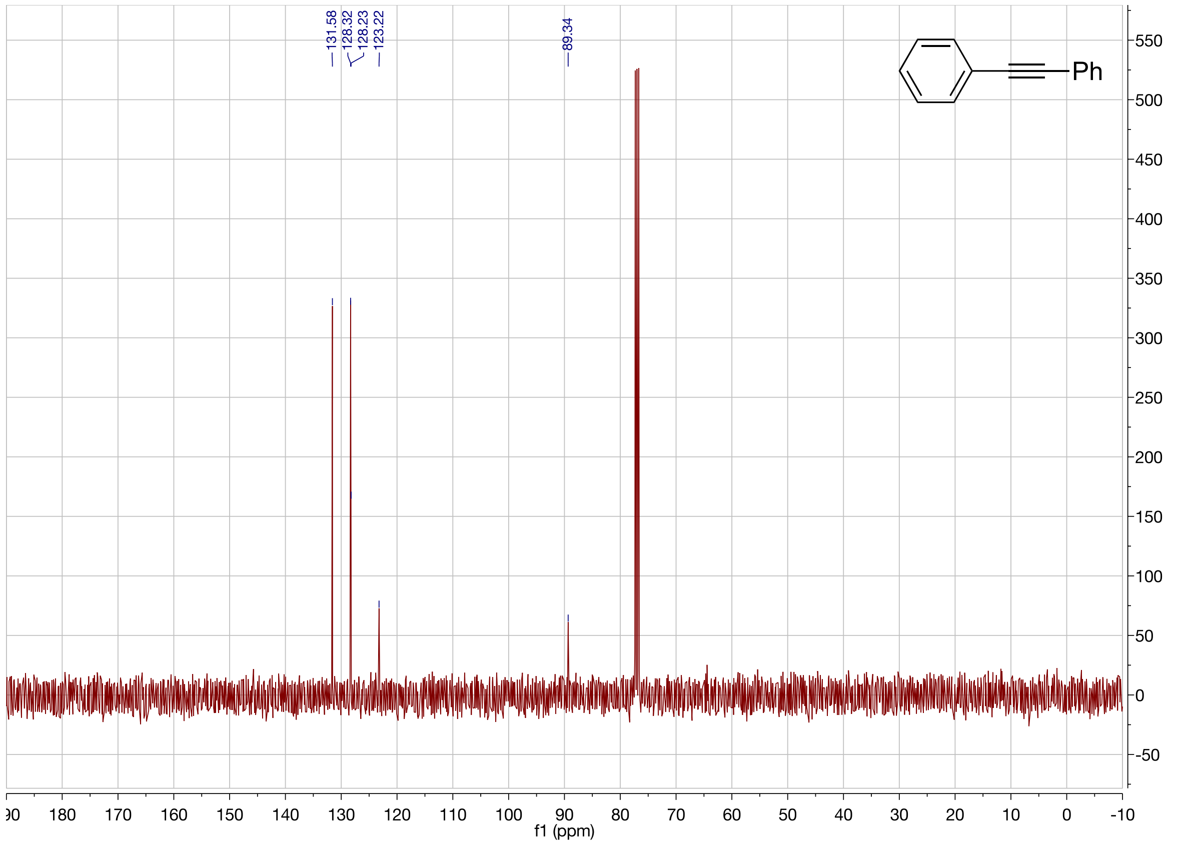
131.58
128.32
128.23
123.22
89.34
Ph
f1 (ppm)

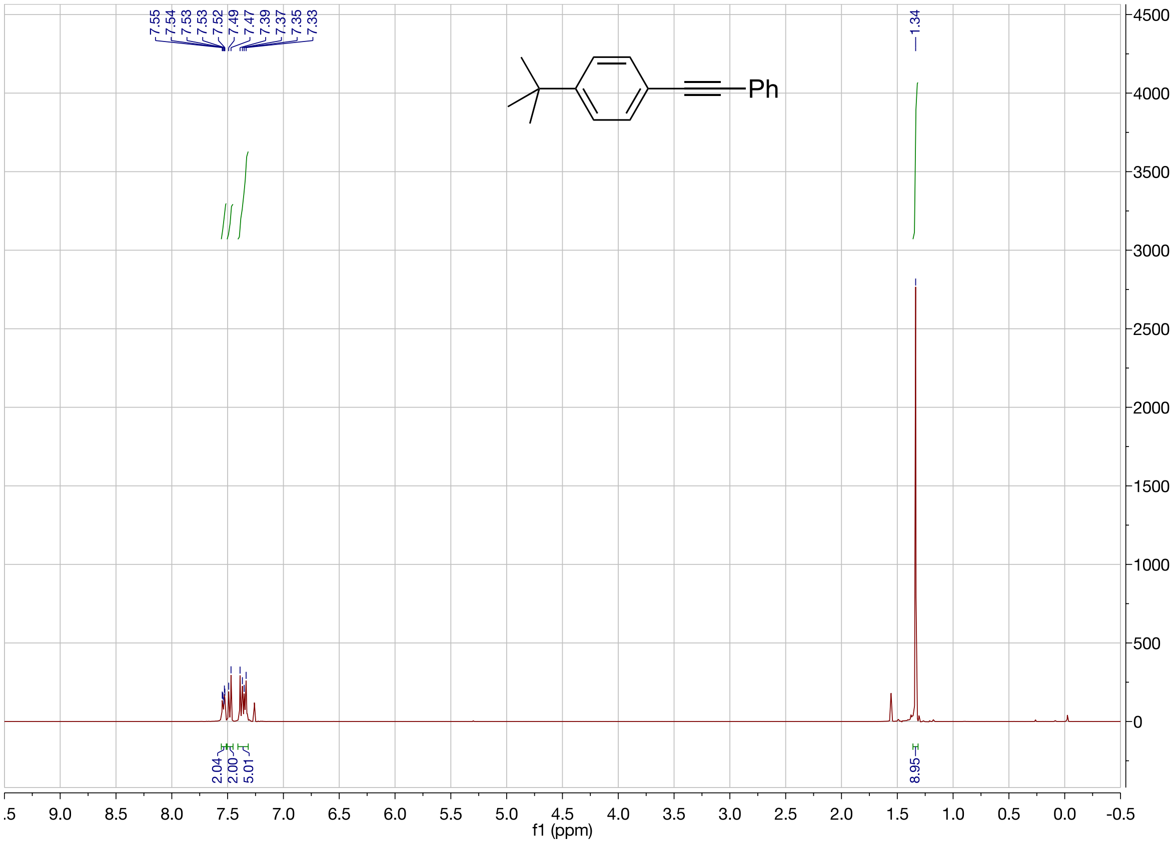
7.55
7.54
7.53
7.53
7.52
7.49
7.47
7.39
7.37
7.35
7.33
1.34
Ph
2.04
2.00
5.01
8.95
f1 (ppm)

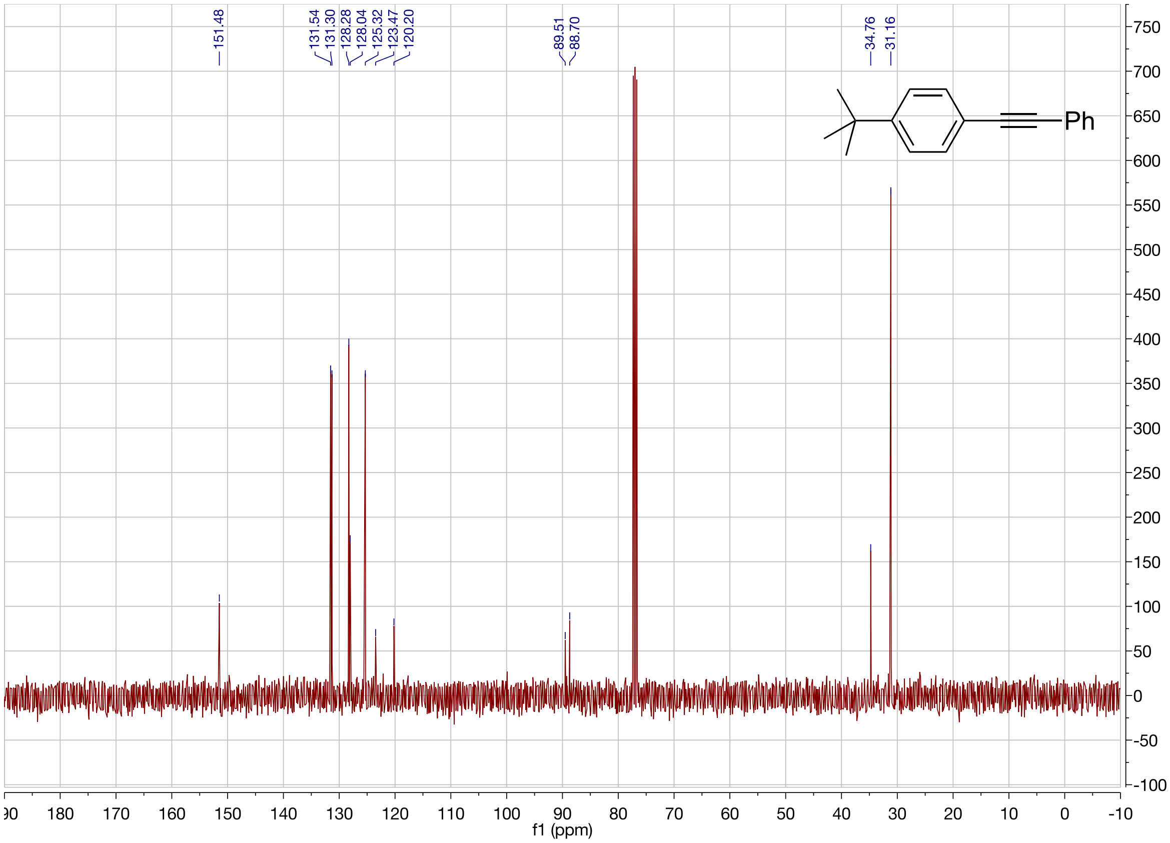
151.48
131.54
131.30
128.28
128.04
125.32
123.47
120.20
89.51
88.70
34.76
31.16
Ph
f1 (ppm)

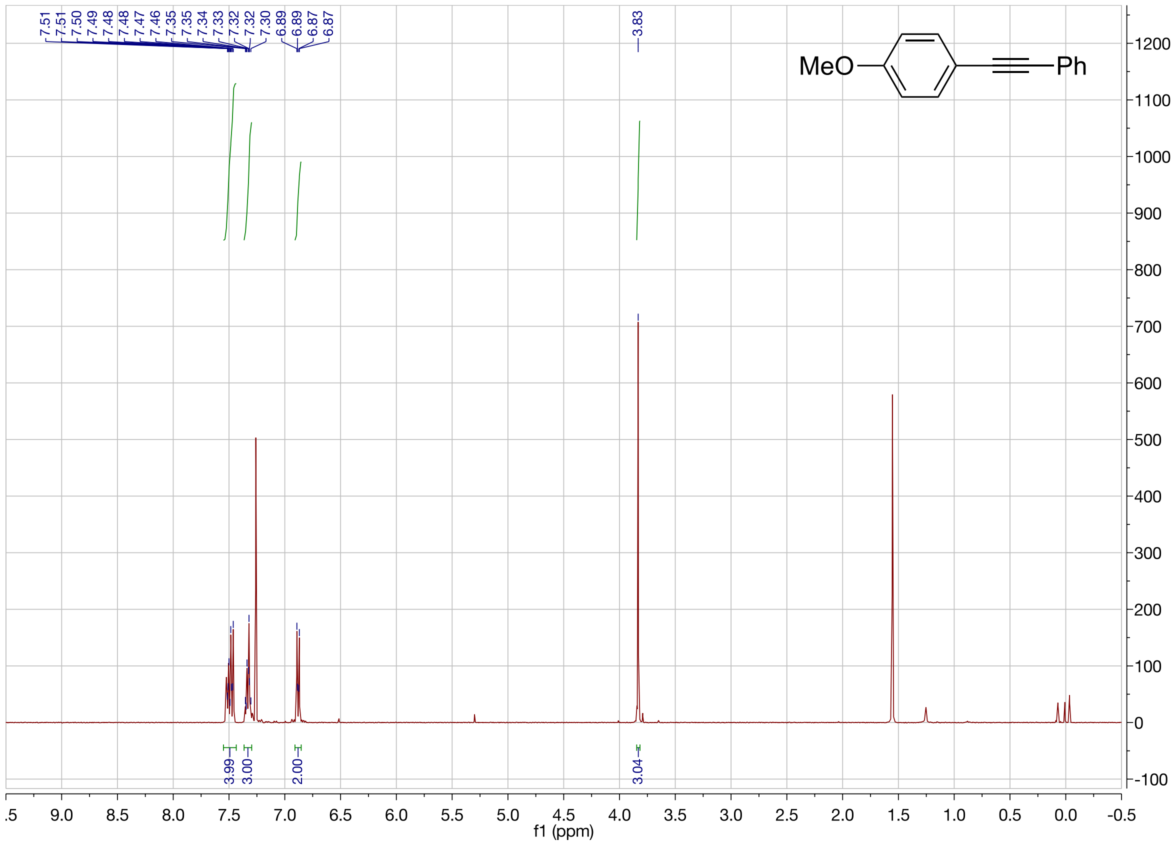
MeO
Ph
f1 (ppm)

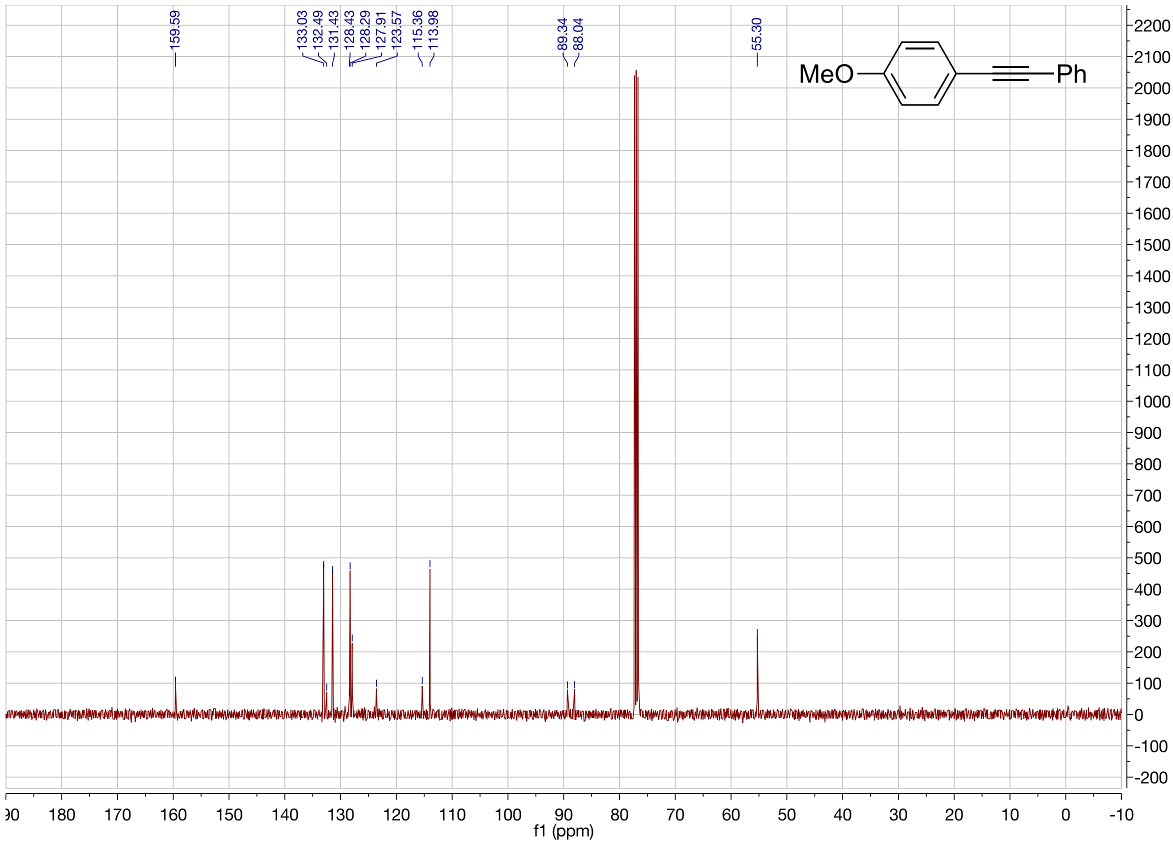
MeO
Ph
f1 (ppm)

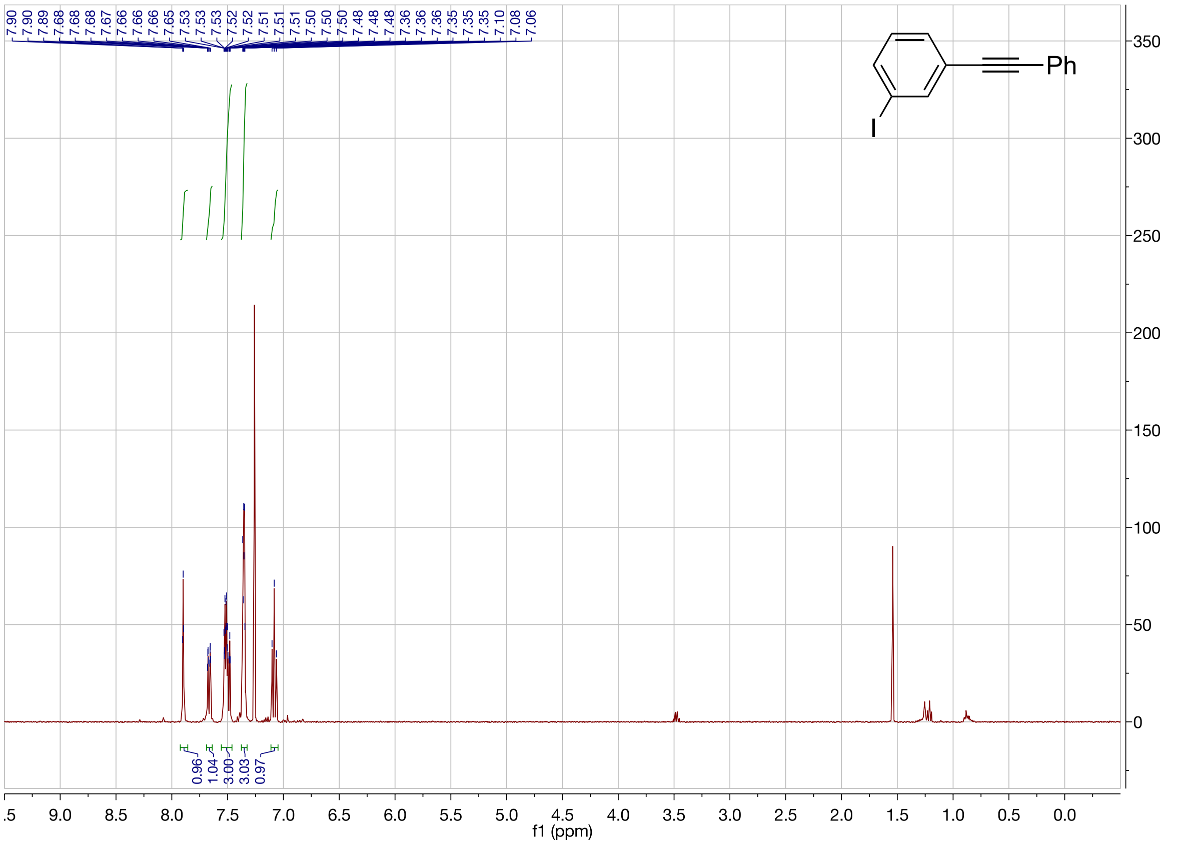
7.90
7.90
7.89
7.68
7.68
7.68
7.67
7.66
7.66
7.66
7.65
7.53
7.53
7.53
7.52
7.52
7.51
7.51
7.51
7.50
7.50
7.50
7.48
7.48
7.48
7.36
7.36
7.36
7.35
7.35
7.35
7.10
7.08
7.06
Ph
I
0.96
1.04
3.00
3.03
0.97
f1 (ppm)

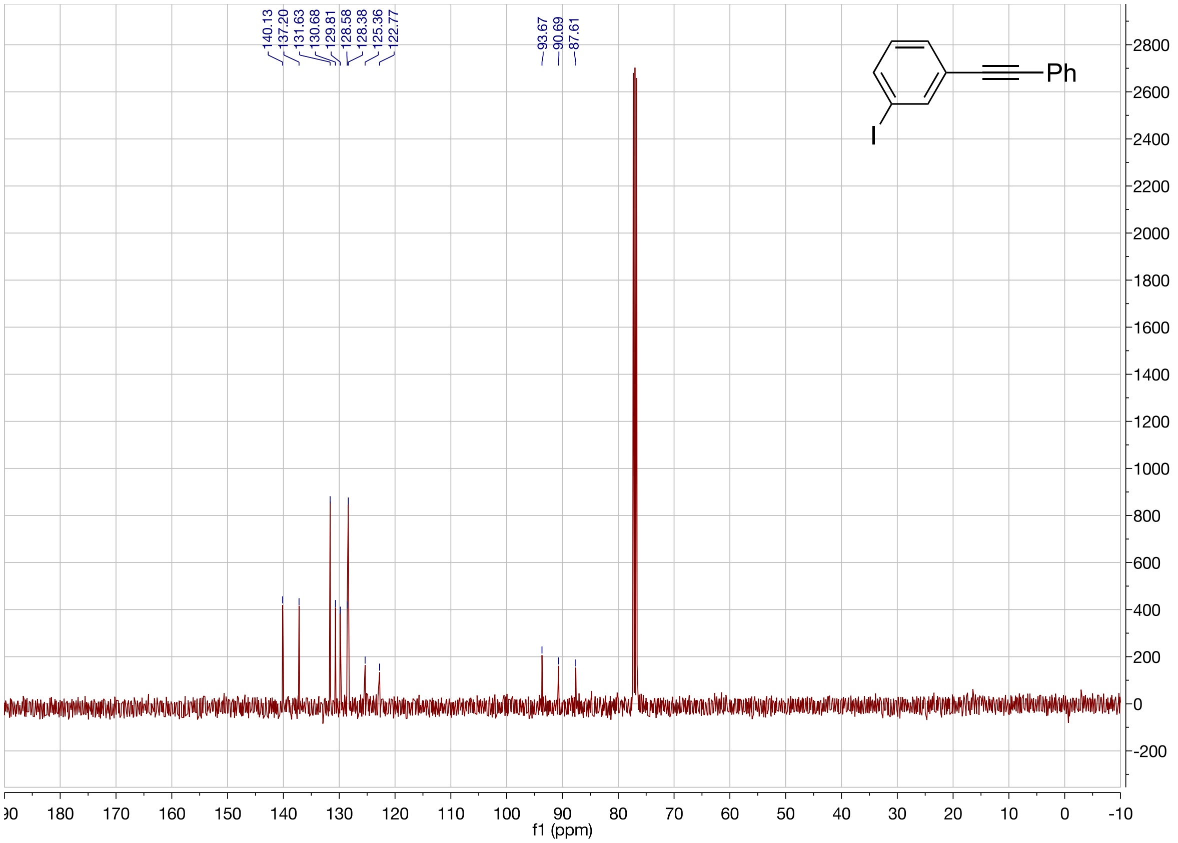
140.13
137.20
131.63
130.68
129.81
128.58
128.38
125.36
122.77
93.67
90.69
87.61
Ph
I
f1 (ppm)

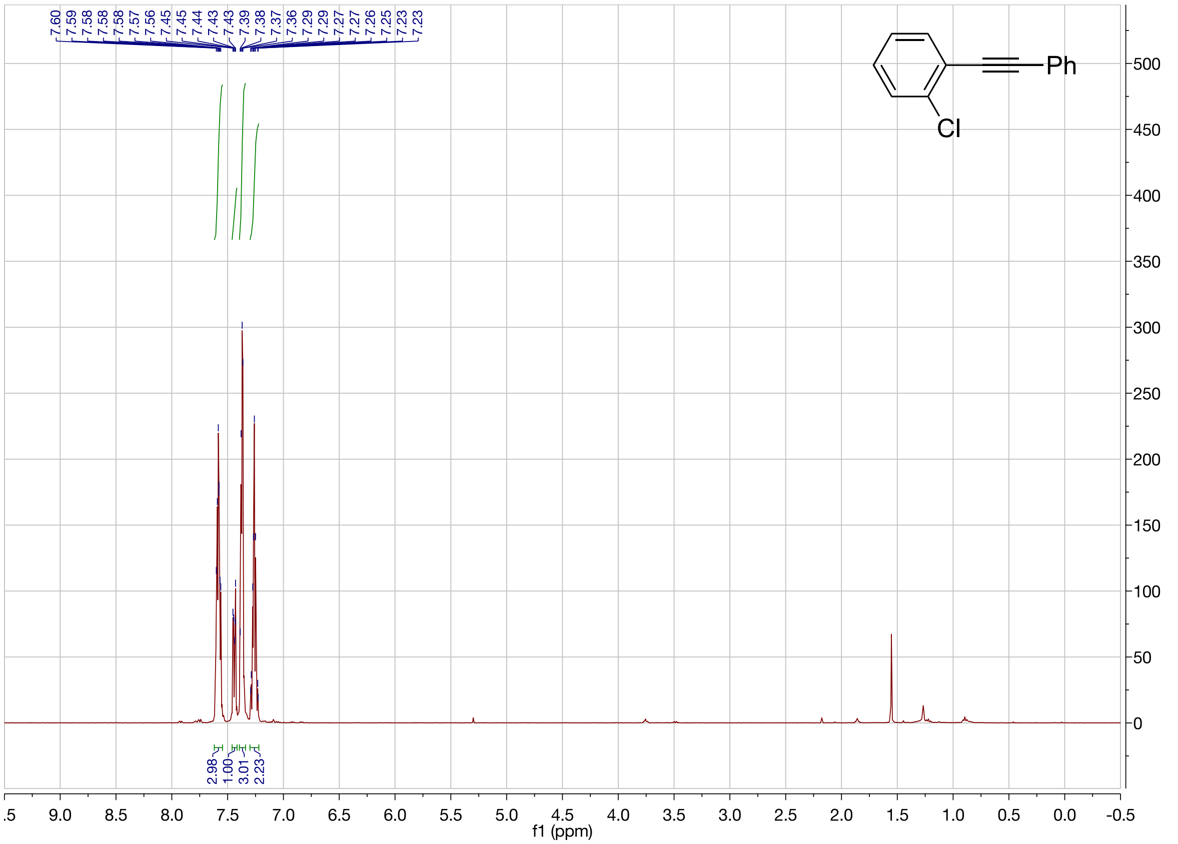
7.60
7.59
7.58
7.58
7.58
7.57
7.56
7.45
7.45
7.44
7.43
7.43
7.39
7.38
7.37
7.36
7.29
7.29
7.27
7.27
7.26
7.25
7.23
7.23
Ph
Cl
2.98
1.00
3.01
2.23
f1 (ppm)

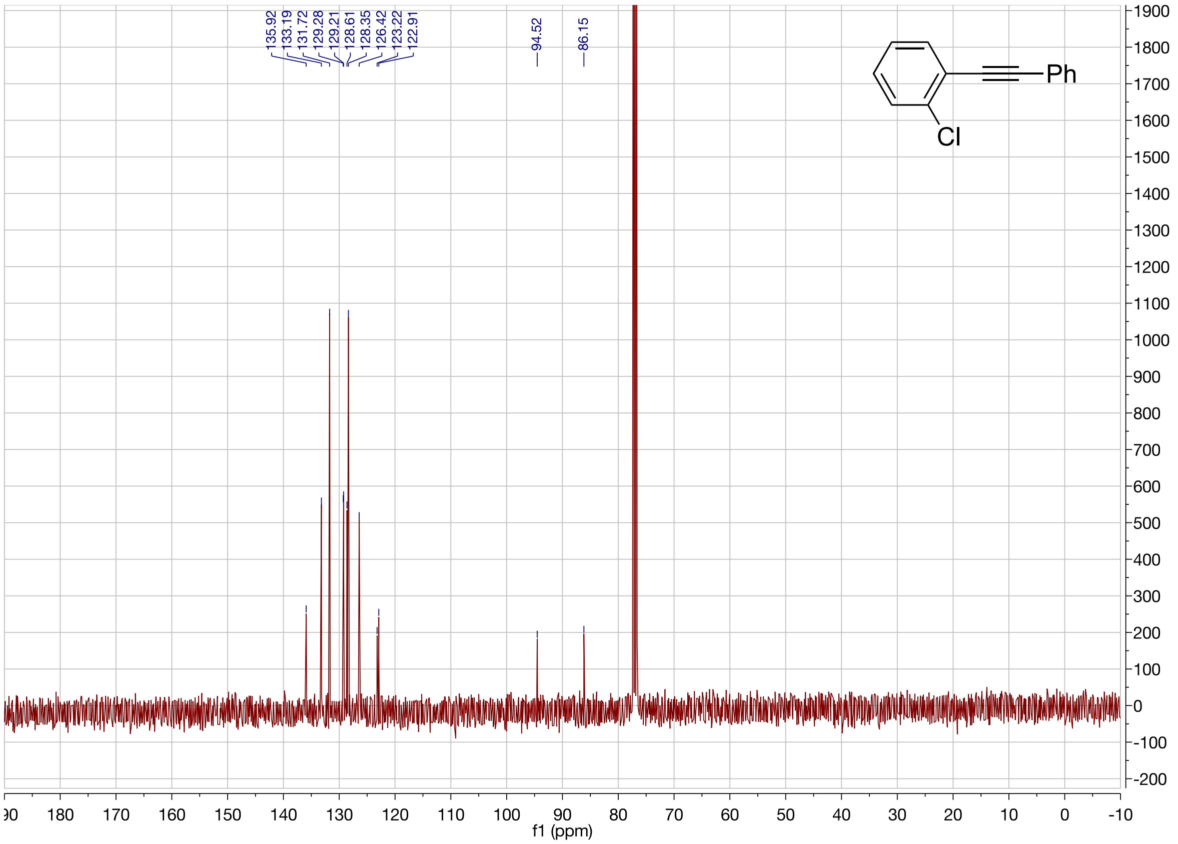
135.92
133.19
131.72
129.28
129.21
128.61
128.35
126.42
123.22
122.91
94.52
86.15
Ph
Cl
f1 (ppm)

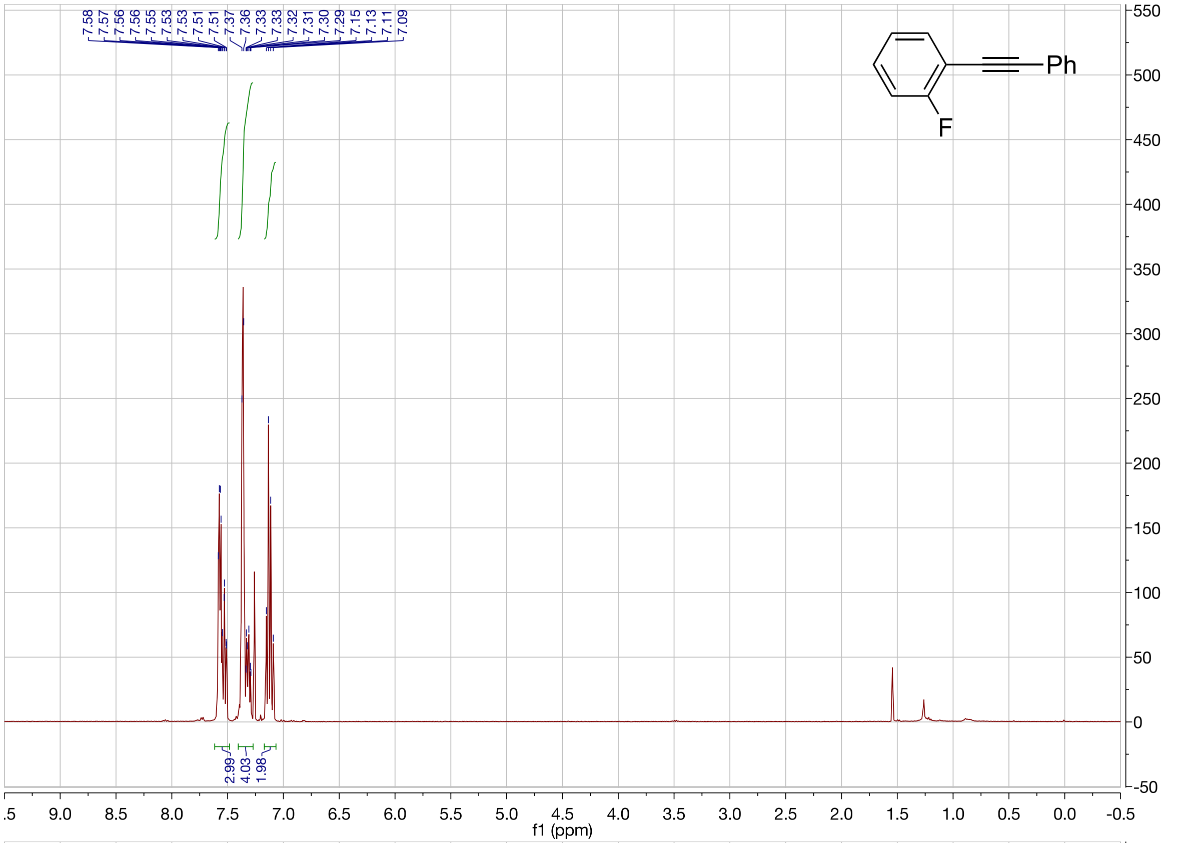
7.58
7.57
7.56
7.56
7.55
7.53
7.53
7.51
7.51
7.37
7.36
7.33
7.33
7.32
7.31
7.30
7.29
7.15
7.13
7.11
7.09
Ph
F
2.99
4.03
1.98
f1 (ppm)

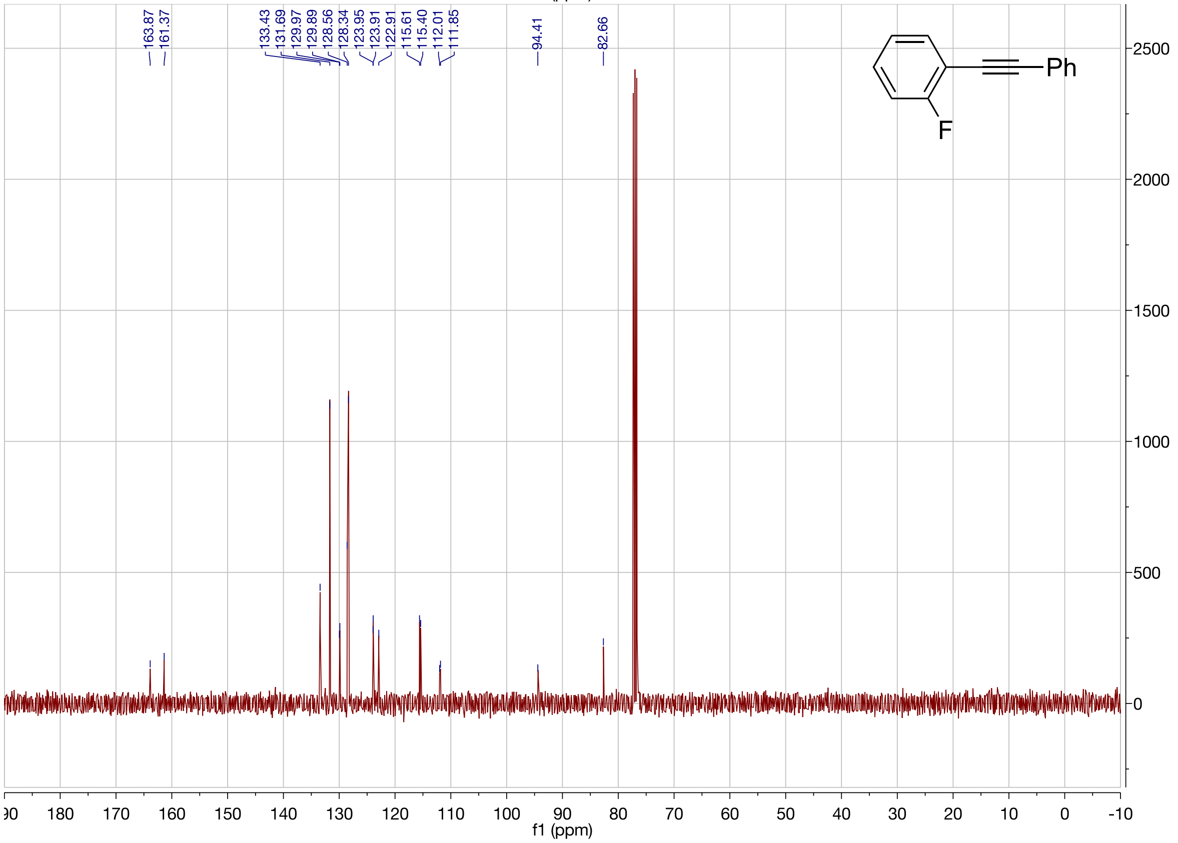
163.87
161.37
133.43
131.69
129.97
129.89
128.56
128.34
123.95
123.91
122.91
115.61
115.40
112.01
111.85
94.41
82.66
Ph
F
f1 (ppm)

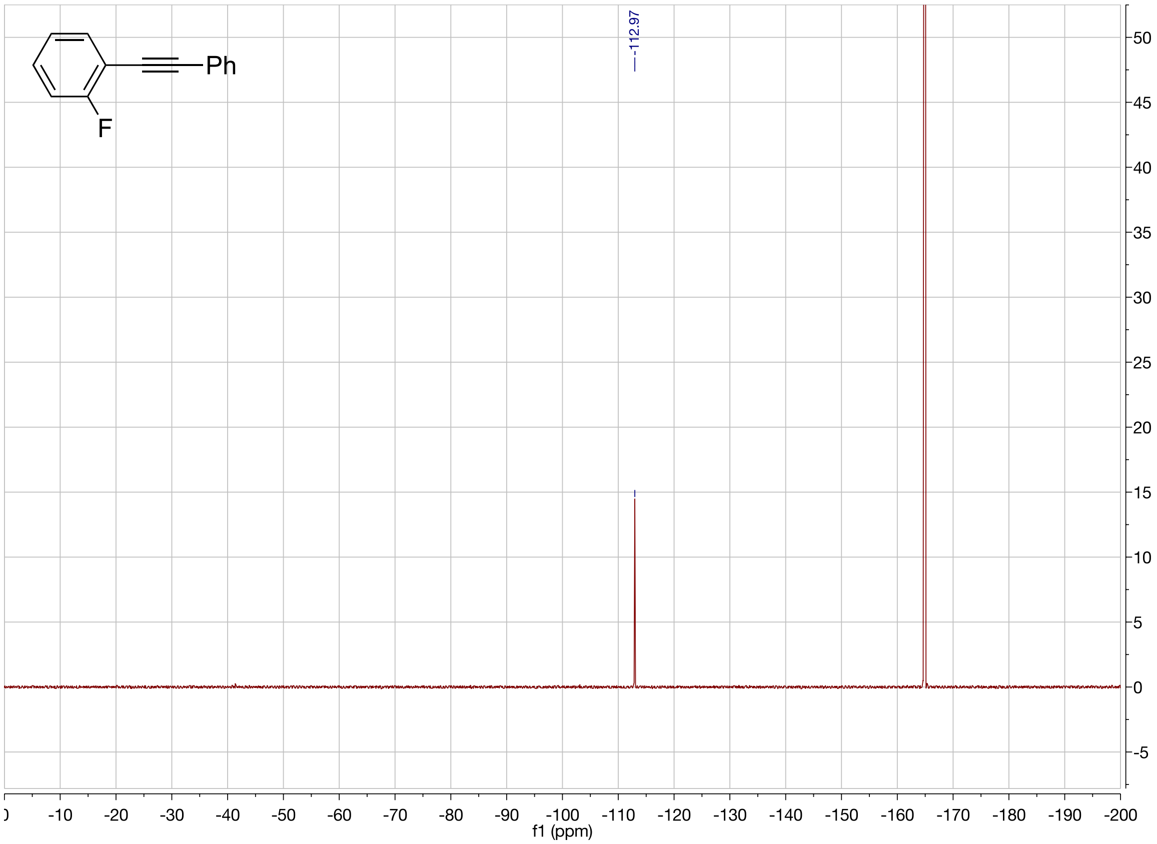

Ph
F
-112.97
f1 (ppm)

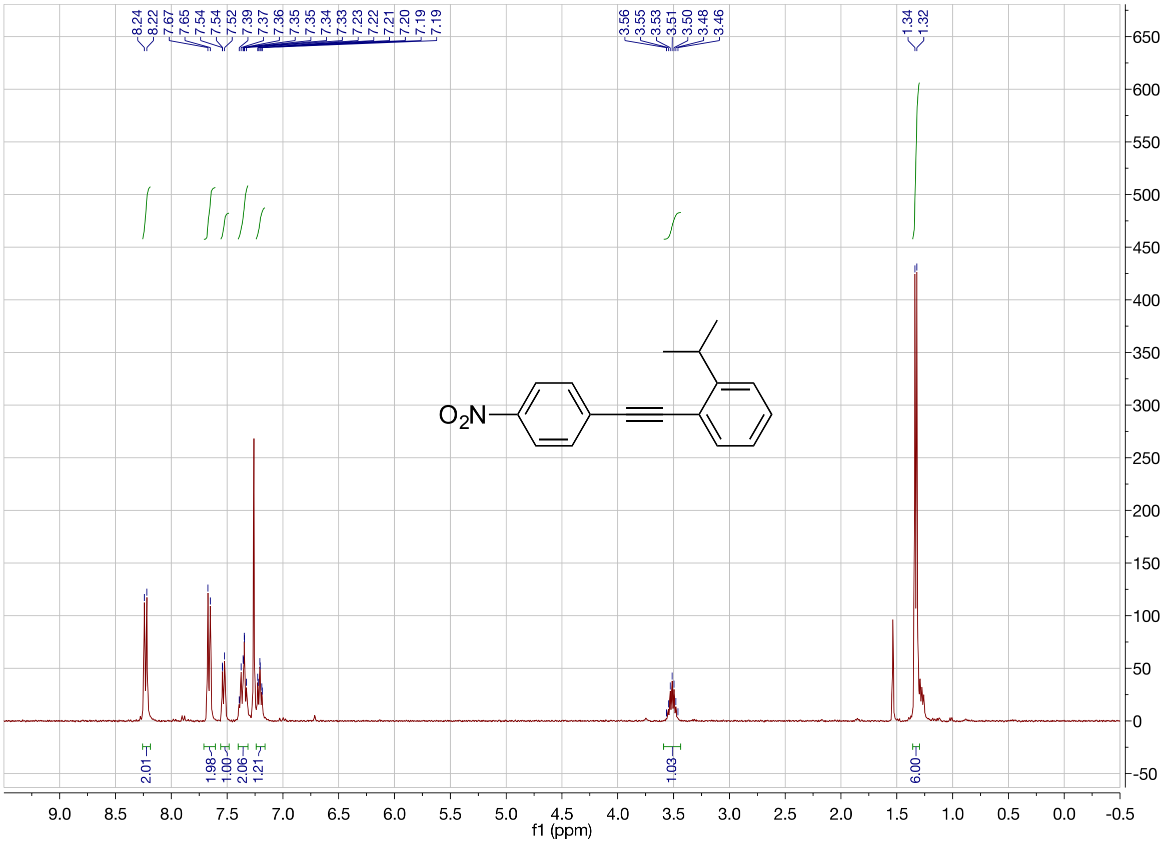
8.24
8.22
7.67
7.65
7.54
7.54
7.52
7.39
7.37
7.36
7.35
7.35
7.34
7.33
7.23
7.22
7.21
7.20
7.19
7.19
3.56
3.55
3.53
3.51
3.50
3.48
3.46
1.34
1.32
O_2N
2.01
1.98
1.00
2.06
1.21
1.03
6.00
f1 (ppm)

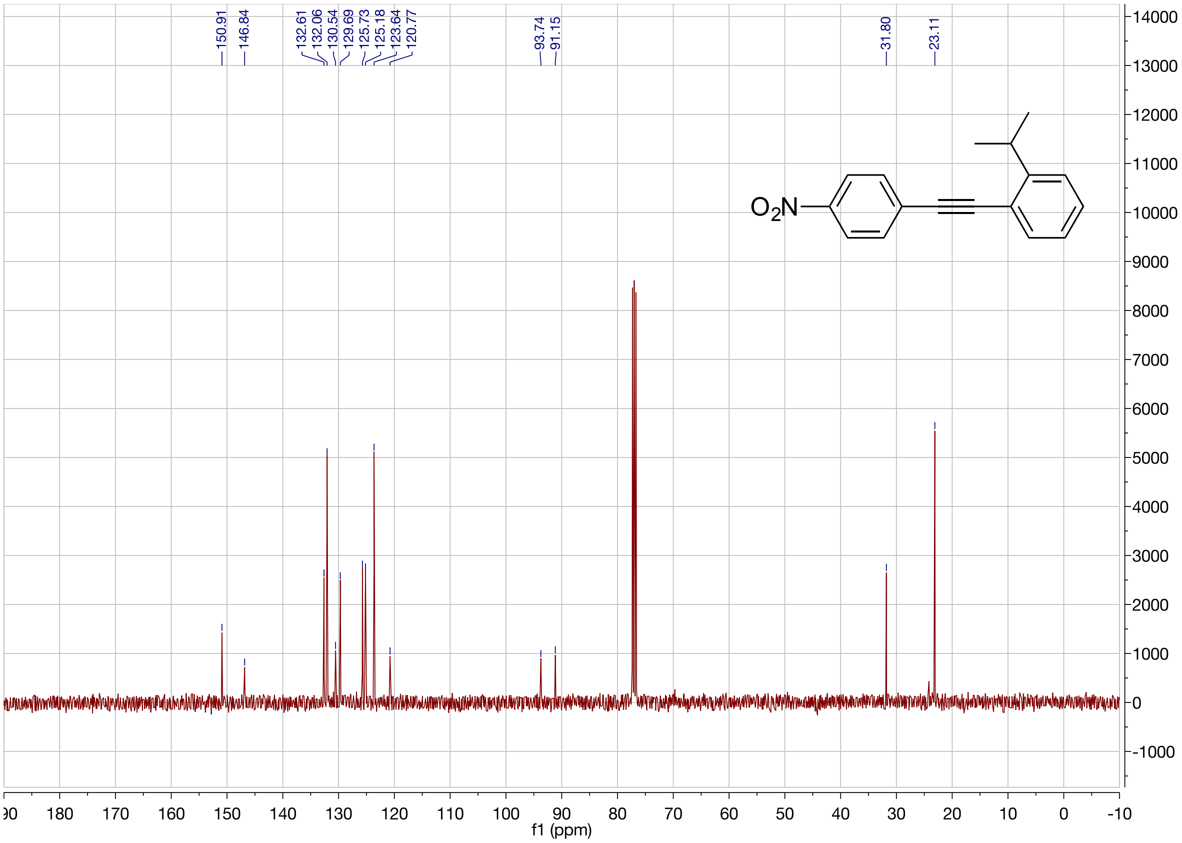
150.91
146.84
132.61
132.06
130.54
129.69
125.73
125.18
123.64
120.77
93.74
91.15
31.80
23.11
O_2N
f1 (ppm)

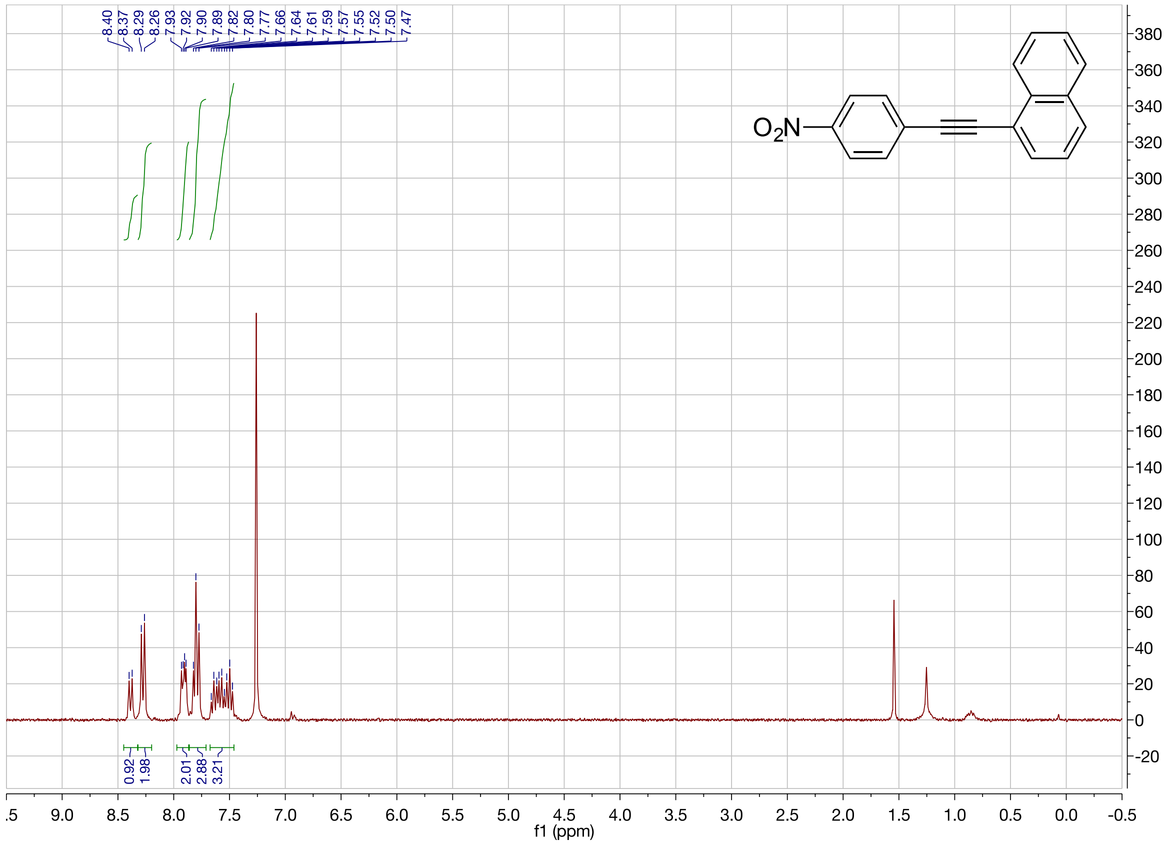

8.40
8.37
8.29
8.26
7.93
7.92
7.90
7.89
7.82
7.80
7.77
7.66
7.64
7.61
7.59
7.57
7.55
7.52
7.50
7.47
O_2N
0.92
1.98
2.01
2.88
3.21
f1 (ppm)

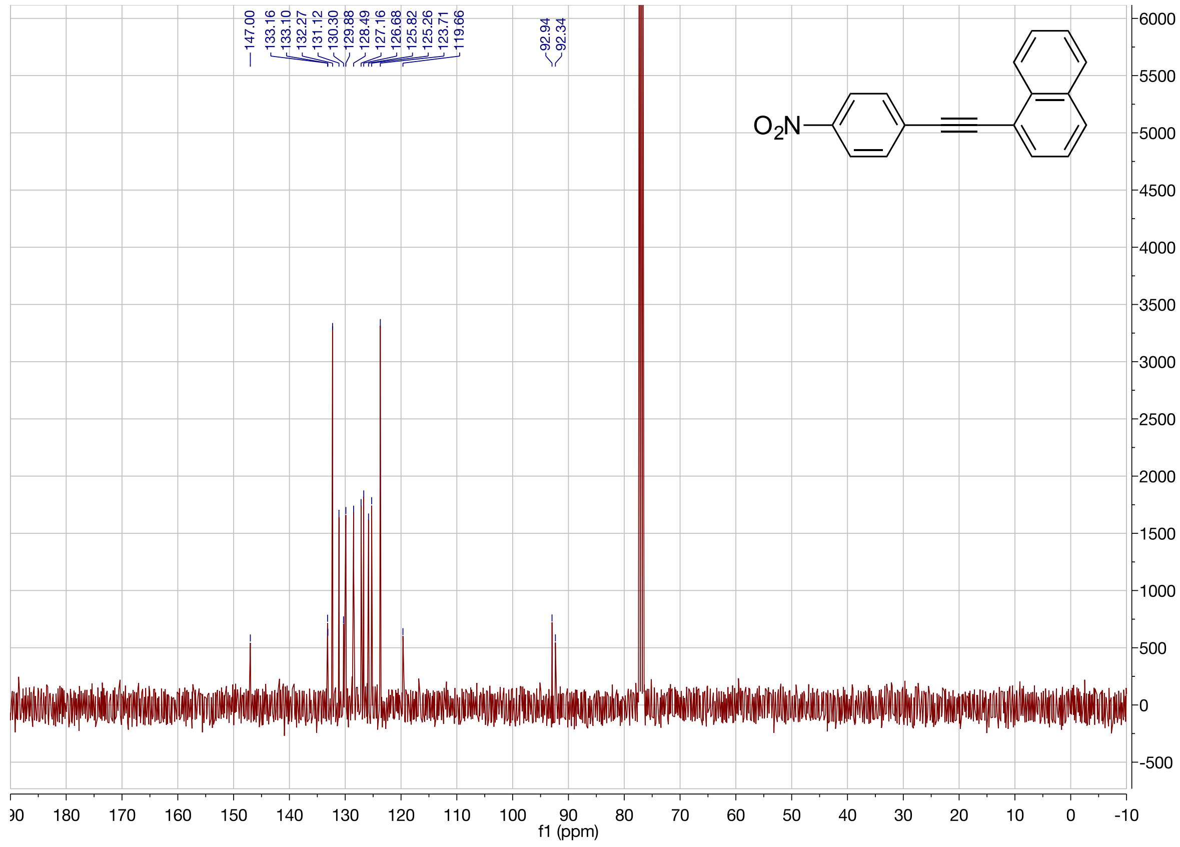

147.00
133.16
133.10
132.27
131.12
130.30
129.88
128.49
127.16
126.68
125.82
125.26
123.71
119.66
92.94
92.34
O_2N
f1 (ppm)

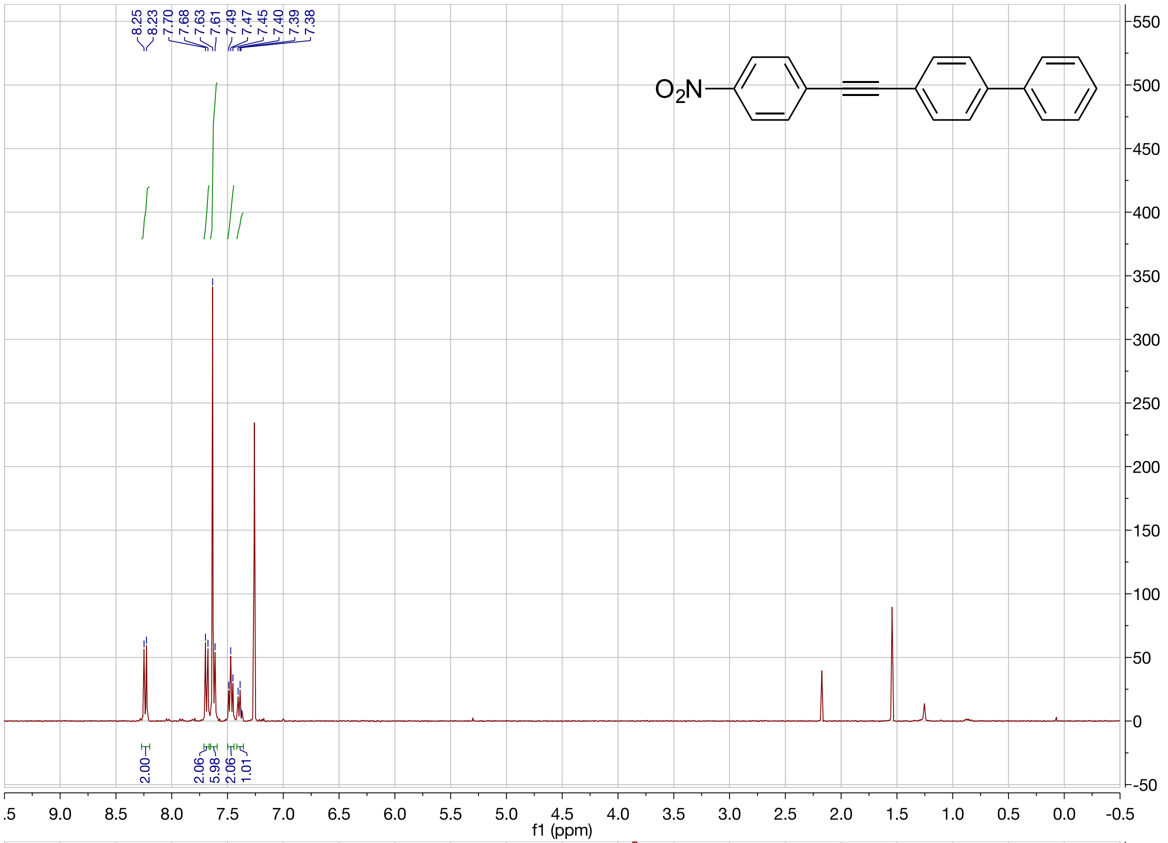
8.25
8.23
7.70
7.68
7.63
7.61
7.49
7.47
7.45
7.40
7.39
7.38
O_2N
2.00
2.06
5.98
2.06
1.01
f1 (ppm)

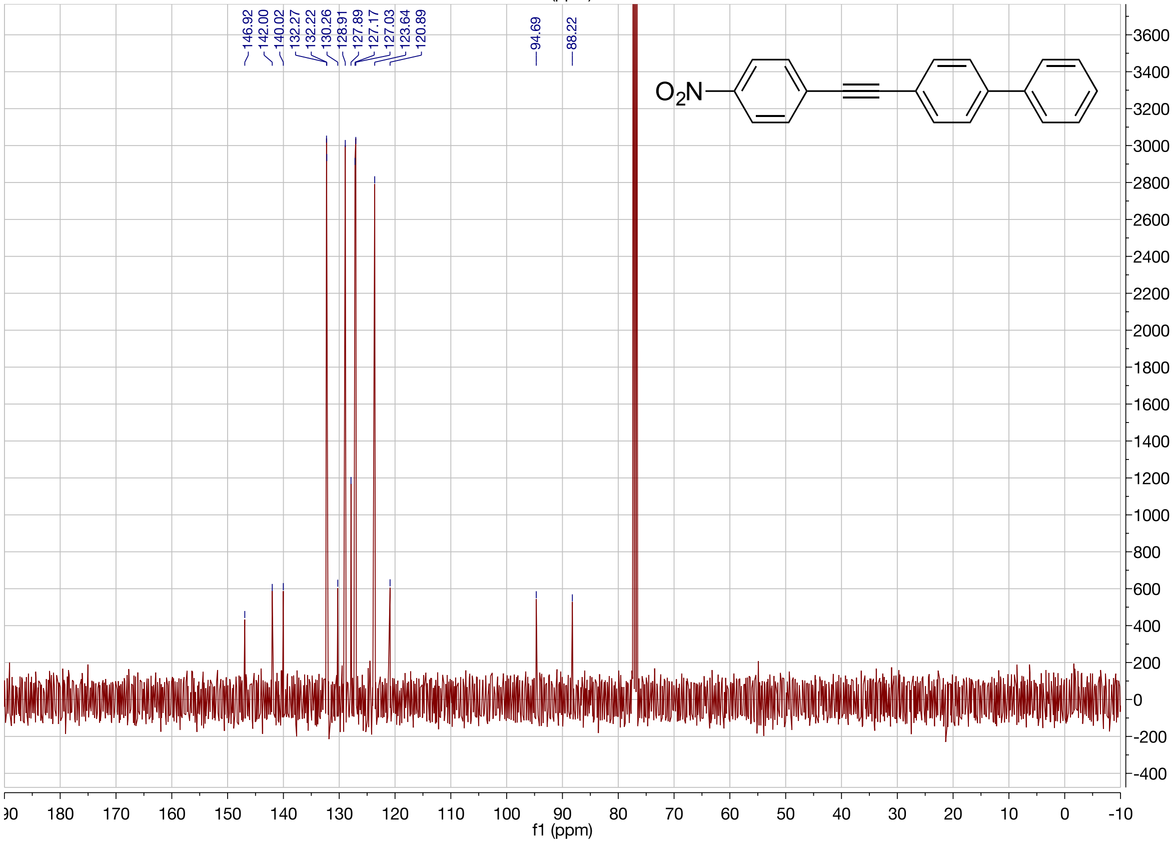
146.92
142.00
140.02
132.27
132.22
130.26
128.91
127.89
127.17
127.03
123.64
120.89
94.69
88.22
O_2N
f1 (ppm)

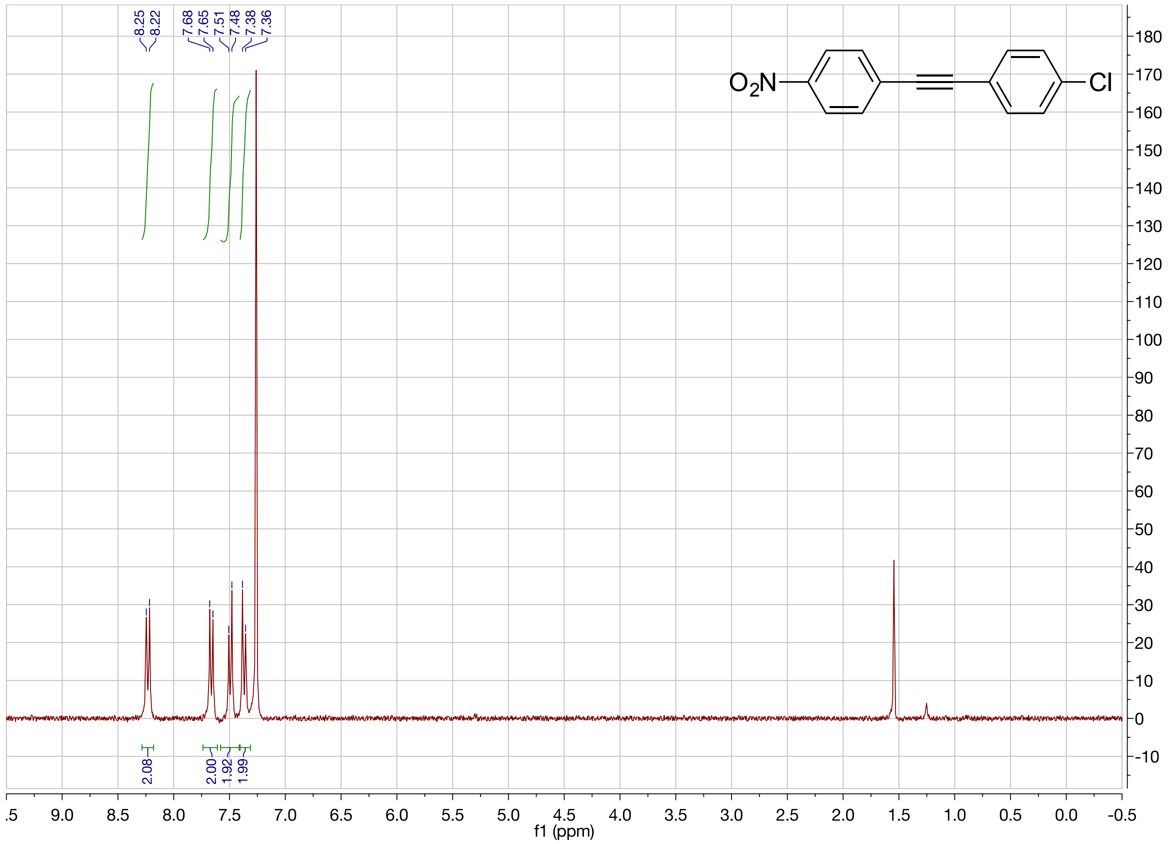
8.25
8.22
7.68
7.65
7.51
7.48
7.38
7.36
O_2N
Cl
2.08
2.00
1.92
1.99
f1 (ppm)

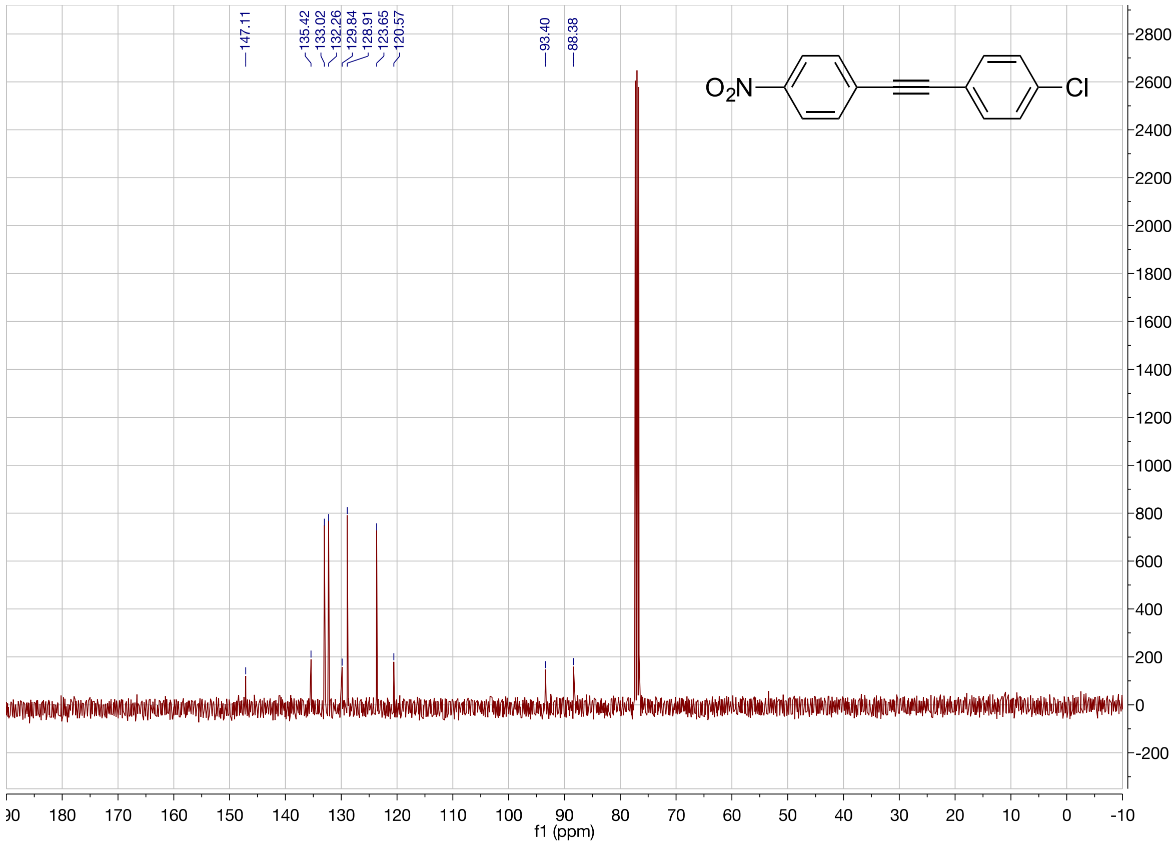
147.11
135.42
133.02
132.26
129.84
128.91
123.65
120.57
93.40
88.38
O_2N
Cl
f1 (ppm)

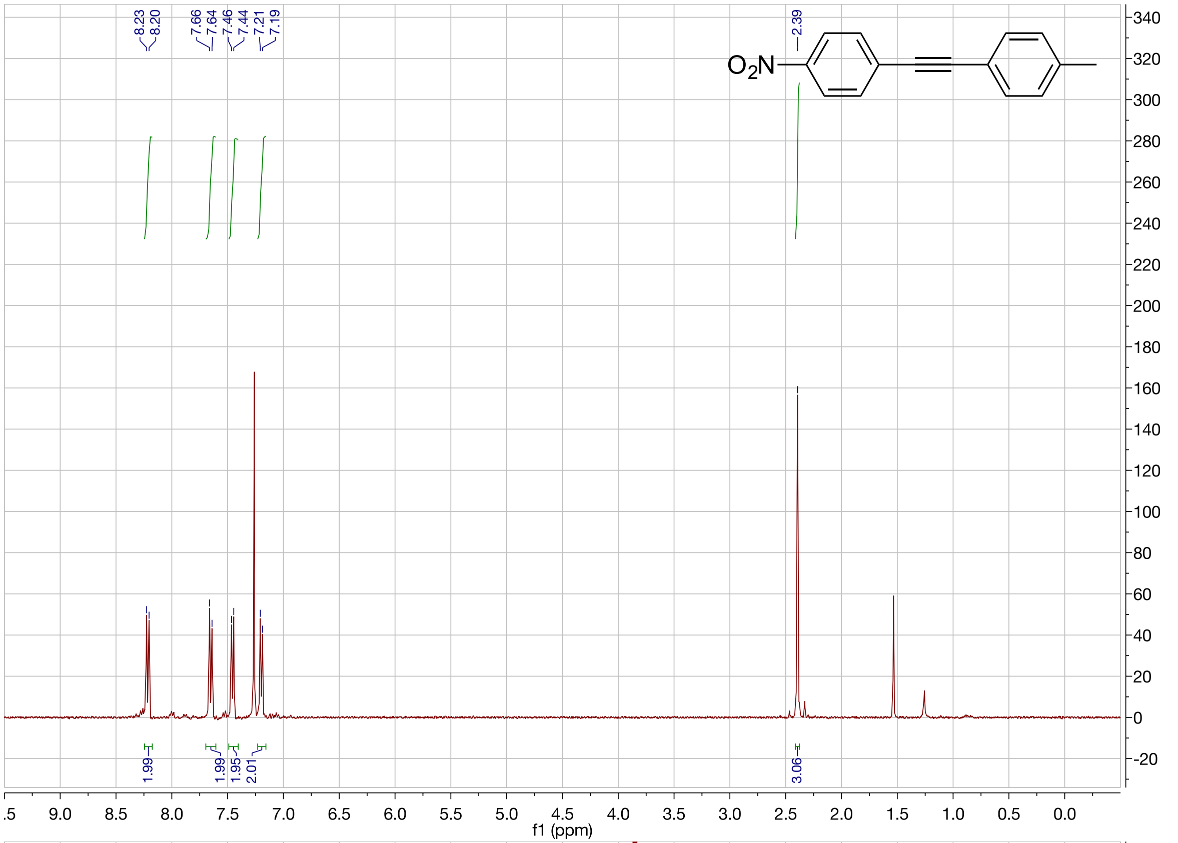
8.23
8.20
7.66
7.64
7.46
7.44
7.21
7.19
2.39
O_2N
1.99
1.99
1.95
2.01
3.06
f1 (ppm)

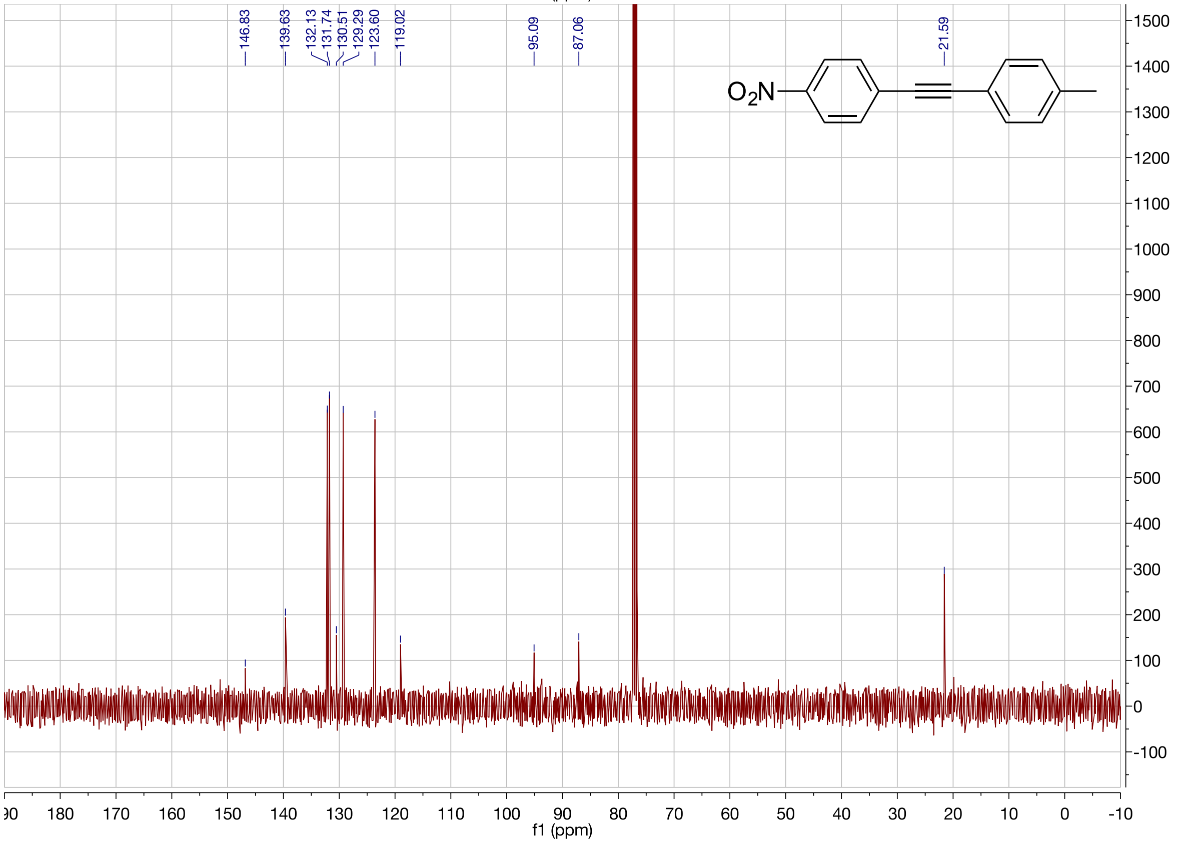
146.83
139.63
132.13
131.74
130.51
129.29
123.60
119.02
95.09
87.06
21.59
O_2N
f1 (ppm)

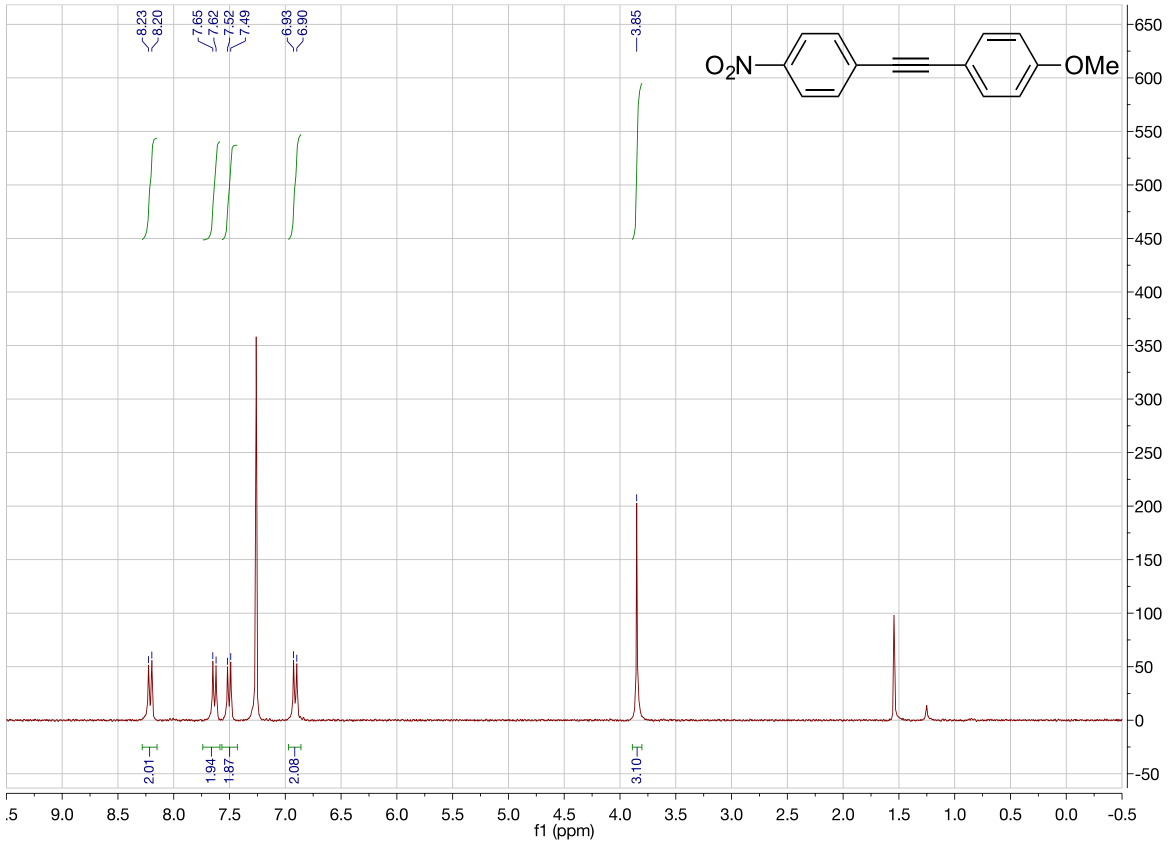

8.23
8.20
7.65
7.62
7.52
7.49
6.93
6.90
3.85
O_2N
OMe
2.01
1.94
1.87
2.08
3.10
f1 (ppm)

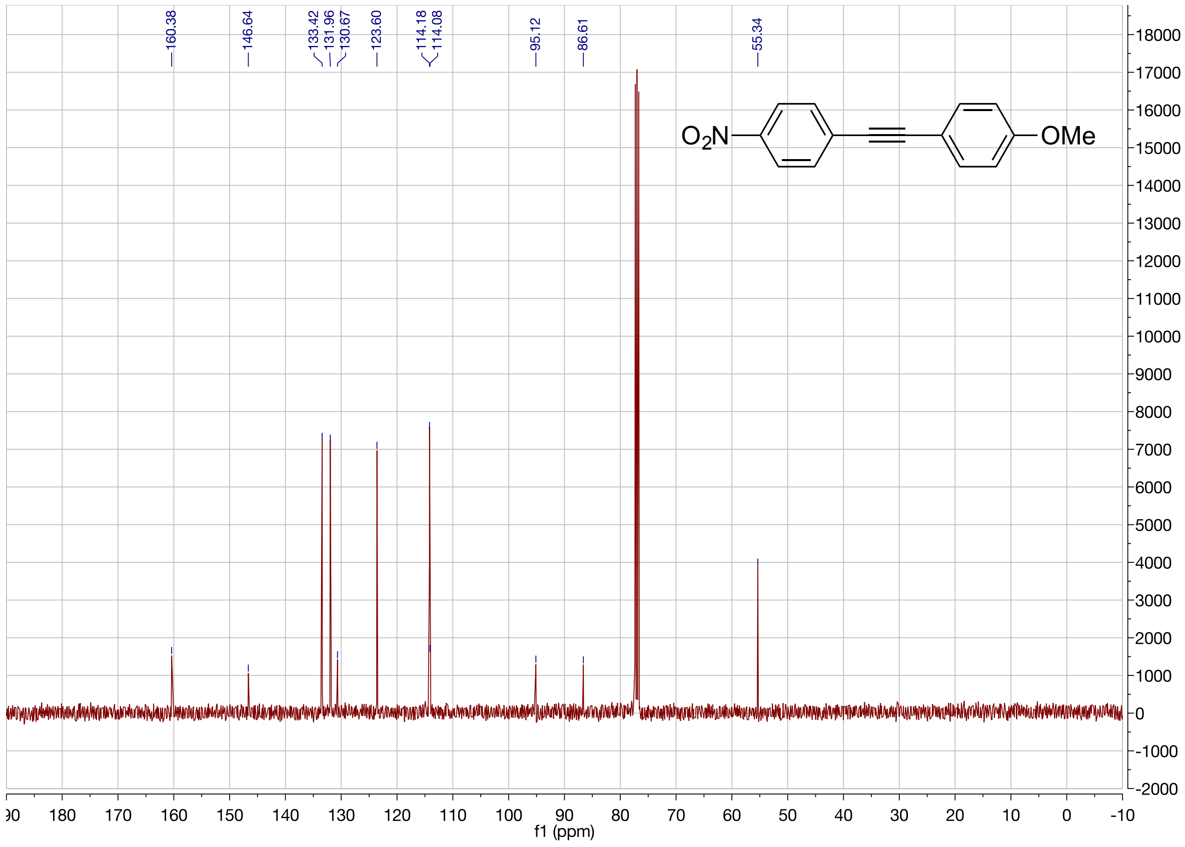

160.38
146.64
133.42
131.96
130.67
123.60
114.18
114.08
95.12
86.61
55.34
O_2N
OMe
f1 (ppm)

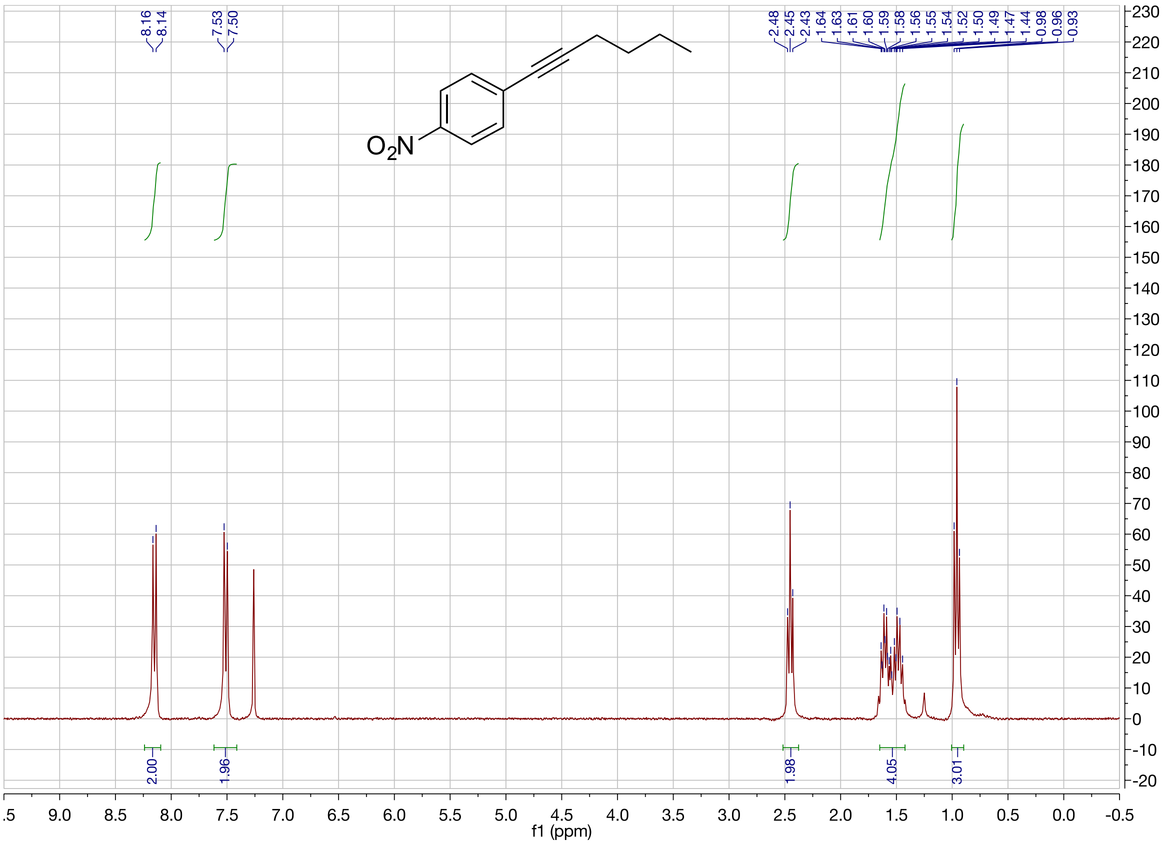
8.16
8.14
7.53
7.50
2.48
2.45
2.43
1.64
1.63
1.61
1.60
1.59
1.58
1.56
1.55
1.54
1.52
1.50
1.49
1.47
1.44
0.98
0.96
0.93
O2N
2.00
1.96
1.98
4.05
3.01
f1 (ppm)

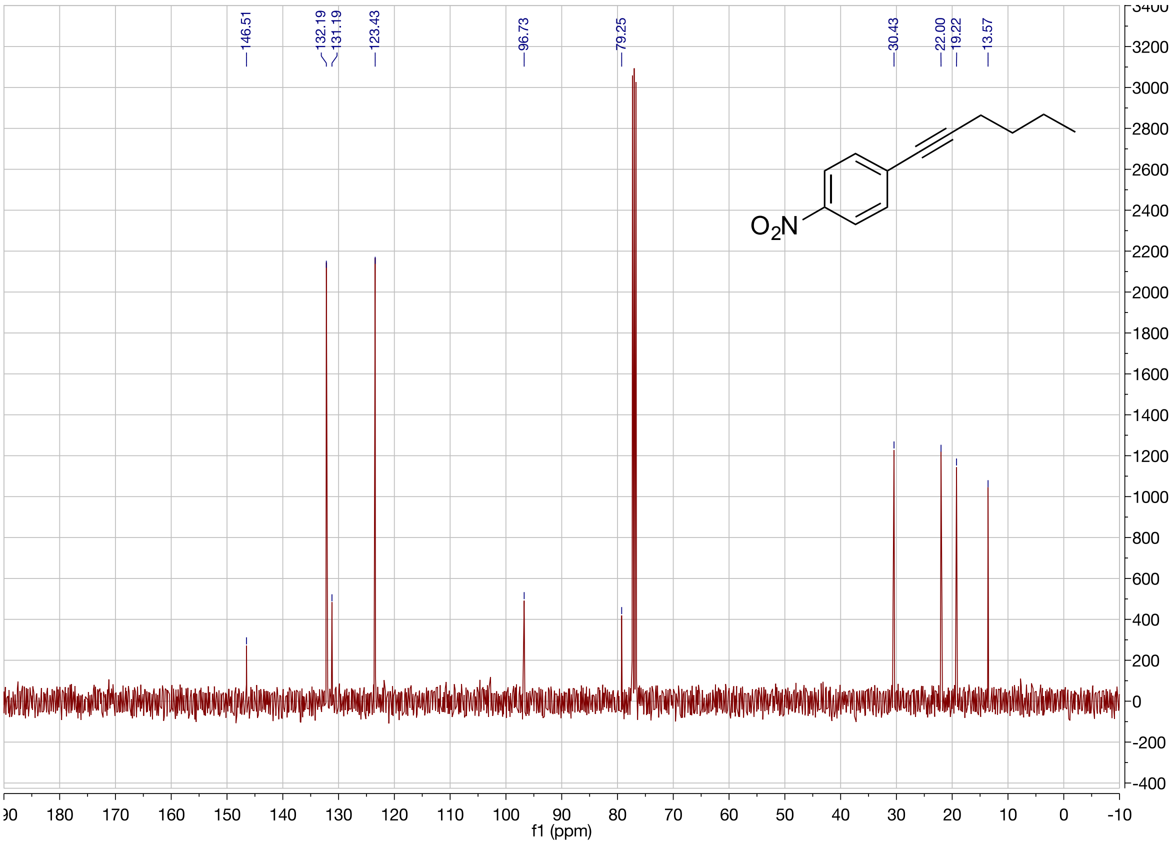
146.51
132.19
131.19
123.43
96.73
79.25
30.43
22.00
19.22
13.57
O2N
f1 (ppm)

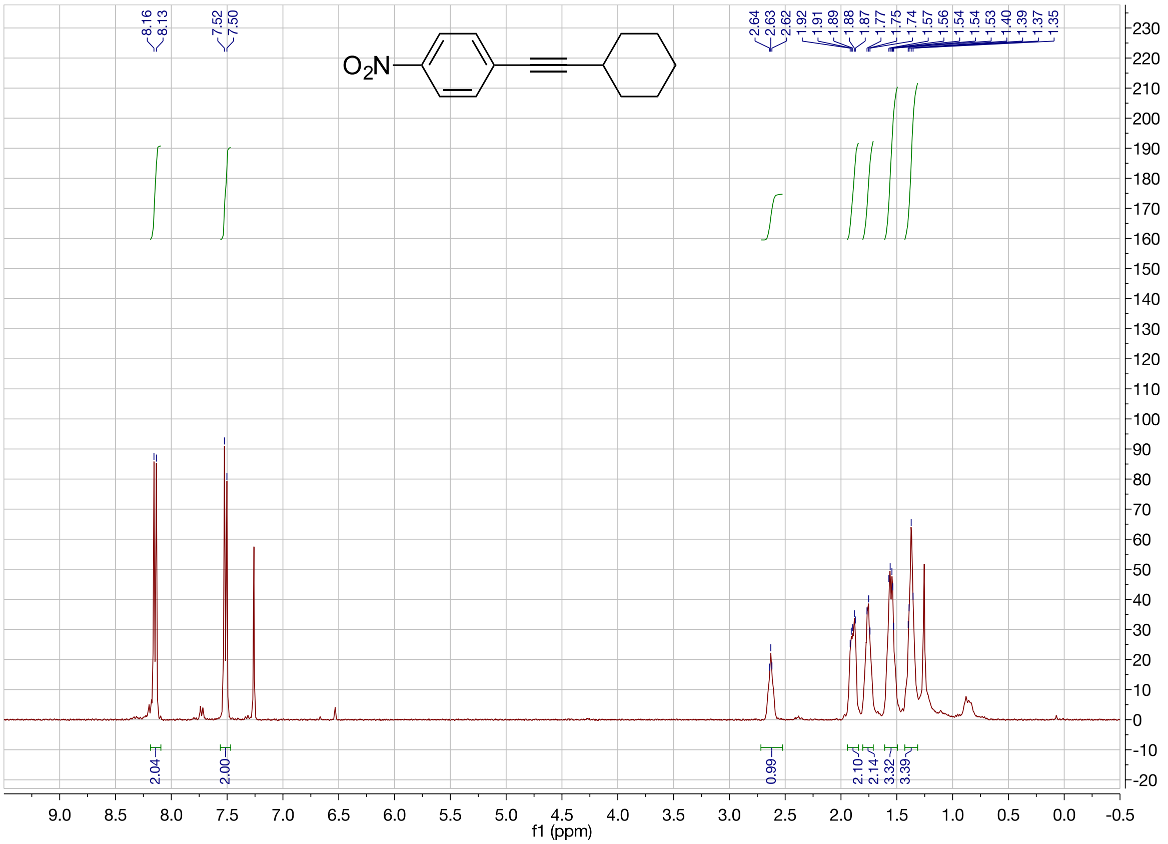
8.16
8.13
7.52
7.50
2.64
2.63
2.62
1.92
1.91
1.89
1.88
1.87
1.77
1.75
1.74
1.57
1.56
1.54
1.54
1.53
1.40
1.39
1.37
1.35
O2N
2.04
2.00
0.99
2.10
2.14
3.32
3.39
f1 (ppm)

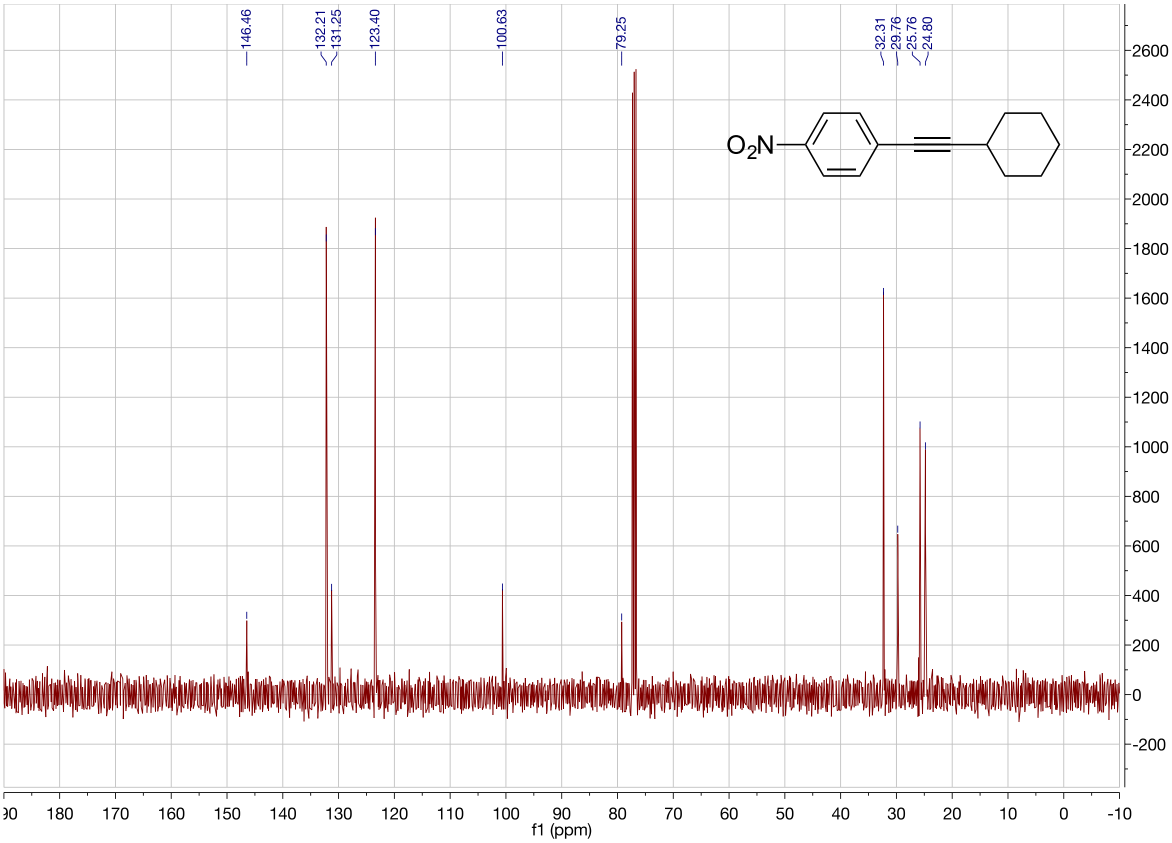
146.46
132.21
131.25
123.40
100.63
79.25
32.31
29.76
25.76
24.80
O2N
f1 (ppm)

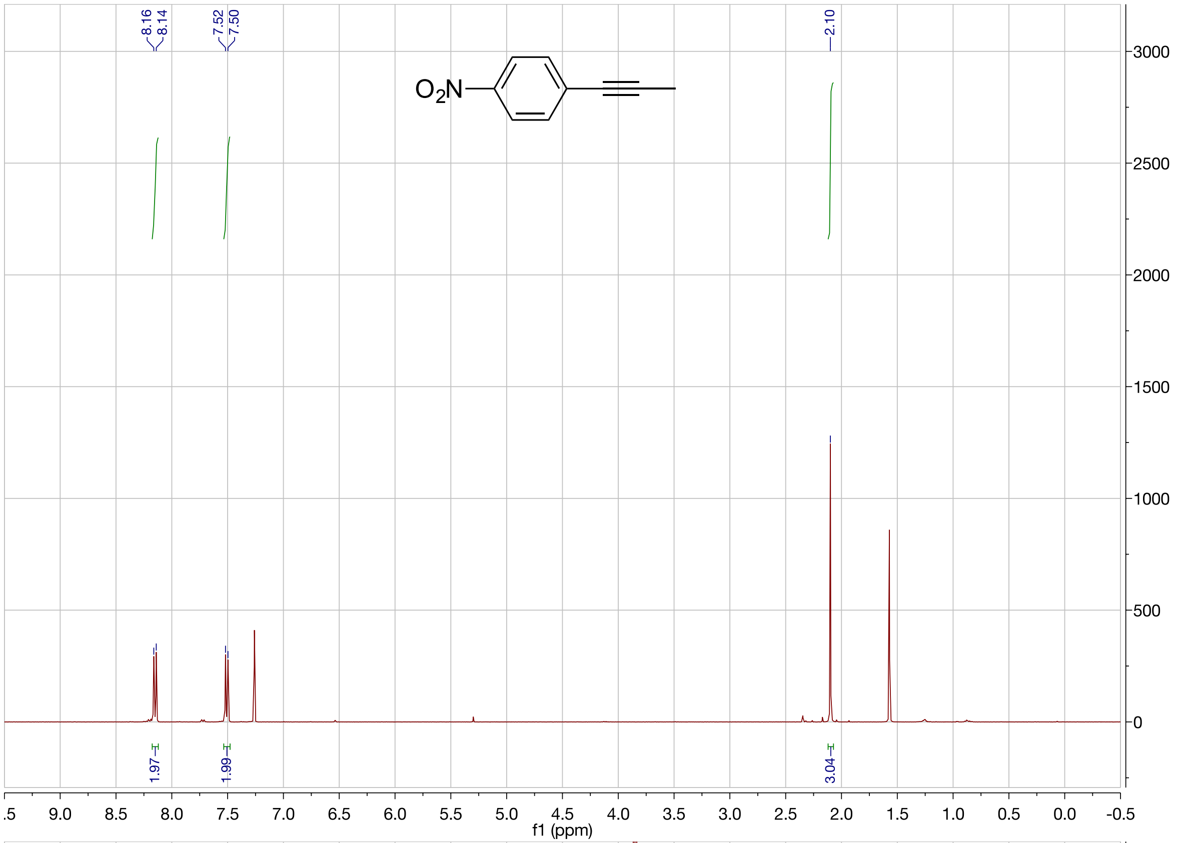
8.16
8.14
7.52
7.50
2.10
O_2N
1.97
1.99
3.04
f1 (ppm)

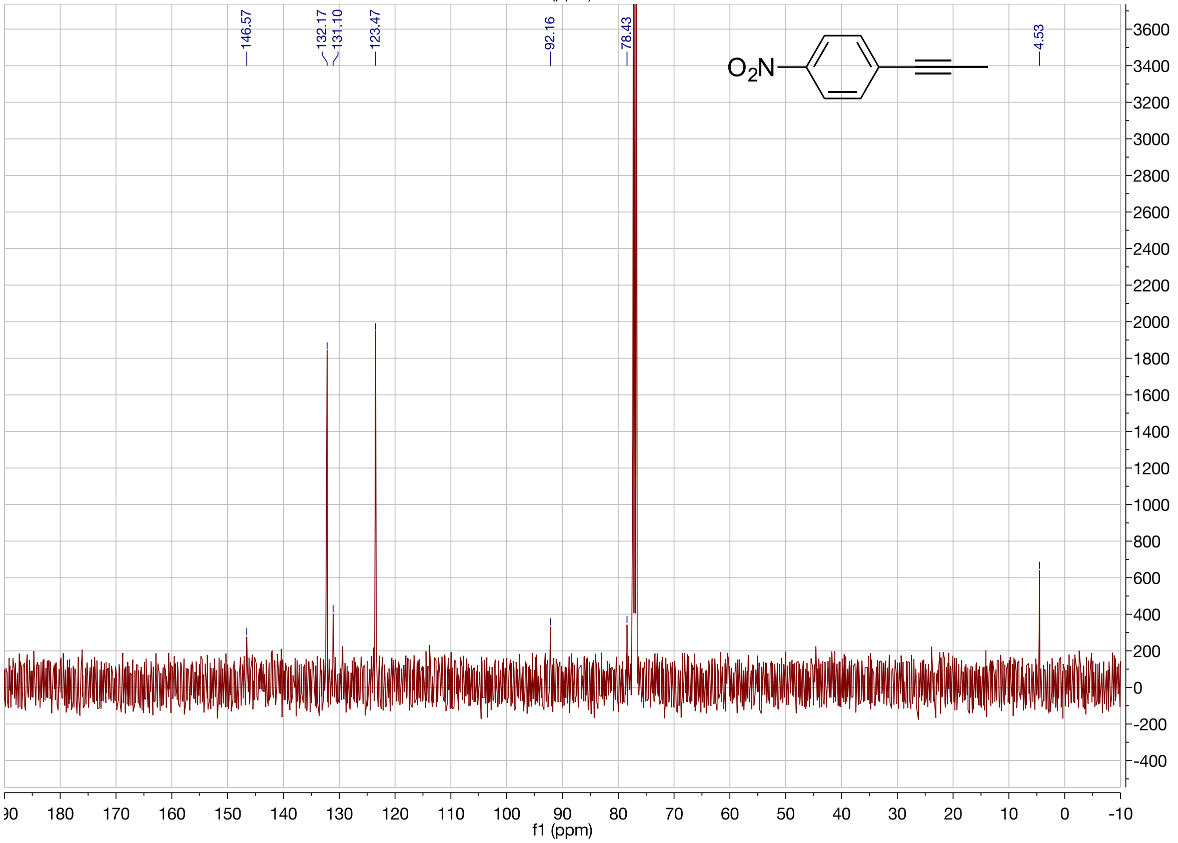
146.57
132.17
131.10
123.47
92.16
78.43
4.53
O_2N
f1 (ppm)

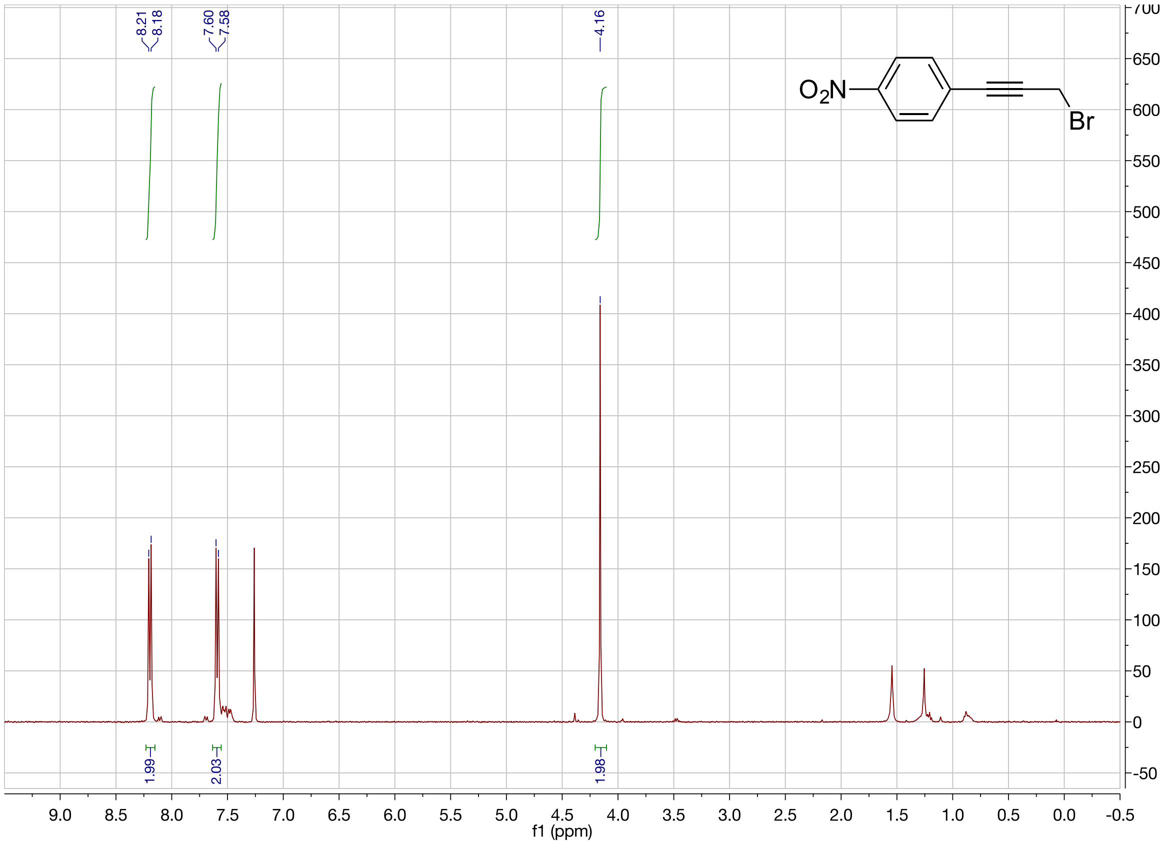
8.21
8.18
7.60
7.58
4.16
O2N
Br
1.99
2.03
1.98
f1 (ppm)

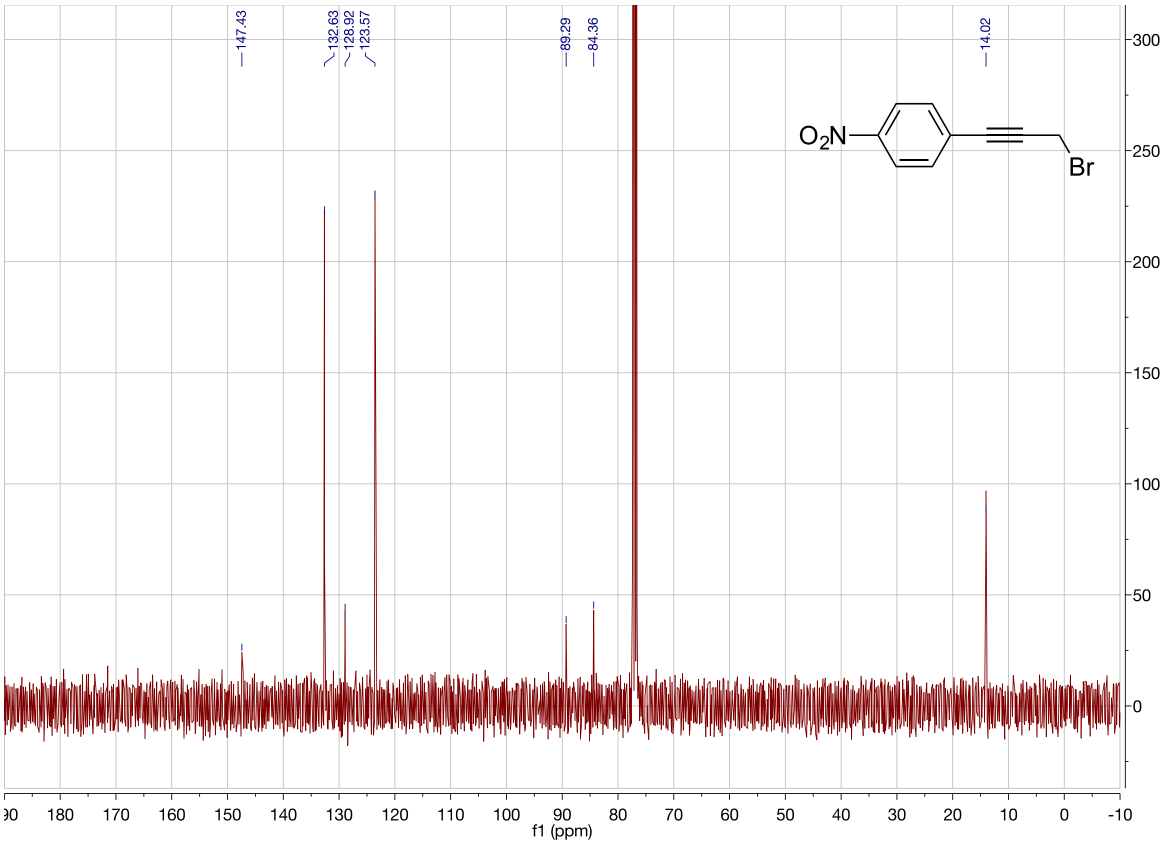
147.43
132.63
128.92
123.57
89.29
84.36
14.02
O2N
Br
f1 (ppm)

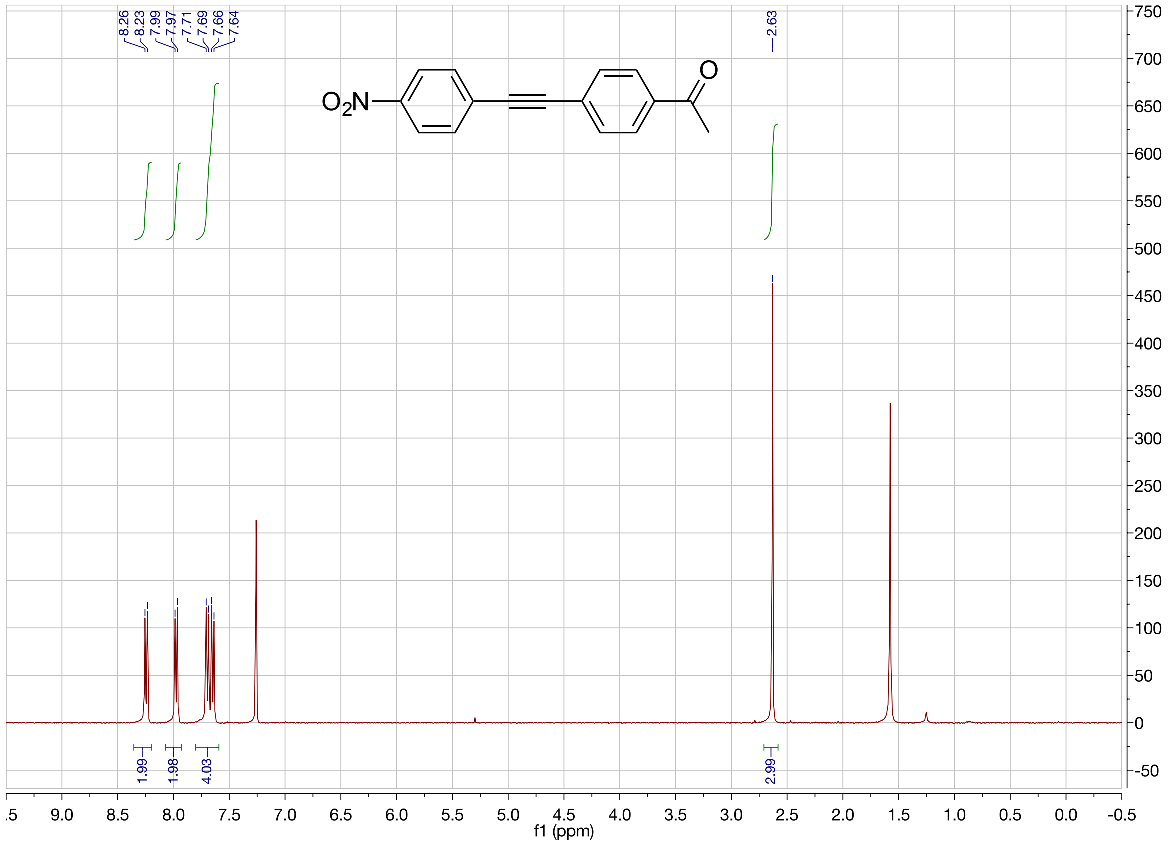
8.26
8.23
7.99
7.97
7.71
7.69
7.66
7.64
2.63
O
O2N
1.99
1.98
4.03
2.99
f1 (ppm)

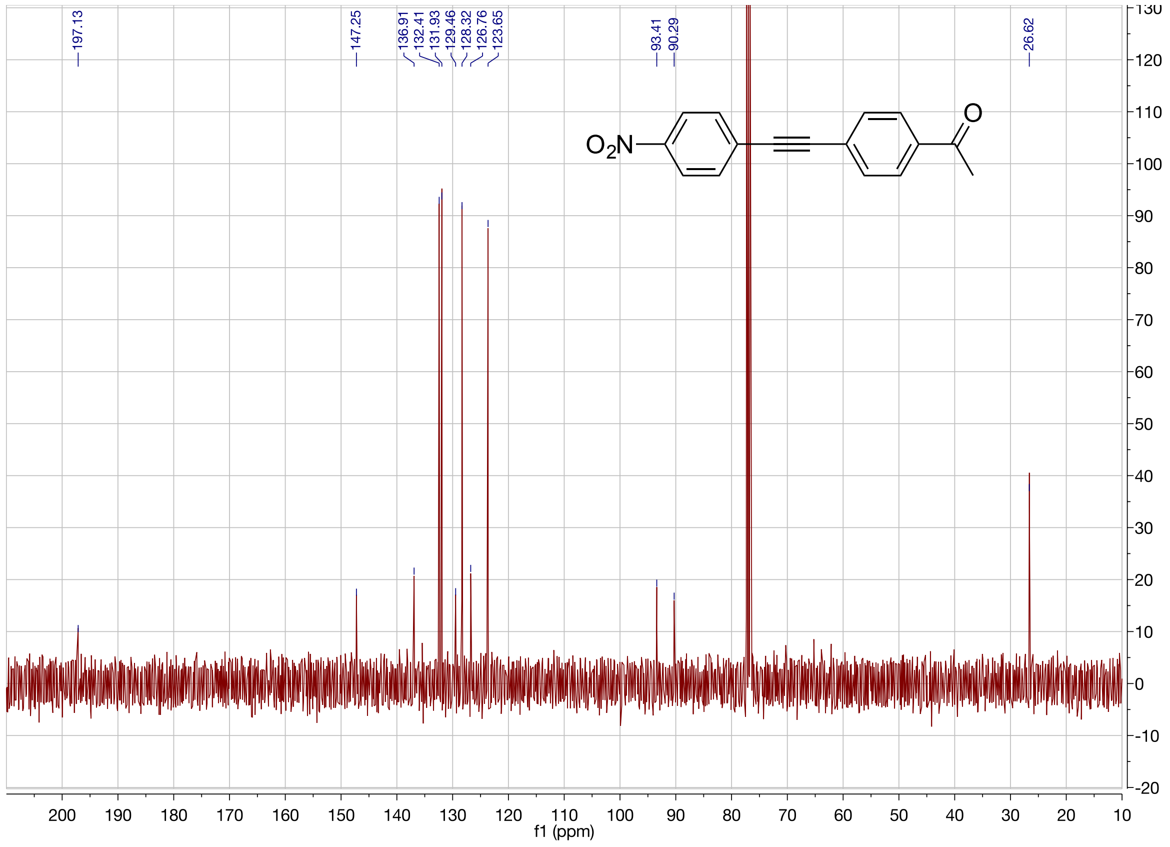
197.13
147.25
136.91
132.41
131.93
129.46
128.32
126.76
123.65
93.41
90.29
26.62
O
O2N
f1 (ppm)

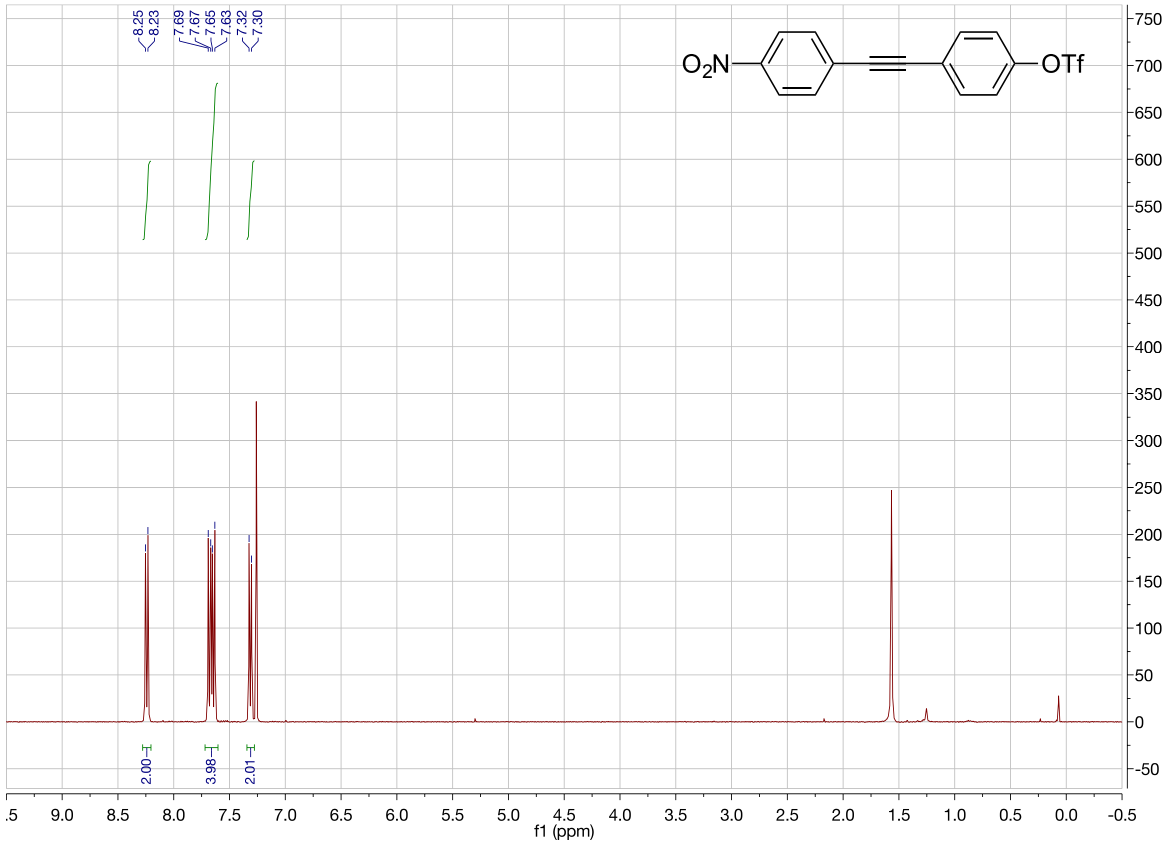
8.25
8.23
7.69
7.67
7.65
7.63
7.32
7.30
O2N
OTf
2.00
3.98
2.01
f1 (ppm)

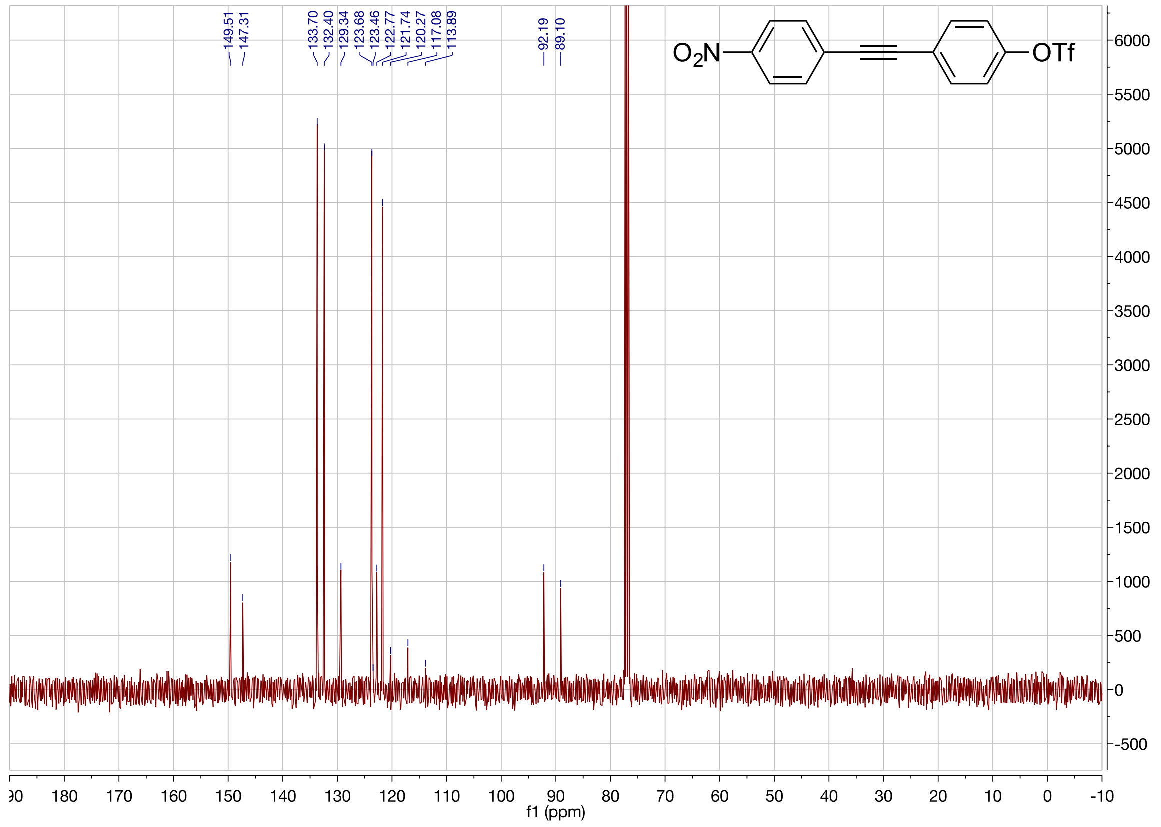
149.51
147.31
133.70
132.40
129.34
123.68
123.46
122.77
121.74
120.27
117.08
113.89
92.19
89.10
O2N
OTf
f1 (ppm)

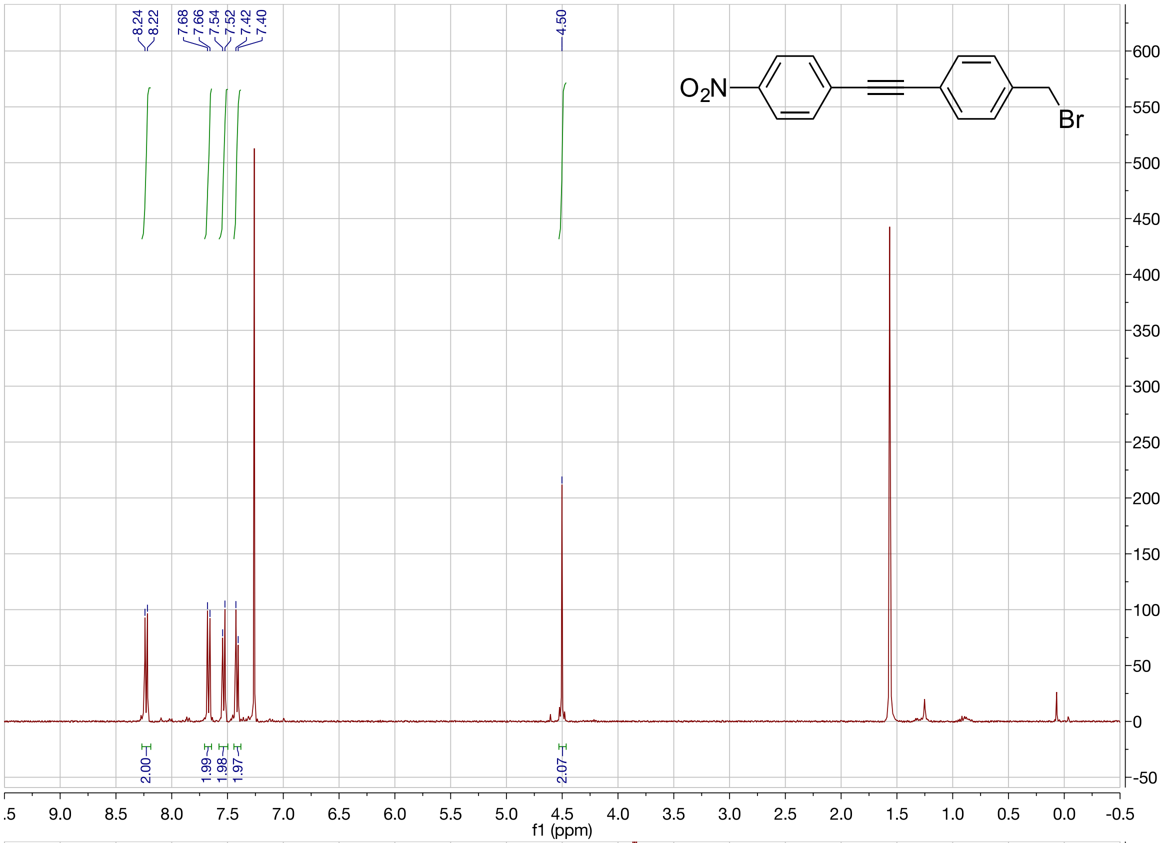
8.24
8.22
7.68
7.66
7.54
7.52
7.42
7.40
4.50
O2N
Br
2.00
1.99
1.98
1.97
2.07
f1 (ppm)

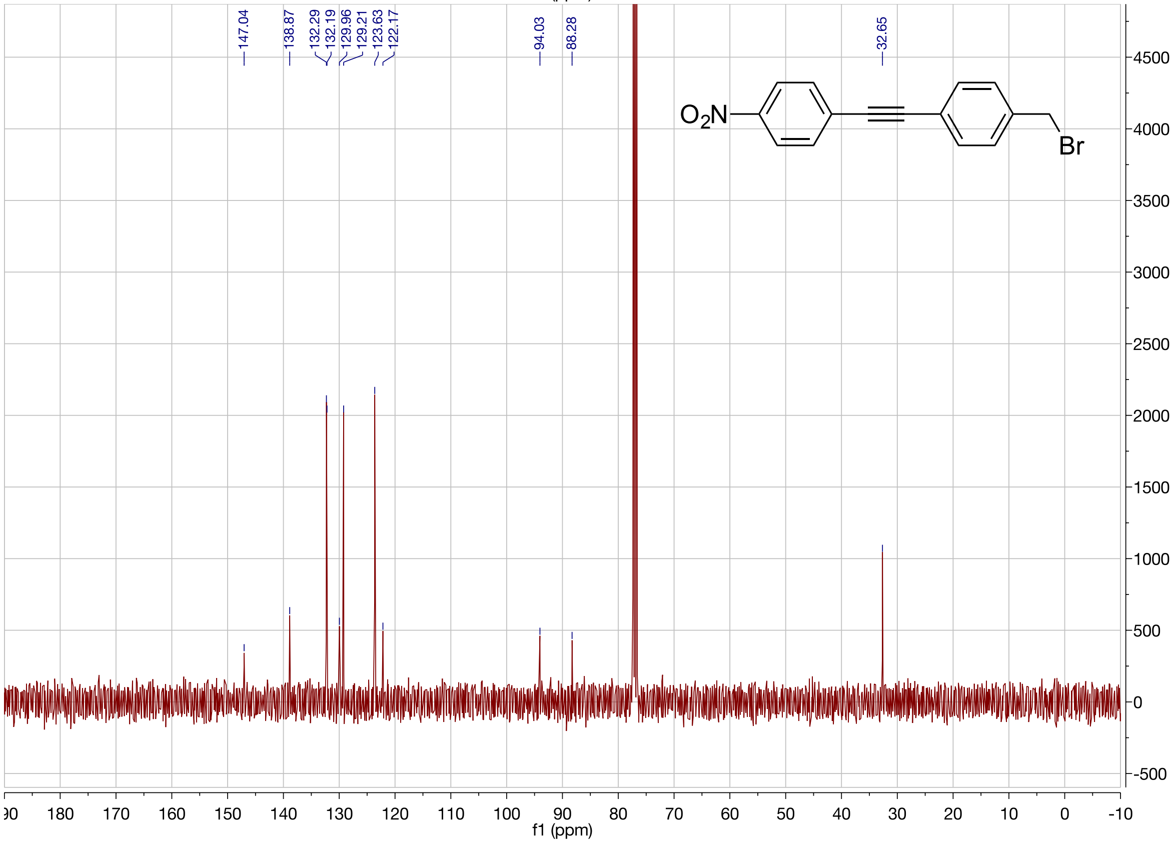
147.04
138.87
132.29
132.19
129.96
129.21
123.63
122.17
94.03
88.28
32.65
O2N
Br
f1 (ppm)

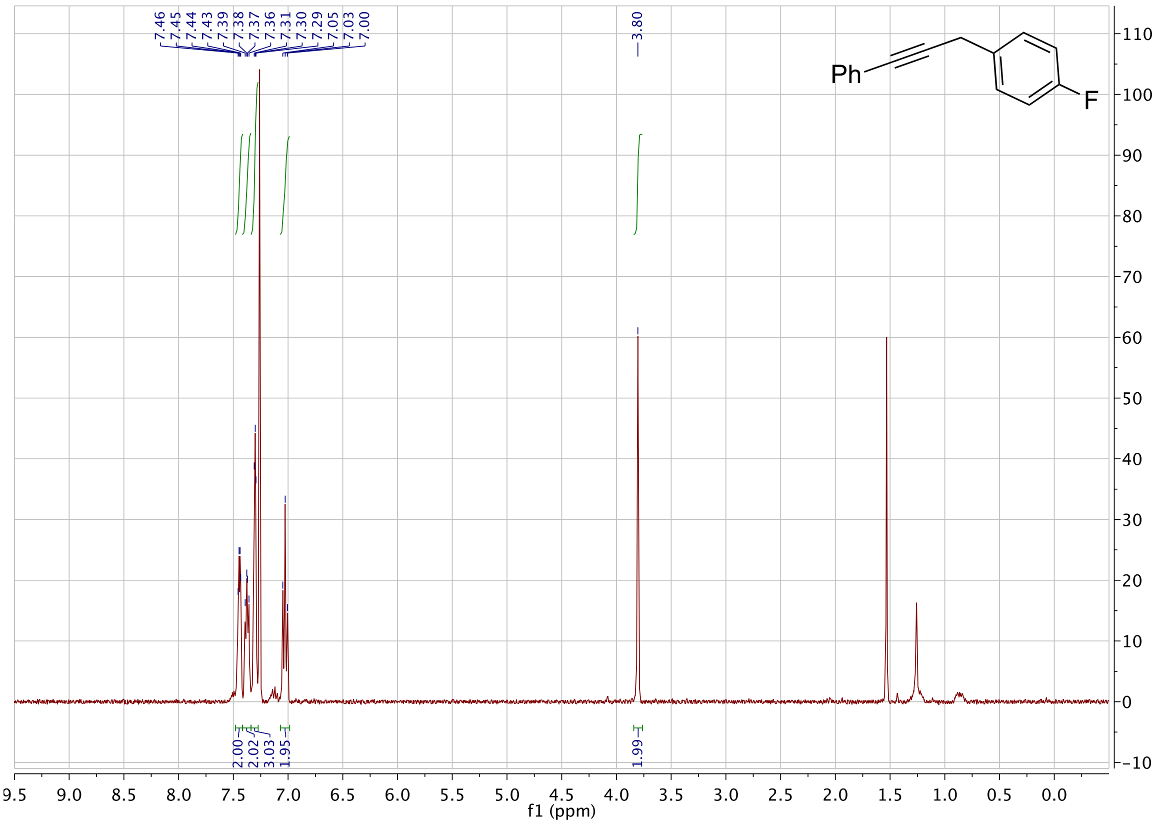
7.46
7.45
7.44
7.43
7.39
7.38
7.37
7.36
7.31
7.30
7.29
7.05
7.03
7.00
3.80
Ph
F
2.00
2.02
3.03
1.95
1.99
f1 (ppm)

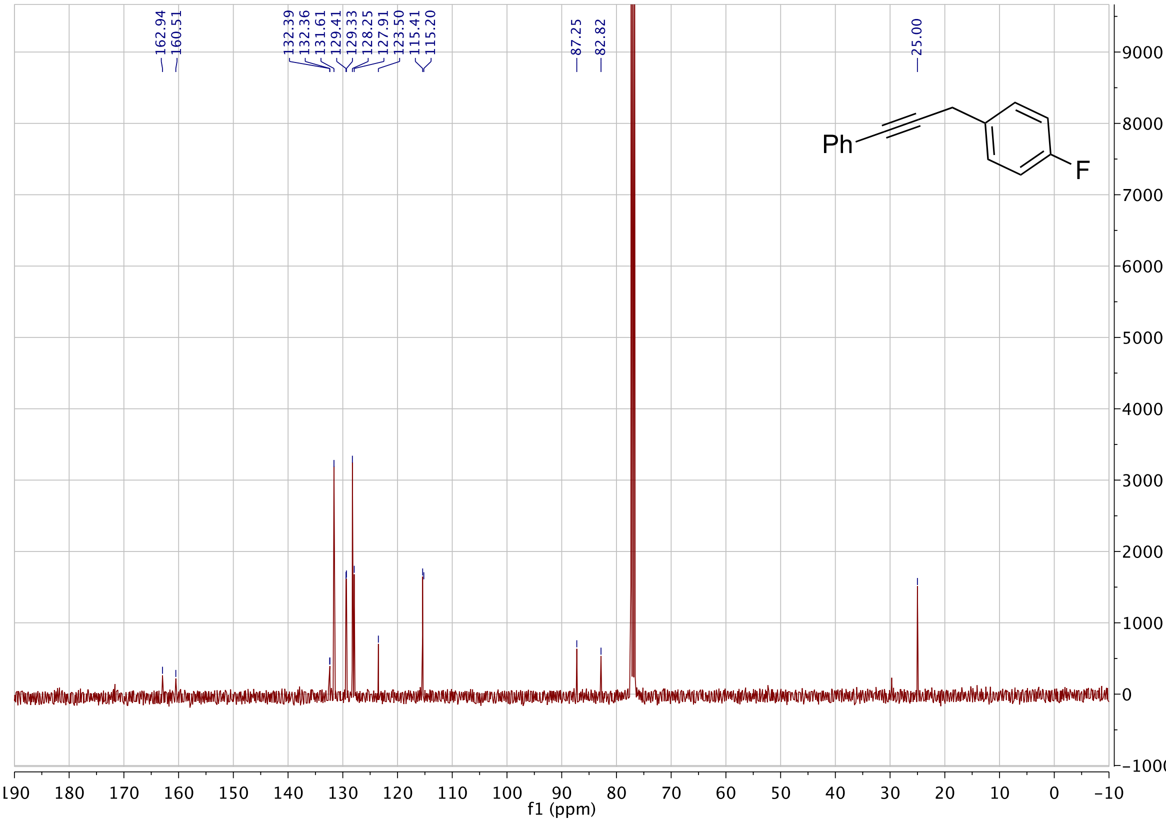
162.94
160.51
132.39
132.36
131.61
129.41
129.33
128.25
127.91
123.50
115.41
115.20
87.25
82.82
25.00
Ph
F
f1 (ppm)

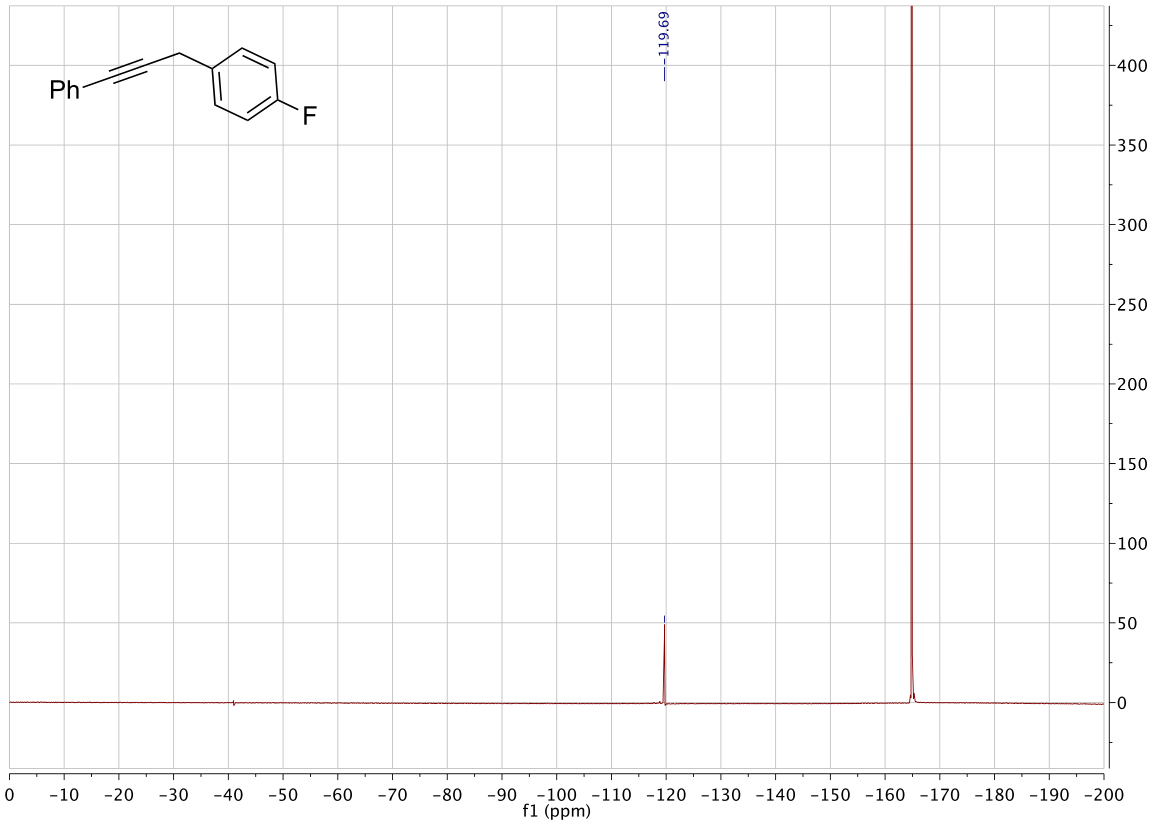

Ph
F
-119.69
400
350
300
250
200
150
100
50
0
0 −10 −20 −30 −40 −50 −60 −70 −80 −90 −100 −110 −120 −130 −140 −150 −160 −170 −180 −190 −200
f1 (ppm)

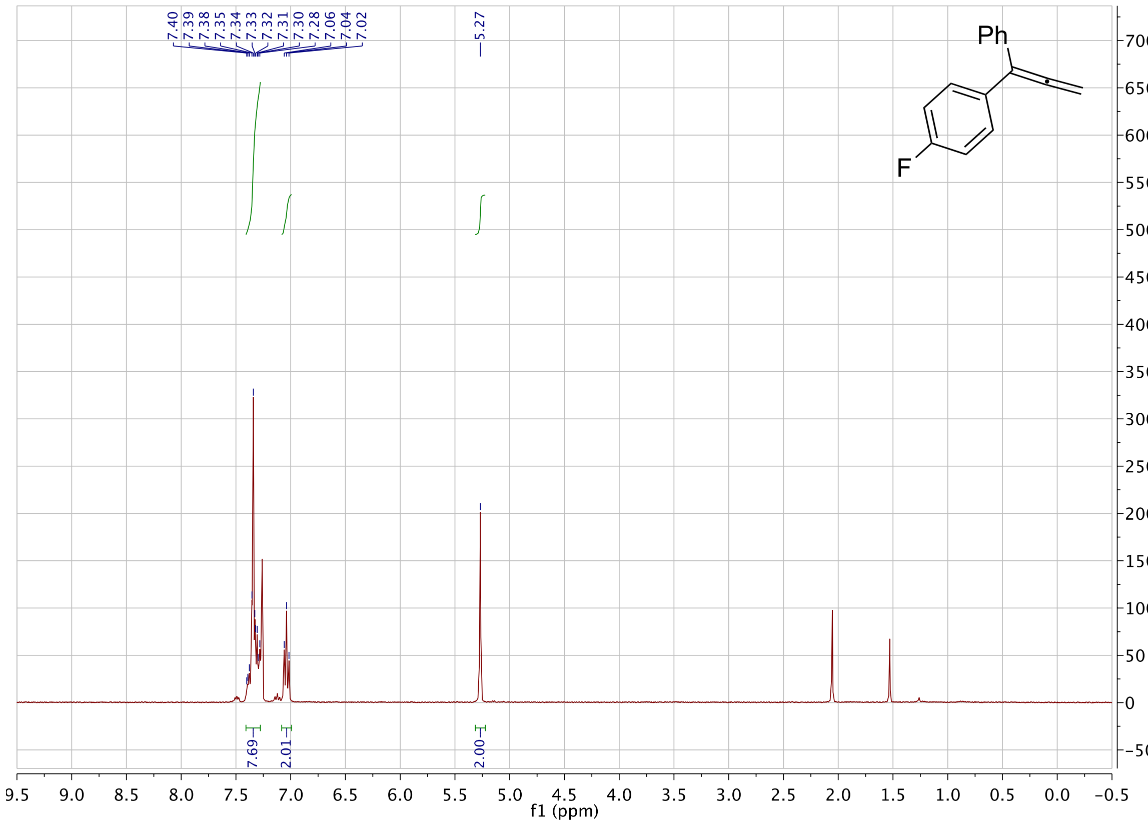

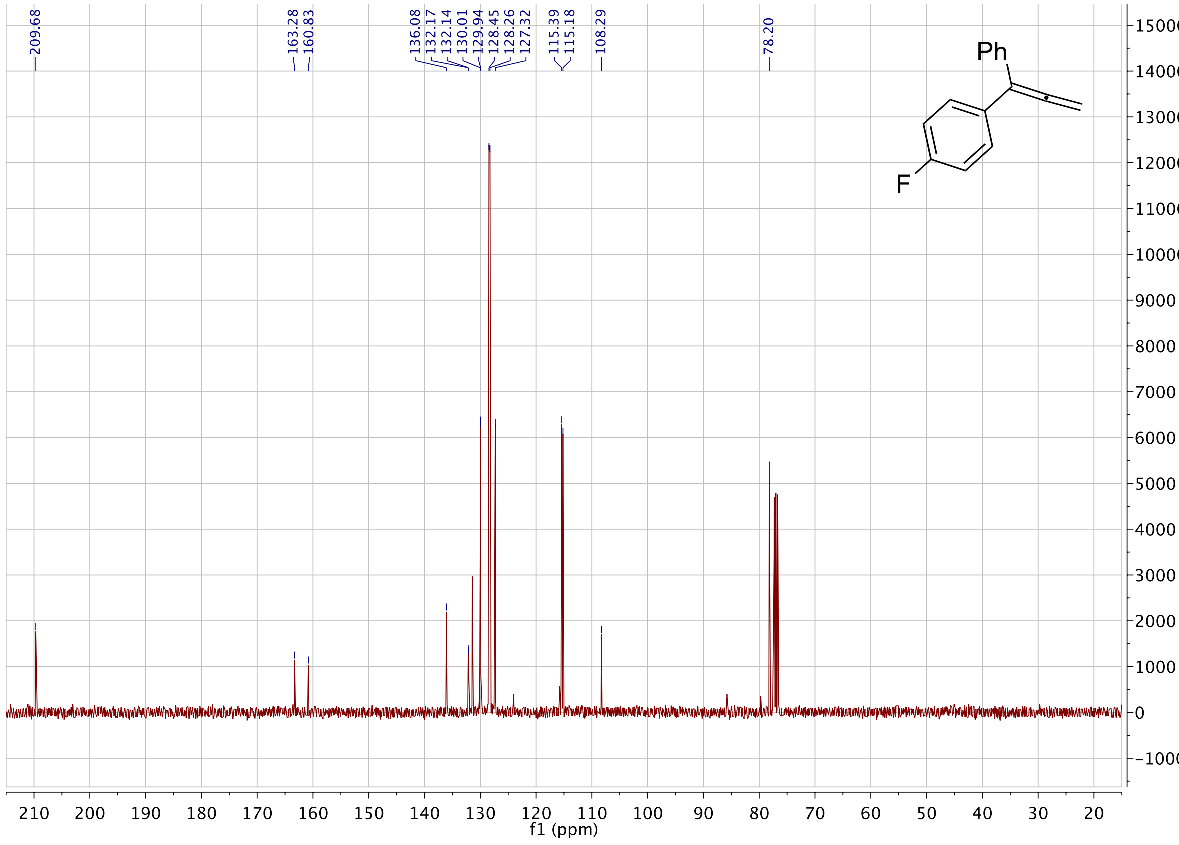

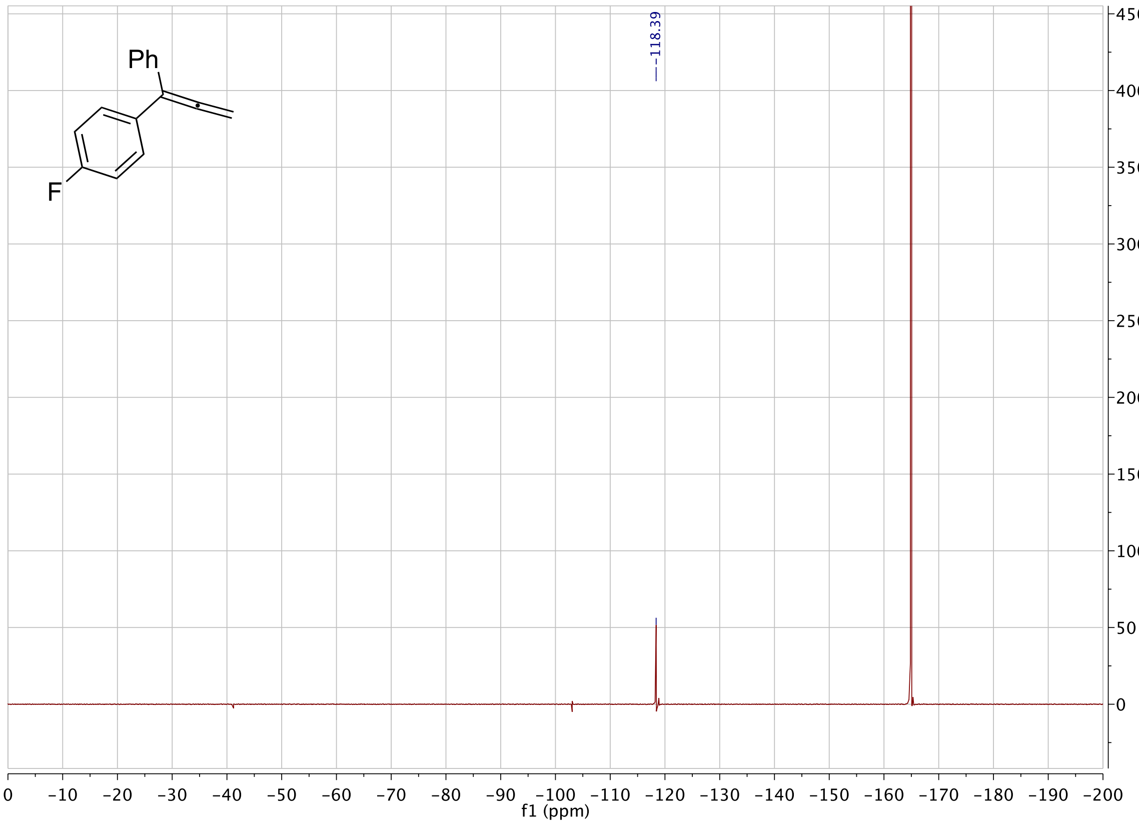
Ph
F
--118.39
450
400
350
300
250
200
150
100
50
0
0 −10 −20 −30 −40 −50 −60 −70 −80 −90 −100 −110 −120 −130 −140 −150 −160 −170 −180 −190 −200
f1 (ppm)

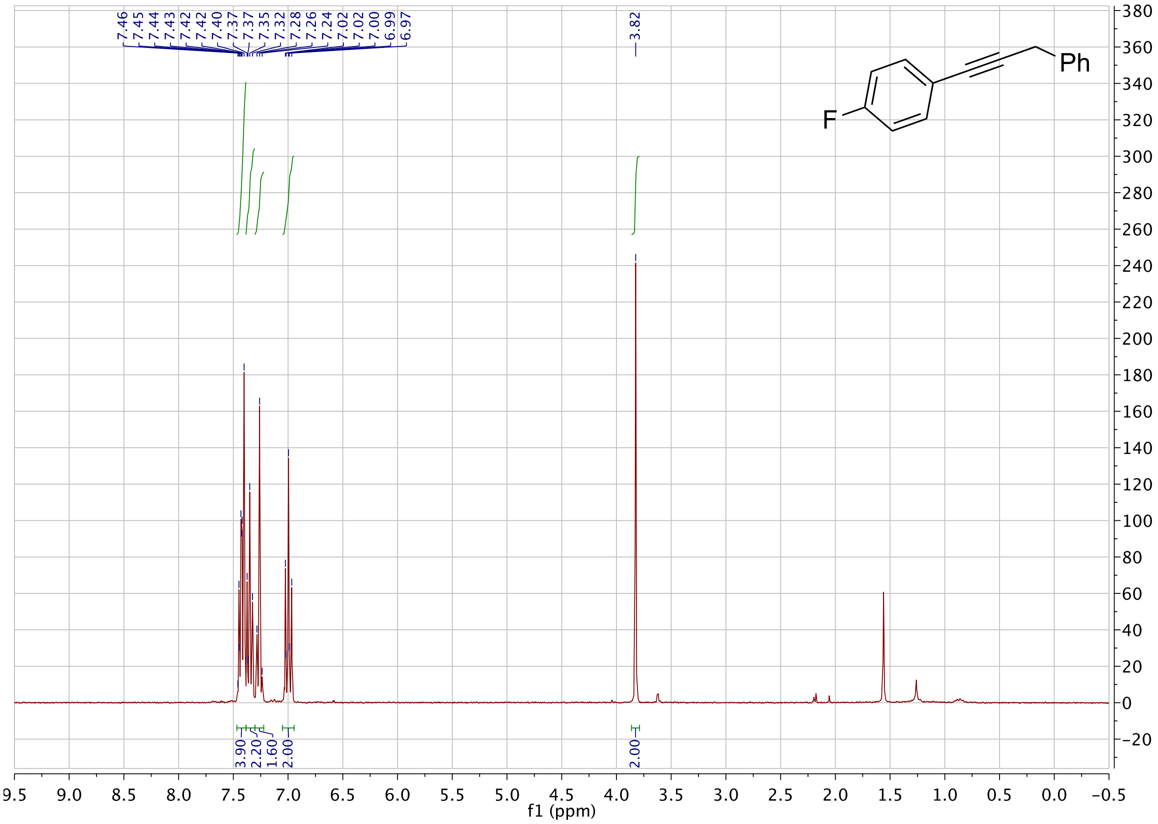
7.46
7.45
7.44
7.43
7.42
7.42
7.40
7.37
7.37
7.35
7.32
7.28
7.26
7.24
7.02
7.02
7.00
6.99
6.97
3.82
F
Ph
3.90
2.20
1.60
2.00
2.00
f1 (ppm)

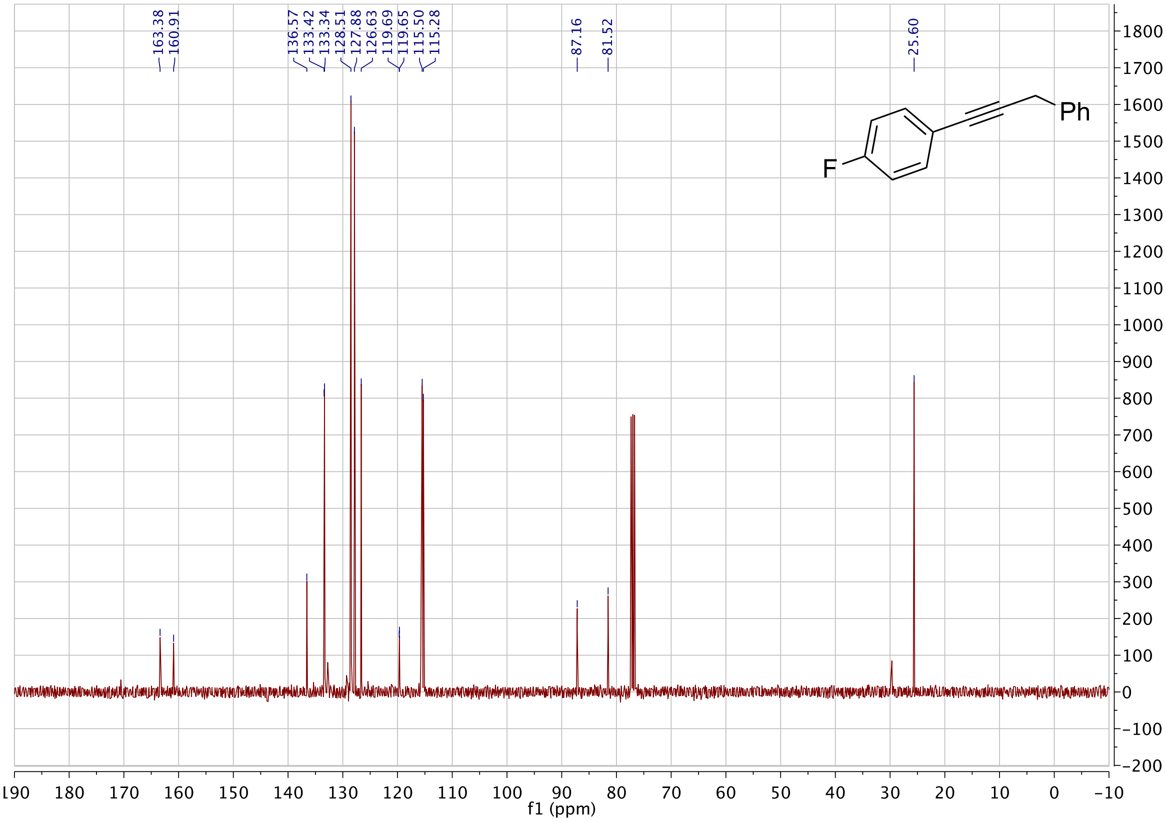
163.38
160.91
136.57
133.42
133.34
128.51
127.88
126.63
119.69
119.65
115.50
115.28
87.16
81.52
25.60
F
Ph
f1 (ppm)

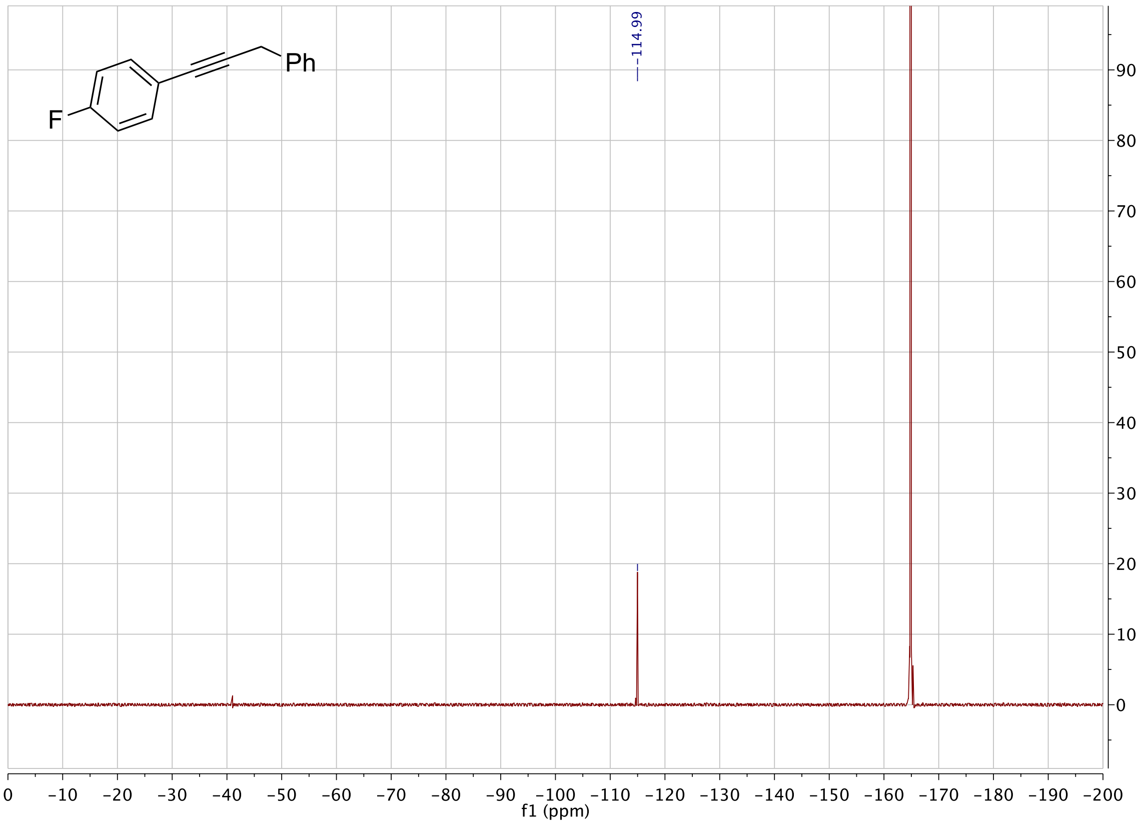

F
Ph
-114.99
f1 (ppm)
